# Supplementary material for: Structure-based discovery of potent and selective melatonin receptor agonists
Source: eLife. 2020 Mar 2;9:e53779. doi: 10.7554/eLife.53779 (PMC7080406; doi:10.7554/eLife.53779)
Supplement: Supplementary file 1. — The table lists vendors invormation, and Tanimo distances to the closest known MT ligands in ChEMBL database. [file elife-53779-supp1.docx]

Supplementary Material for the article

**Structure-Based Discovery of Potent & Selective Melatonin Receptors Agonists**

Nilkanth Patel^†^, Xi-Ping Huang^‡, ∥^, Jessica M. Grandner^†, ^^, Linda C. Johansson^†^, Benjamin Stauch^†^, John D. McCorvy^‡, ∥, &^, Yongfeng Liu^‡, ∥^, Bryan L. Roth^‡, §, ∥^, Vsevolod Katritch^*, †^

*Corresponding Author. Email: [katritch@usc.edu](mailto:katritch@usc.edu) (V.K.)

**This file includes:**

**Table S1.** **List of selected 62 compounds from VLS**

## Table S1. List of selected 62 compounds from VLS (Identified hits’ data shown in bold)

| Compound | Mol. Weight | ZINC id | Vendor | Vendor ID | Purity (≥ %) | VLS model | Distance CHEMBL | Closest CHEMBL |
| --- | --- | --- | --- | --- | --- | --- | --- | --- |
| **1** | 248.1 | ZINC000646611107 | Enamine | Z2228498436 | 90 | MT1 | 0.40 | CHEMBL124577 |
| **2** | 231.1 | ZINC000681274710 | Enamine | Z1725992474 | 95 | MT1 | 0.51 | CHEMBL1823016 |
| **3** | 242.1 | ZINC000546578797 | Enamine | Z1687515356 | 95 | MT1 | 0.58 | CHEMBL8200 |
| **4** | 241.1 | ZINC000298429656 | Enamine | Z2333538374 | 90 | MT1 | 0.46 | CHEMBL54262 |
| **5** | 249.1 | ZINC000289053784 | Enamine | Z2161843026 | 95 | MT1 / MT2 | 0.47 | CHEMBL50311 |
| **6** | 236.1 | ZINC000276156141 | Enamine | Z2252522873 | 95 | MT1 | 0.39 | CHEMBL127596 |
| **7** | 220.1 | ZINC000258964556 | Enamine | Z1551725861 | 90 | MT1 | 0.31 | CHEMBL127596 |
| **8** | 241.1 | ZINC000533004080 | Enamine | Z1480824591 | 90 | MT1 | 0.58 | CHEMBL1823015 |
| **9** | 234.1 | ZINC000664235904 | Enamine | Z2614615475 | 95 | MT1 | 0.69 | CHEMBL1760945 |
| **10** | 226.1 | ZINC000227309310 | Enamine | Z1627633848 | 95 | MT1 | 0.53 | CHEMBL139497 |
| **11** | 219.1 | ZINC000336355552 | Enamine | Z804762324 | 90 | MT1 | 0.41 | CHEMBL137969 |
| **12** | 222.1 | ZINC000413601342 | Enamine | Z2348423828 | 95 | MT1 | 0.53 | CHEMBL139497 |
| **13** | 192.1 | ZINC000647635198 | Enamine | Z2445988651 | 95 | MT1 | 0.61 | CHEMBL60001 |
| **14** | 239.1 | ZINC000090284709 | Enamine | Z1981813878 | 95 | MT1 | 0.43 | CHEMBL1092047 |
| **15** | 202.1 | ZINC000163280593 | Enamine | Z1618072048 | 95 | MT1 | 0.62 | CHEMBL395437 |
| **16** | 245.1 | ZINC000048543543 | Enamine | Z640201342 | 95 | MT1 | 0.58 | CHEMBL8094 |
| **17** | 233.1 | ZINC000185901046 | Enamine | Z1729375648 | 95 | MT1 | 0.51 | CHEMBL4105125 |
| **18** | 235.1 | ZINC000646561028 | Enamine | Z2228267762 | 95 | MT1 | 0.39 | CHEMBL8032 |
| **19** | 247.1 | ZINC000684533317 | Enamine | Z1955032980 | 95 | MT2 | 0.63 | CHEMBL216158 |
| **20** | 241.1 | ZINC000086778271 | Enamine | Z1343343581 | 95 | MT2 | 0.58 | CHEMBL139497 |
| **21** | **224.1** | **ZINC000574396478** | **Enamine** | **Z2434357410** | **95** | **MT2** | **0.50** | **CHEMBL237236** |
| **22** | 236.1 | ZINC000635555148 | Enamine | Z2799987596 | 95 | MT2 | 0.24 | CHEMBL297947 |
| **23** | **248.1** | **ZINC000636043315** | **Enamine** | **Z2801662476** | **95** | **MT2** | **0.22** | **CHEMBL301083** |
| **24** | 233.1 | ZINC000571358399 | Enamine | Z2143496175 | 95 | MT2 | 0.65 | CHEMBL1091894 |
| **25** | 231.1 | ZINC000425262799 | Enamine | Z2409717456 | 95 | MT2 | 0.60 | CHEMBL268165 |
| **26** | 249.1 | ZINC000685911113 | Enamine | Z2179535626 | 95 | MT1 / MT2 | 0.61 | CHEMBL1091894 |
| **27** | 232.1 | ZINC000450719435 | Enamine | Z2273688472 | 95 | MT2 | 0.66 | CHEMBL1760947 |
| **28** | **233.1** | **ZINC000000290474** | **Enamine** | **Z1196462057** | **95** | **MT2** | **0.05** | **CHEMBL137949** |
| **29** | **248.2** | **ZINC000624768199** | **Enamine** | **Z2701721005** | **95** | **MT2** | **0.43** | **CHEMBL139497** |
| **30** | 241.1 | ZINC000335753637 | Enamine | Z1959154430 | 95 | MT2 | 0.69 | CHEMBL4087265 |
| **31** | 232.1 | ZINC000177805562 | Enamine | Z1337812751 | 95 | MT2 | 0.53 | CHEMBL395437 |
| **32** | 245.1 | ZINC000337312179 | Enamine | Z1499411301 | 95 | MT2 | 0.50 | CHEMBL219127 |
| **33** | 245.1 | ZINC000338047573 | Enamine | Z1005838414 | 95 | MT2 | 0.55 | CHEMBL336960 |
| **34** | 228.1 | ZINC000007069489 | Enamine | Z108630396 | 95 | MT2 | 0.52 | CHEMBL268165 |
| **35** | 233.1 | ZINC000292733495 | Enamine | Z2212371816 | 95 | MT2 | 0.56 | CHEMBL3580913 |
| **36** | 237.1 | ZINC000074859414 | Enamine | Z1203061587 | 95 | MT2 | 0.51 | CHEMBL1823017 |
| **37** | **245.1** | **ZINC000265572185** | **Enamine** | **Z1230796252** | **95** | **MT2** | **0.57** | **CHEMBL3580913** |
| **38** | 246.1 | ZINC000020354491 | MolPort | MolPort-000-900-380 | 90 | MT1 / MT2 | 0.64 | CHEMBL64664 |
| **39** | 223.1 | ZINC000049538518 | MolPort | MolPort-009-652-706 | 90 | MT1 / MT2 | 0.70 | CHEMBL376693 |
| **40** | 242.1 | ZINC000073658806 | MolPort | MolPort-021-780-191 | 95 | MT1 | 0.63 | CHEMBL329979 |
| **41** | 234.1 | ZINC000072323996 | MolPort | MolPort-019-952-828 | 90 | MT1 / MT2 | 0.69 | CHEMBL237840 |
| **42** | 247.1 | ZINC000002528737 | MolPort | MolPort-002-498-254 | 90 | MT1 | 0.59 | CHEMBL1093457 |
| **43** | 239.1 | ZINC000000273619 | MolPort | MolPort-000-510-506 | 90 | MT1 | 0.60 | CHEMBL273624 |
| **44** | **247.1** | **ZINC000001452754** | **MolPort** | **MolPort-003-861-713** | **95** | **MT1 / MT2** | **0.59** | **CHEMBL395437** |
| **45** | **232.1** | **ZINC000000087930** | **MolPort** | **MolPort-000-933-891** | **90** | **MT1** | **0.59** | **CHEMBL140785** |
| **46** | 222.1 | ZINC000000154679 | MolPort | MolPort-001-572-860 | 95 | MT1 | 0.33 | CHEMBL54198 |
| **47** | **248.0** | **ZINC000002508551** | **MolPort** | **MolPort-002-881-836** | **90** | **MT1 / MT2** | **0.60** | **CHEMBL288562** |
| **48** | 227.1 | ZINC000000274667 | MolPort | MolPort-001-969-774 | 90 | MT1 | 0.60 | CHEMBL14635 |
| **49** | 248.1 | ZINC000020354470 | MolPort | MolPort-000-900-367 | 90 | MT1 / MT2 | 0.60 | CHEMBL395437 |
| **50** | 218.1 | ZINC000020354460 | MolPort | MolPort-000-900-362 | 90 | MT1 / MT2 | 0.69 | CHEMBL237840 |
| **51** | 236.1 | ZINC000020354478 | MolPort | MolPort-000-900-371 | 90 | MT1 / MT2 | 0.69 | CHEMBL235328 |
| **52** | 231.1 | ZINC000000140934 | MolPort | MolPort-002-713-448 | 90 | MT2 | 0.69 | CHEMBL96173 |
| **53** | 242.1 | ZINC000005773134 | MolPort | MolPort-000-773-637 | 90 | MT2 | 0.69 | CHEMBL243002 |
| **54** | **220.1** | **ZINC000000530046** | **MolPort** | **MolPort-000-928-790** | **90** | **MT2** | **0.43** | **CHEMBL390944** |
| **55** | 216.1 | ZINC000052508006 | MolPort | MolPort-009-196-070 | 95 | MT1 / MT2 | 0.55 | CHEMBL1092647 |
| **56** | 245.1 | ZINC000004754067 | MolPort | MolPort-002-740-778 | 90 | MT2 | 0.63 | CHEMBL8200 |
| **57** | **232.1** | **ZINC000006584926** | **MolPort** | **MolPort-023-197-616** | **95** | **MT2** | **0.53** | **CHEMBL125081** |
| **58** | 232.1 | ZINC000000419982 | MolPort | MolPort-001-987-293 | 90 | MT1 / MT2 | 0.45 | CHEMBL8094 |
| **59** | 245.1 | ZINC000018137901 | ChemBridge | 5730112 | 90 | MT2 | 0.57 | CHEMBL376693 |
| **60** | 231.1 | ZINC000001287292 | ChemBridge | 9026858 | 90 | MT1 / MT2 | 0.64 | CHEMBL215252 |
| **61** | 231.1 | ZINC000000205968 | ChemBridge | 7702622 | 90 | MT2 | 0.68 | CHEMBL376693 |
| **62** | **247.1** | **ZINC000002879048** | **ChemBridge** | **7964675** | **90** | **MT1 / MT2** | **0.64** | **CHEMBL218808** |
