# Supplementary material for: Structure-based discovery of potent and selective melatonin receptor agonists
Source: eLife. 2020 Mar 2;9:e53779. doi: 10.7554/eLife.53779 (PMC7080406; doi:10.7554/eLife.53779)
Supplement: Supplementary file 2. [file elife-53779-supp2.zip › mt_vls_62_compounds_QC_data/Compound_13_Z2445988651/Z2445988651_21482021.PDF]

MaxPeak: 97.76%
Ret_Time: 0.708 min

L693620$1

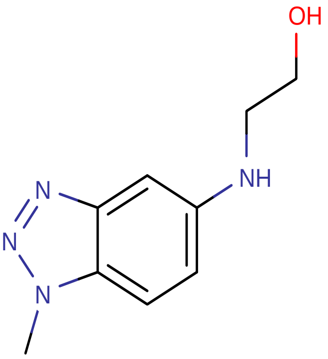

Mol Wt 192.22

Exact Mass 192.11

#	Time	Area%
1	0.708	97.76
2	1.088	1.06
3	1.512	1.18

DAD1 A, Sig=215,16 Ref=off (D:\WORK\D\03\03_02\L084394D\011-D2B-A10-L693620$1.D)

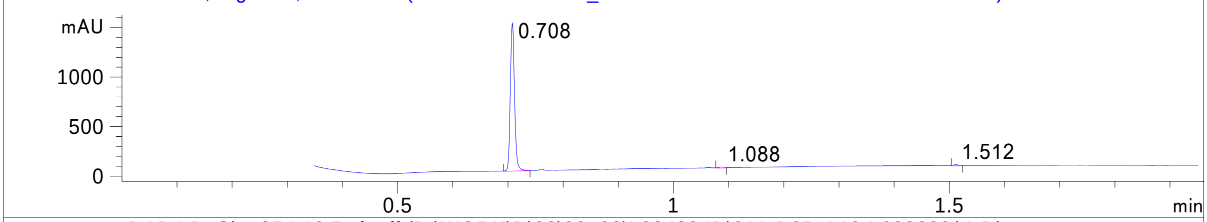

DAD1 B, Sig=254,16 Ref=off (D:\WORK\D\03\03_02\L084394D\011-D2B-A10-L693620$1.D)

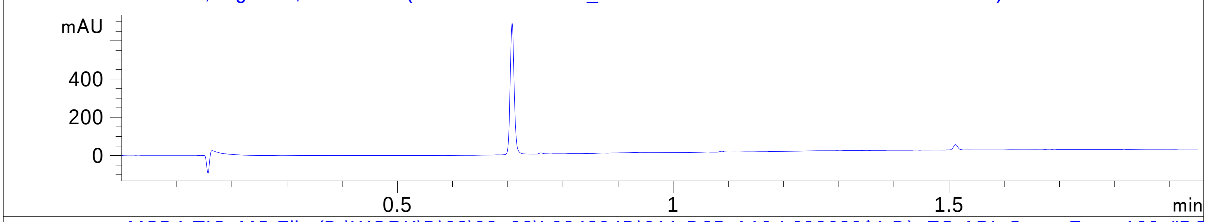

MSD1 TIC, MS File (D:\WORK\D\03\03_02\L084394D\011-D2B-A10-L693620$1.D) ES-API, Scan, Frag: 100, "PO

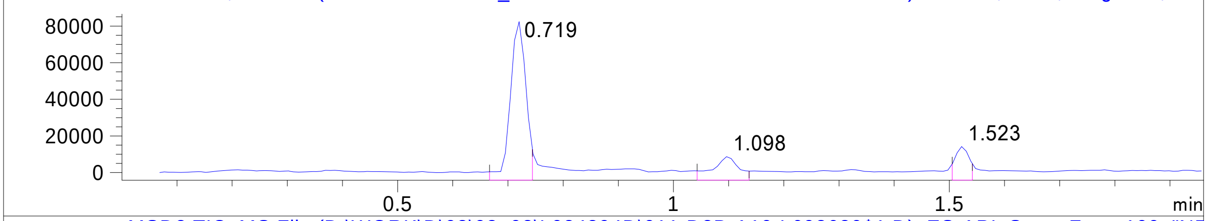

MSD2 TIC, MS File (D:\WORK\D\03\03_02\L084394D\011-D2B-A10-L693620$1.D) ES-API, Scan, Frag: 100, "NE

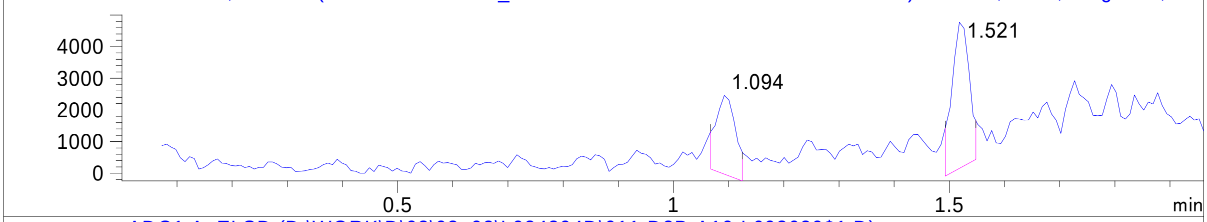

ADC1 A, ELSD (D:\WORK\D\03\03_02\L084394D\011-D2B-A10-L693620$1.D)

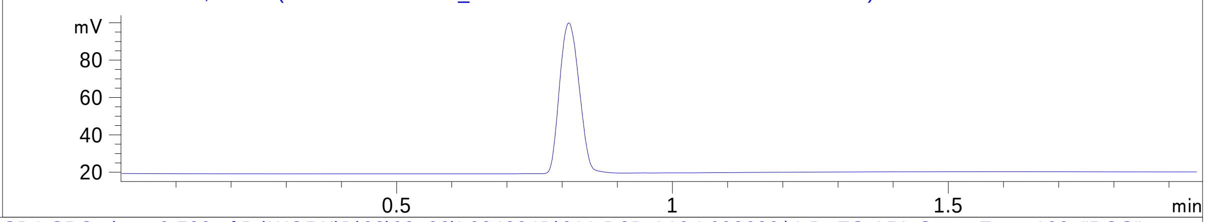

*MSD1 SPC, time=0.720 of D:\WORK\D\03\03_02\L084394D\011-D2B-A10-L693620$1.D ES-API, Scan, Frag: 100, "POS"

RT 0.719

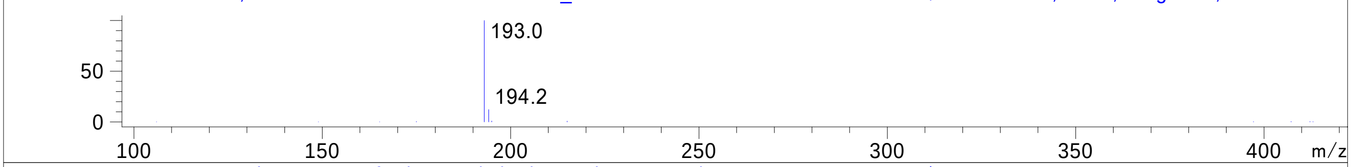

*MSD1 SPC, time=1.096 of D:\WORK\D\03\03_02\L084394D\011-D2B-A10-L693620$1.D ES-API, Scan, Frag: 100, "POS"

RT 1.098

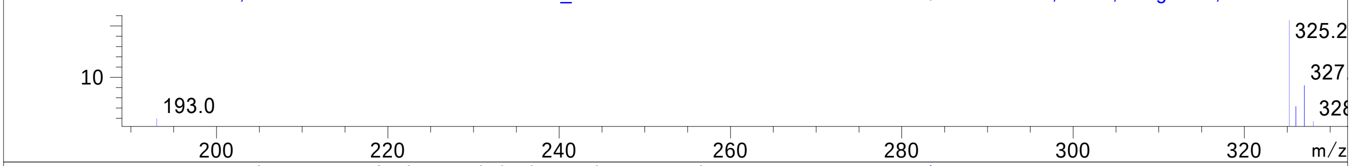

*MSD1 SPC, time=1.522 of D:\WORK\D\03\03_02\L084394D\011-D2B-A10-L693620$1.D ES-API, Scan, Frag: 100, "POS"

RT 1.523

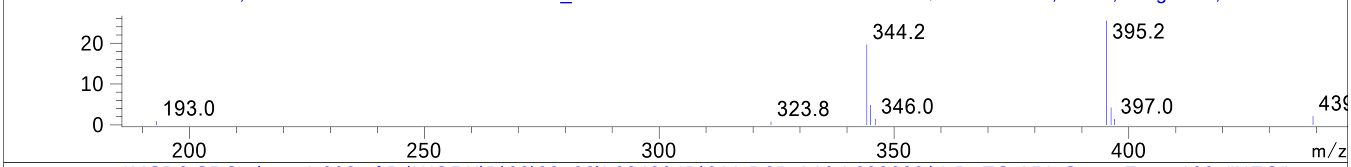

*MSD2 SPC, time=1.092 of D:\WORK\D\03\03_02\L084394D\011-D2B-A10-L693620$1.D ES-API, Scan, Frag: 100, "NEG"

RT 1.094

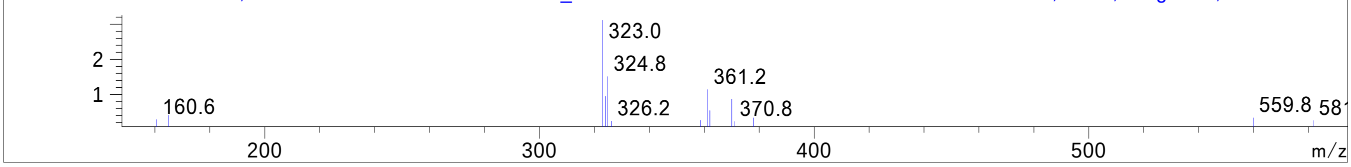

Inj.Date 3/2/2018 K <invalid> -6- Acq. Method C:\Chem32\-> ->

RT 1.521

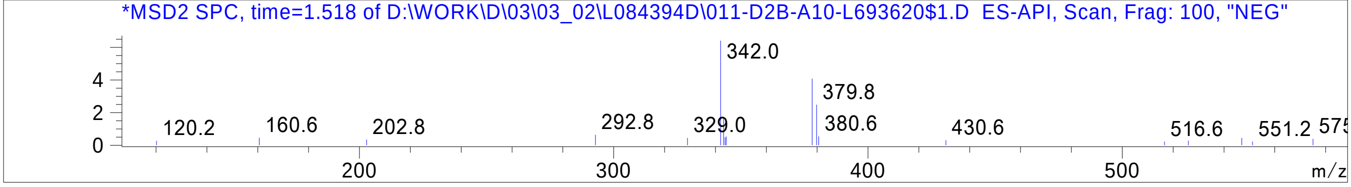
*MSD2 SPC, time=1.518 of D:\WORK\D\03\03_02\L084394D\011-D2B-A10-L693620$1.D ES-API, Scan, Frag: 100, "NEG"
342.0
379.8
380.6
120.2
160.6
202.8
292.8
329.0
430.6
516.6
551.2
575
0
2
4
200
300
400
500
m/z
