# Supplementary material for: Structure-based discovery of potent and selective melatonin receptor agonists
Source: eLife. 2020 Mar 2;9:e53779. doi: 10.7554/eLife.53779 (PMC7080406; doi:10.7554/eLife.53779)
Supplement: Supplementary file 2. [file elife-53779-supp2.zip › mt_vls_62_compounds_QC_data/Compound_16_Z640201342/Z640201342_21507695.PDF]

MaxPeak: 100.00%
Ret_Time: 0.776 min

L693639$2

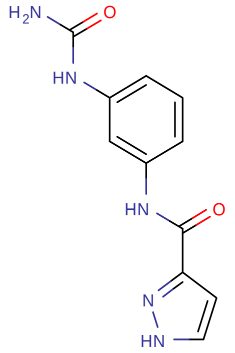

Mol Wt 245.24

Exact Mass 245.09

#	Time	Area%
1	0.776	100.00

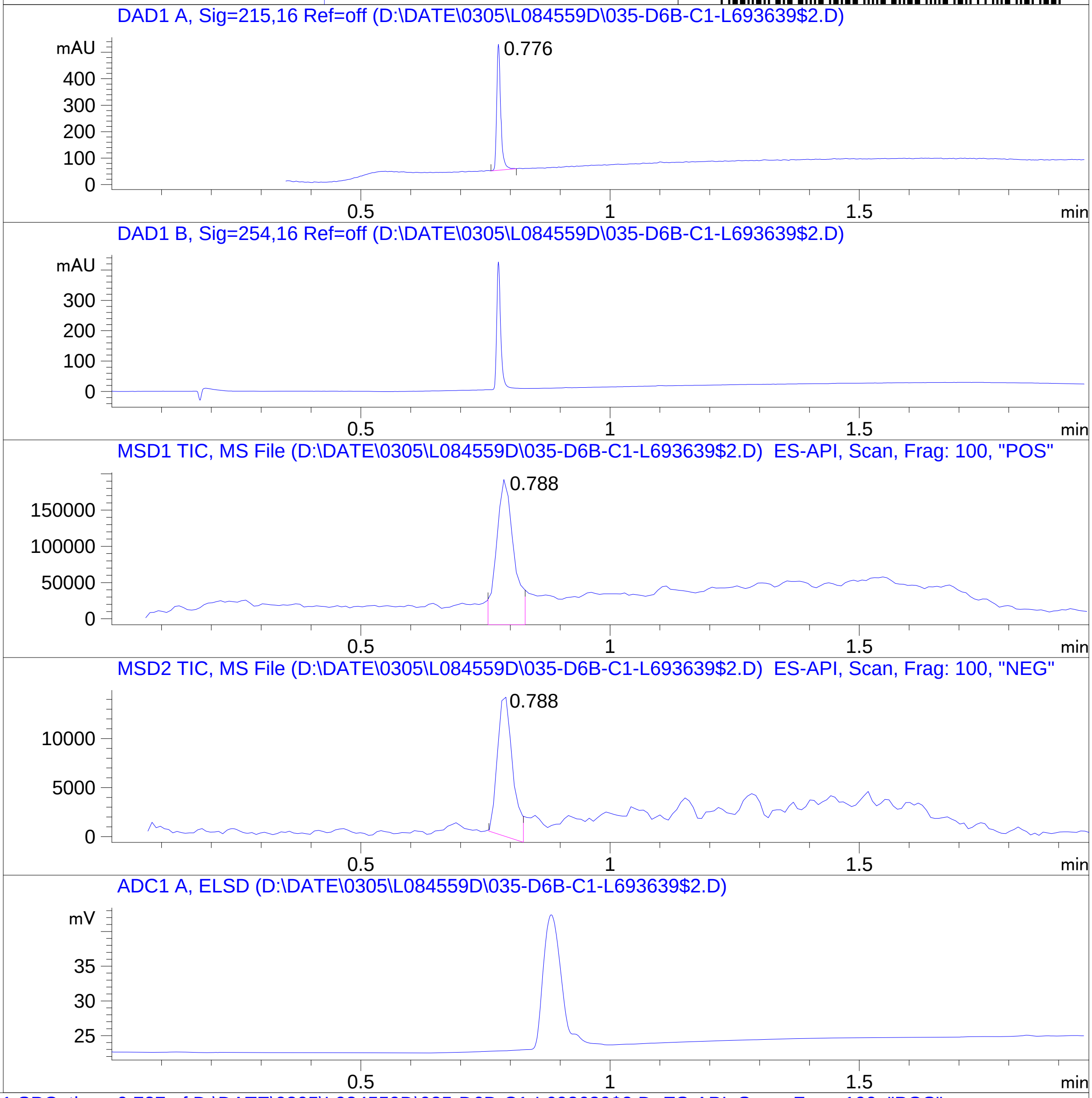

RT 0.788

RT 0.788

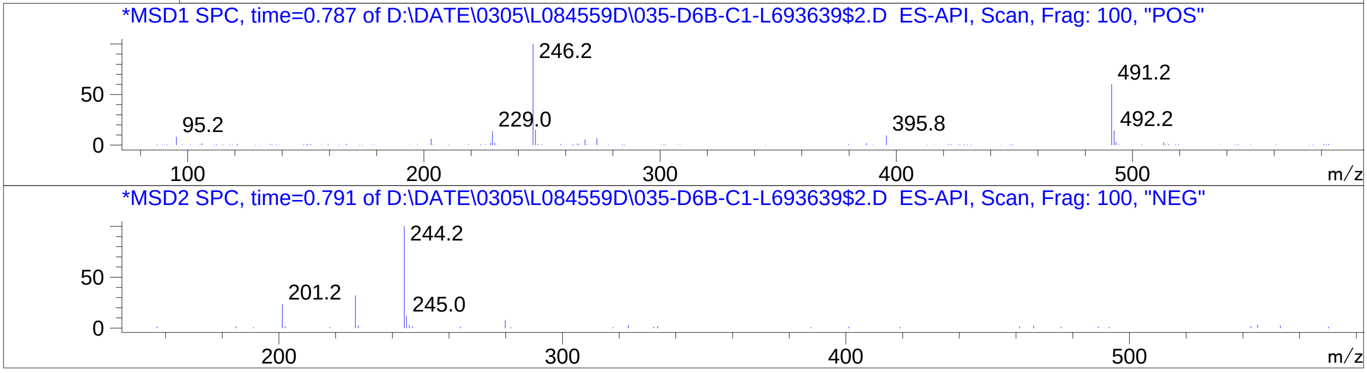

Inj.Date 3/5/2018 E <invalid> -> Acq. Method C:\Chem32\-> ->
