# Supplementary material for: Structure-based discovery of potent and selective melatonin receptor agonists
Source: eLife. 2020 Mar 2;9:e53779. doi: 10.7554/eLife.53779 (PMC7080406; doi:10.7554/eLife.53779)

MaxPeak: 97.66%
Ret_Time: 0.978 min

L693624$3

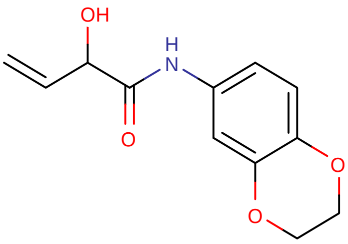

Mol Wt 235.24

Exact Mass 235.09

#	Time	Area%
1	0.978	97.66
2	1.165	2.34

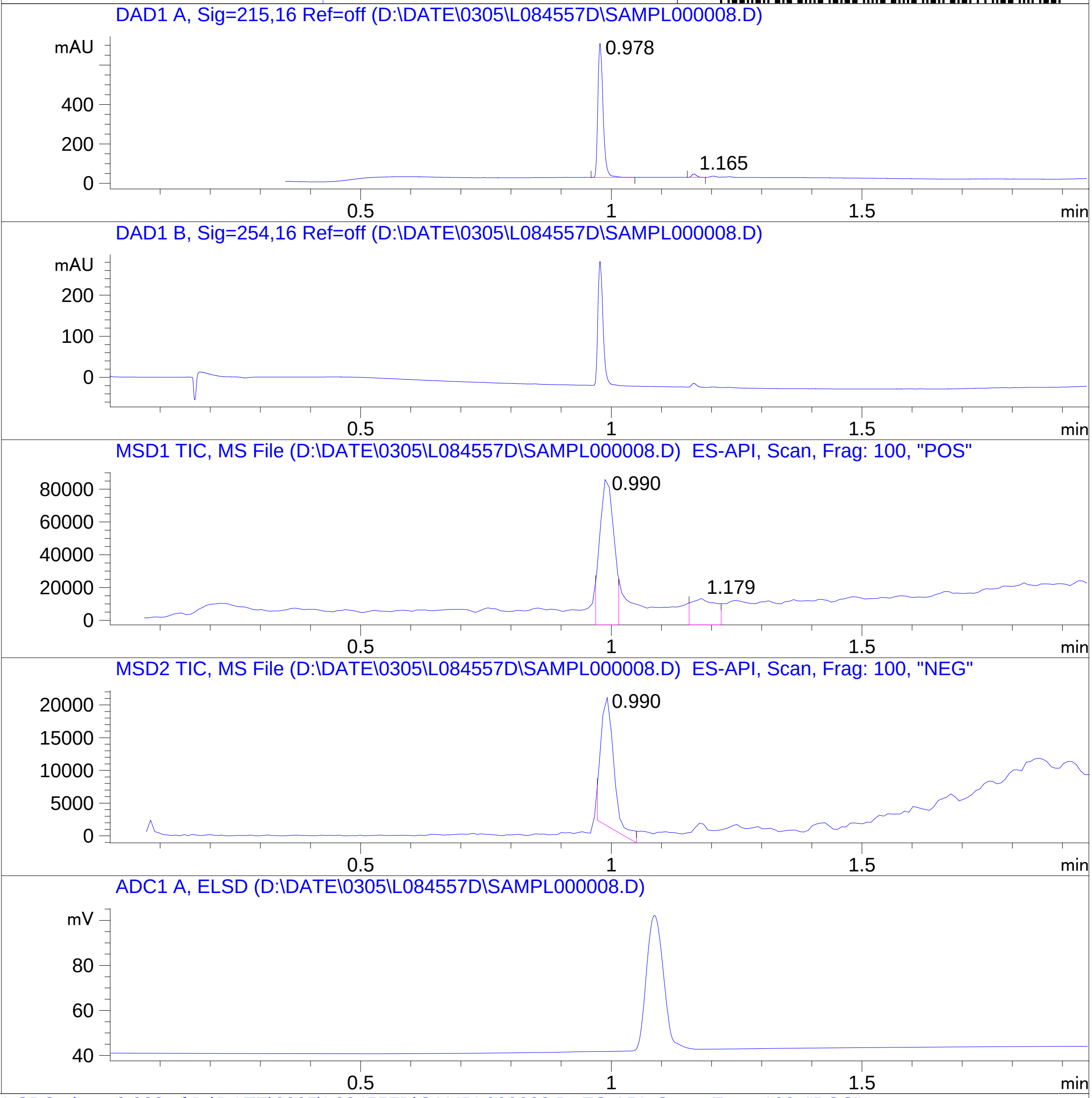

RT 0.990

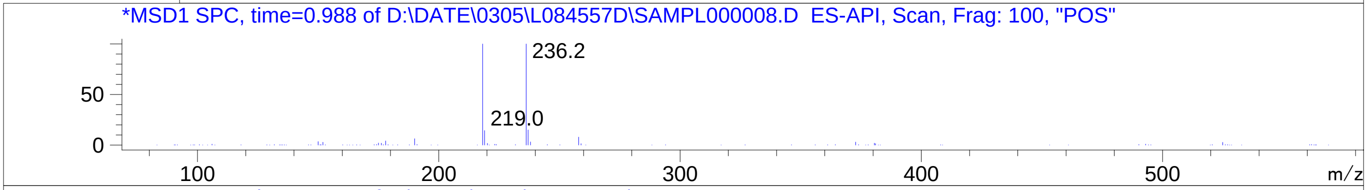

RT 1.179

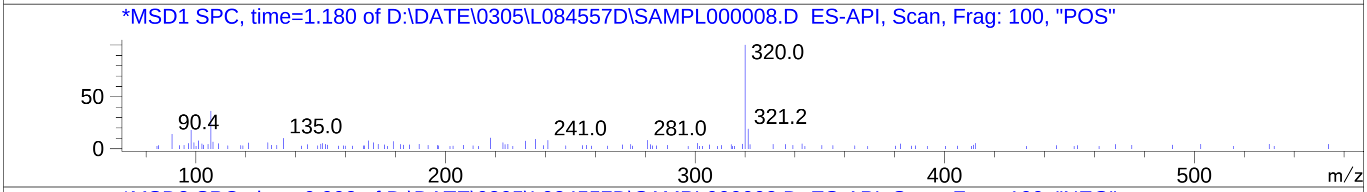

RT 0.990

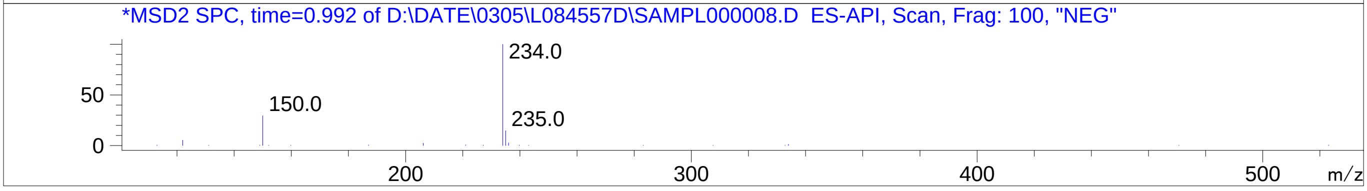

Supplement: Supplementary file 2. [file elife-53779-supp2.zip › mt_vls_62_compounds_QC_data/Compound_18_Z2228267762/Z2228267762_21507825.PDF]
