# Supplementary material for: Structure-based discovery of potent and selective melatonin receptor agonists
Source: eLife. 2020 Mar 2;9:e53779. doi: 10.7554/eLife.53779 (PMC7080406; doi:10.7554/eLife.53779)
Supplement: Supplementary file 2. [file elife-53779-supp2.zip › mt_vls_62_compounds_QC_data/Compound_19_Z1955032980/Z1955032980_21482013.PDF]

MaxPeak: 98.26%
Ret_Time: 0.877 min

L693615$1

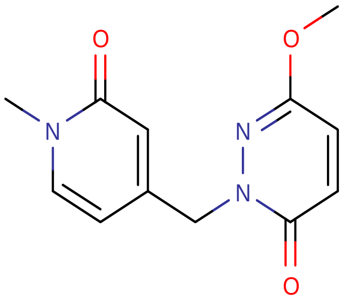

Mol Wt 247.25

Exact Mass 247.1

#	Time	Area%
1	0.859	1.74
2	0.877	98.26

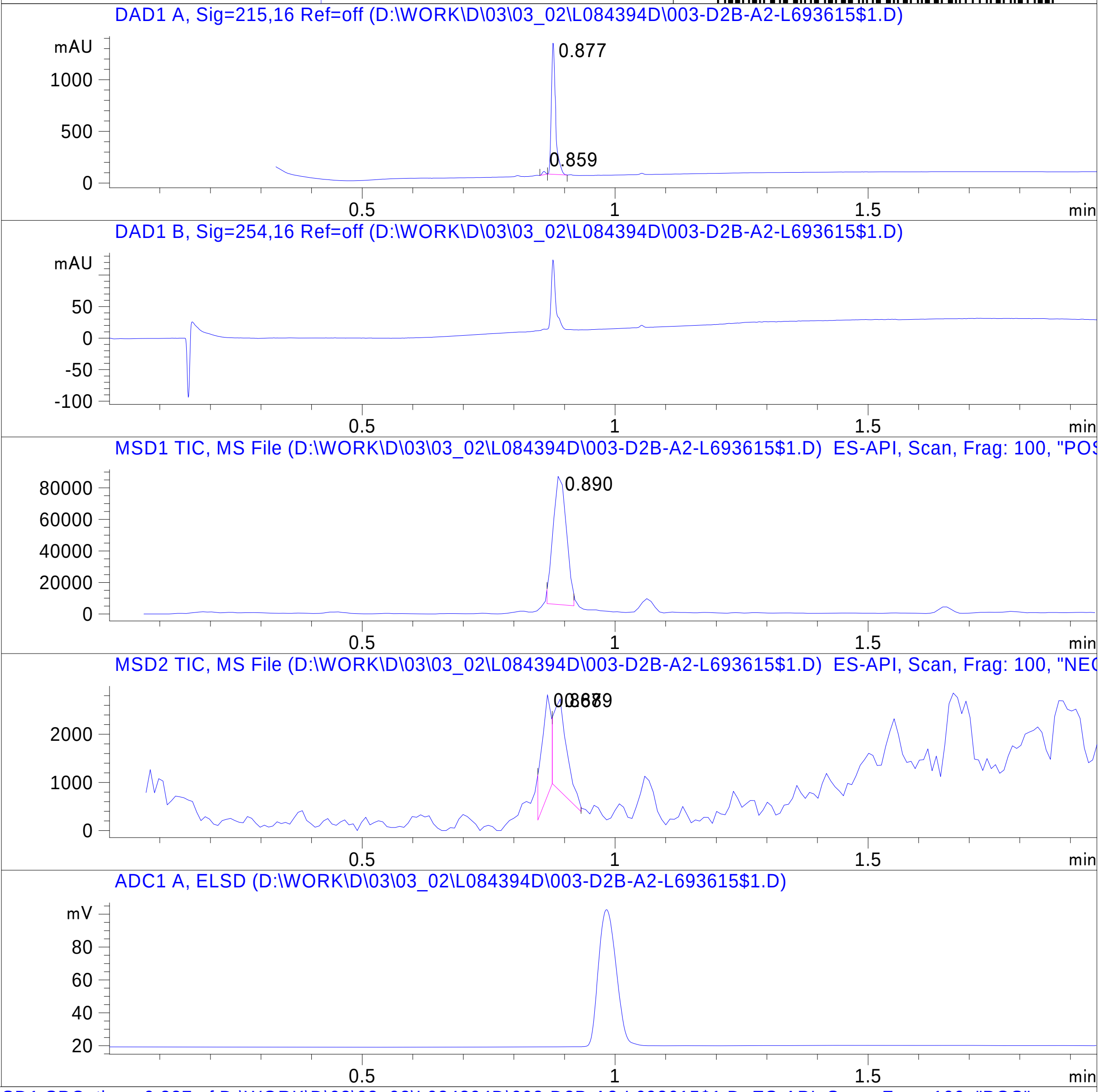

RT 0.890

RT 0.867

RT 0.889

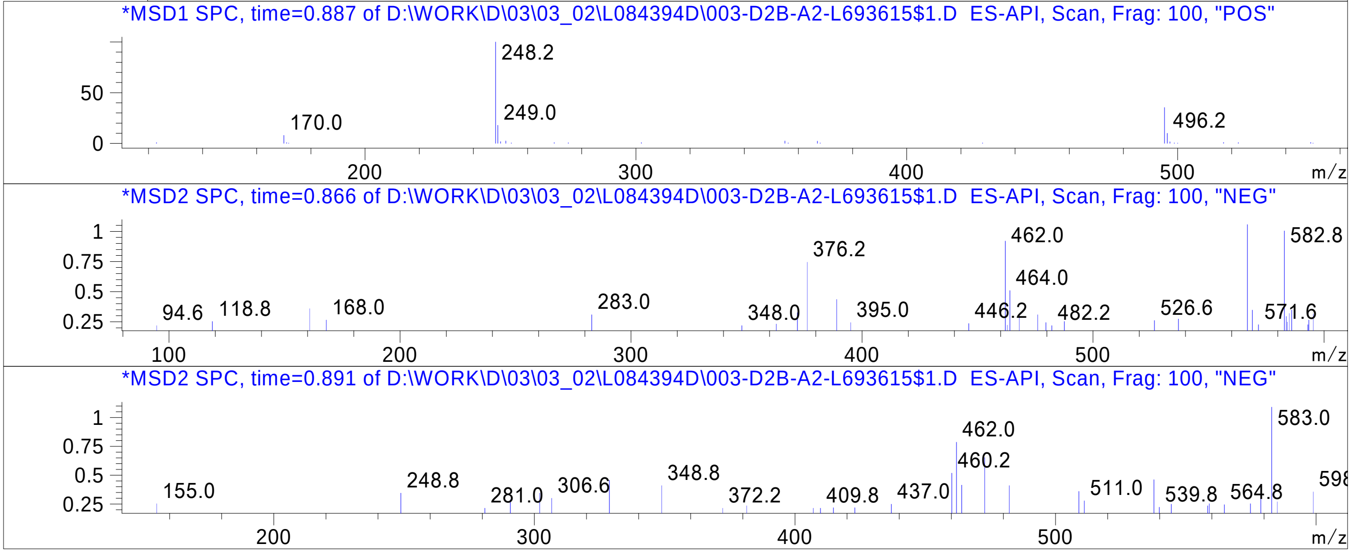

Inj.Date 3/2/2018 K <invalid> -6- Acq. Method C:\Chem32\-> ->
