# Supplementary material for: Structure-based discovery of potent and selective melatonin receptor agonists
Source: eLife. 2020 Mar 2;9:e53779. doi: 10.7554/eLife.53779 (PMC7080406; doi:10.7554/eLife.53779)

MaxPeak: 100.00%
Ret_Time: 0.675 min

L693619$1

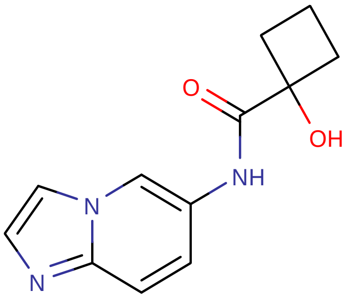

Mol Wt 231.25

Exact Mass 231.11

#	Time	Area%
1	0.675	100.00

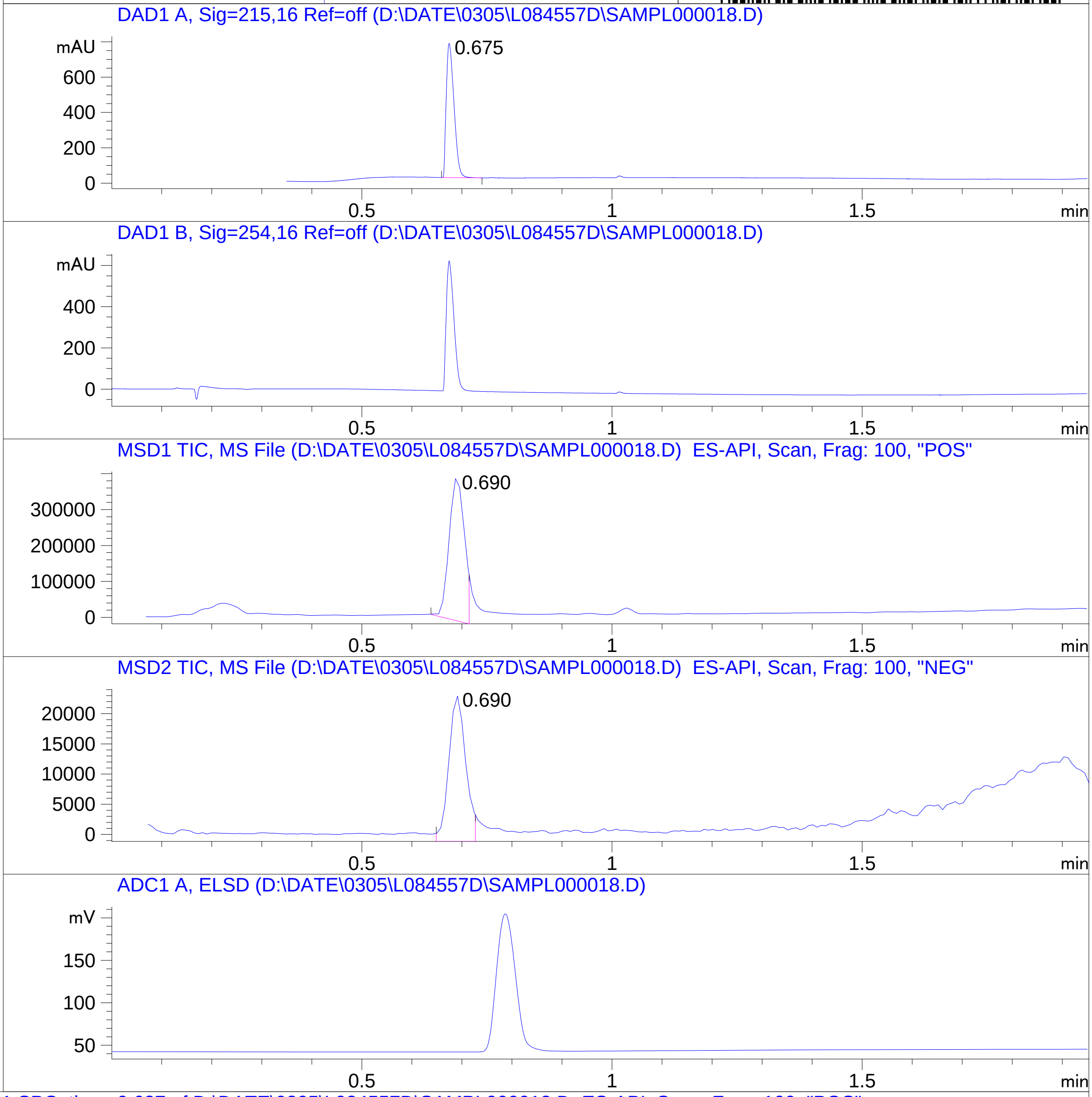

RT 0.690

RT 0.690

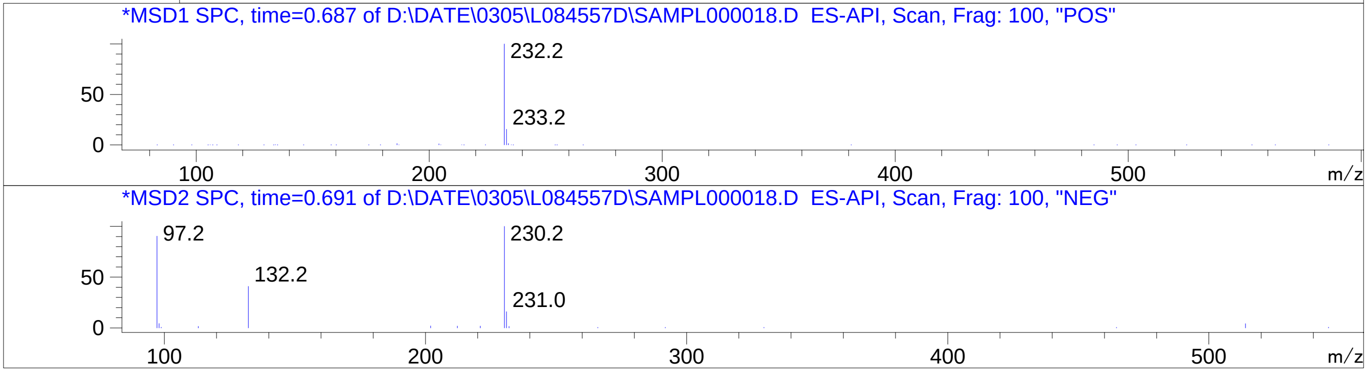

Supplement: Supplementary file 2. [file elife-53779-supp2.zip › mt_vls_62_compounds_QC_data/Compound_2_Z1725992474/Z1725992474_21507835.PDF]
