# Supplementary material for: Structure-based discovery of potent and selective melatonin receptor agonists
Source: eLife. 2020 Mar 2;9:e53779. doi: 10.7554/eLife.53779 (PMC7080406; doi:10.7554/eLife.53779)
Supplement: Supplementary file 2. [file elife-53779-supp2.zip › mt_vls_62_compounds_QC_data/Compound_22_Z2799987596/Z2799987596_21507698.PDF]

MaxPeak: 94.43%
Ret_Time: 1.059 min

L693621$2

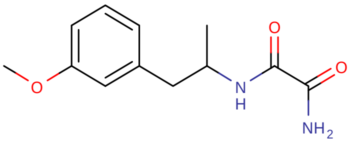

Mol Wt 236.27

Exact Mass 236.13

#	Time	Area%
1	1.059	94.43
2	1.102	5.57

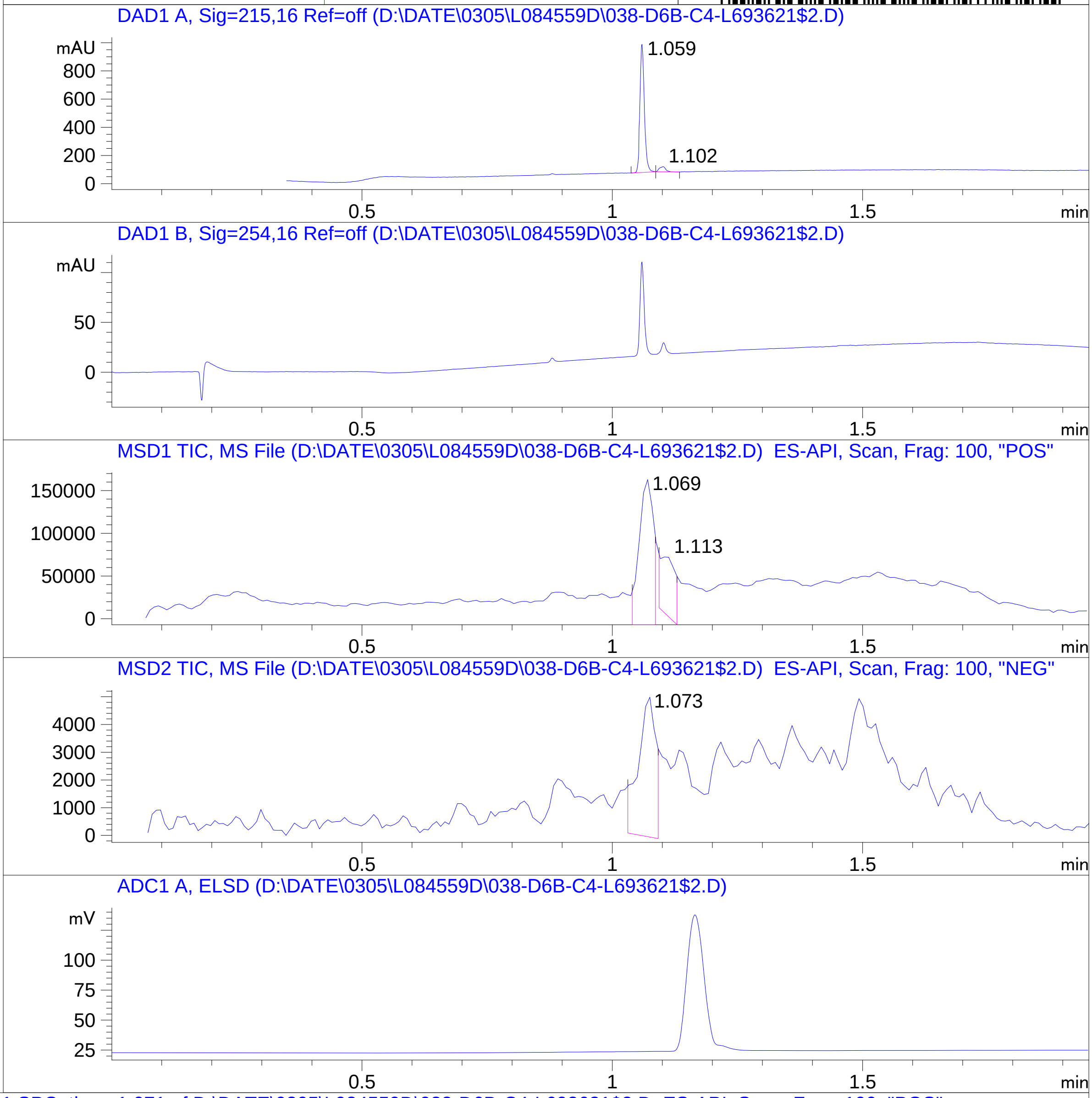

RT 1.069

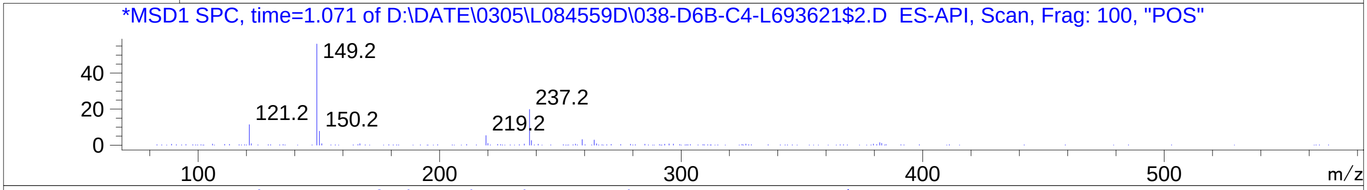

RT 1.113

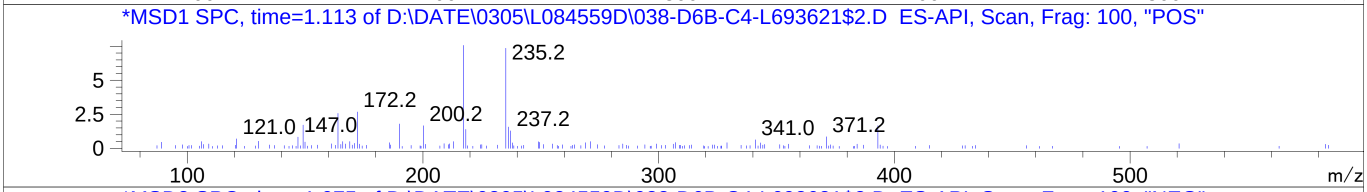

RT 1.073

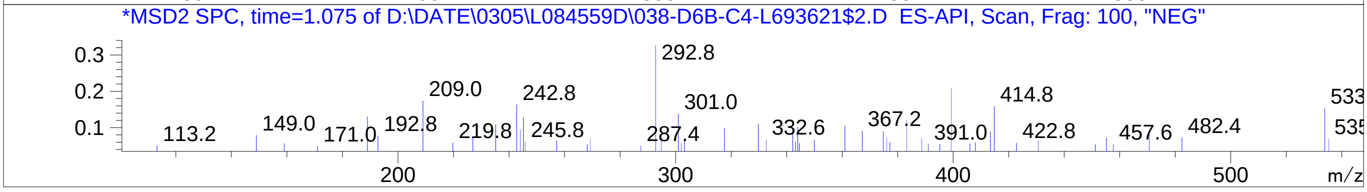

Inj.Date 3/5/2018 E <invalid> -> Acq. Method C:\Chem32\-> ->
