# Supplementary material for: Structure-based discovery of potent and selective melatonin receptor agonists
Source: eLife. 2020 Mar 2;9:e53779. doi: 10.7554/eLife.53779 (PMC7080406; doi:10.7554/eLife.53779)

```
MaxPeak:  100.00%
Ret_Time: 1.131 min
```

L693630$9

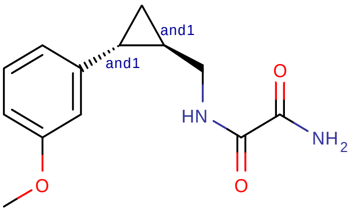

Mol Wt 248.28

Exact Mass 248.13

#	Time	Area%
1	1.131	100.00

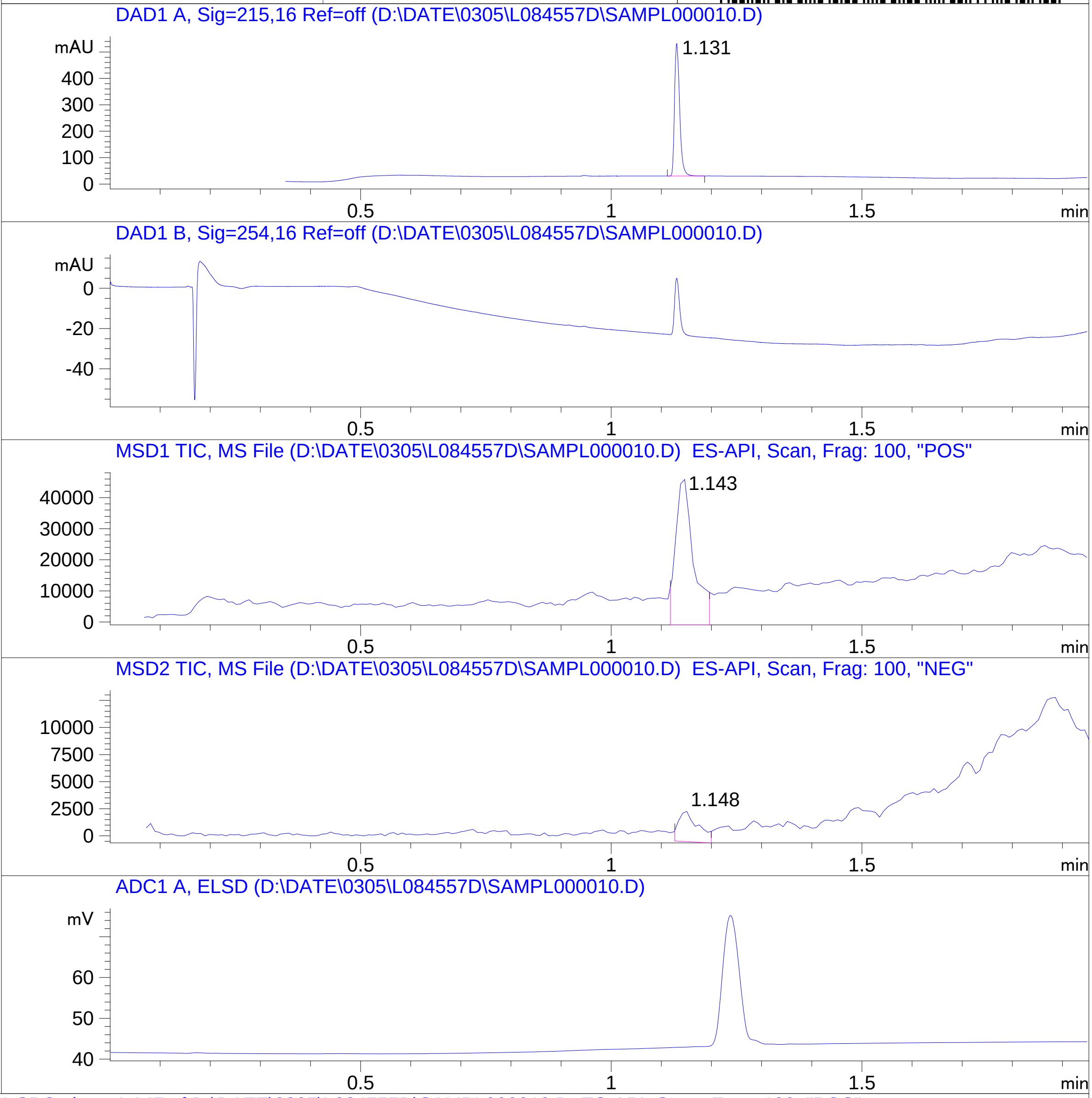

RT 1.143

RT 1.148

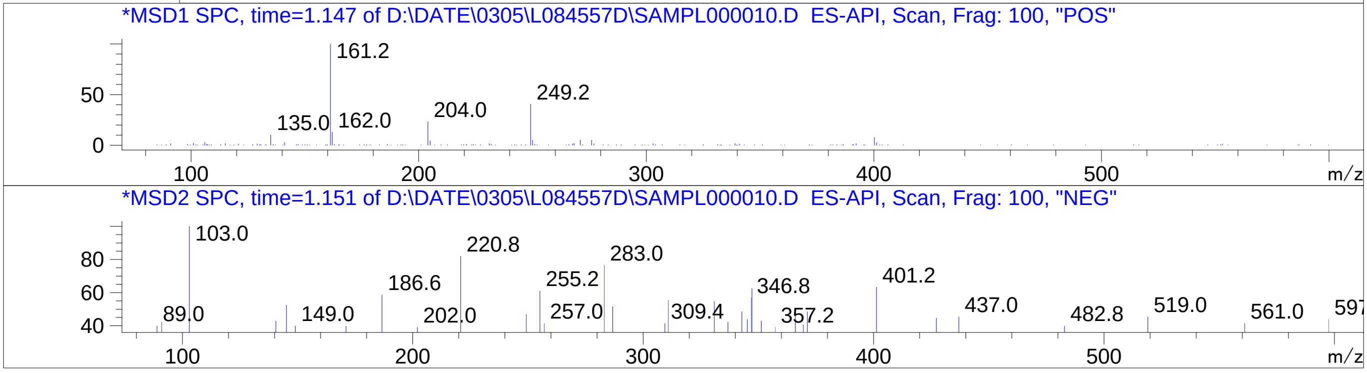

Supplement: Supplementary file 2. [file elife-53779-supp2.zip › mt_vls_62_compounds_QC_data/Compound_23_Z2801662476/Z2801662476_21507827.PDF]
