# Supplementary material for: Structure-based discovery of potent and selective melatonin receptor agonists
Source: eLife. 2020 Mar 2;9:e53779. doi: 10.7554/eLife.53779 (PMC7080406; doi:10.7554/eLife.53779)

MaxPeak: 95.31%
Ret_Time: 0.641 min

L693627$34

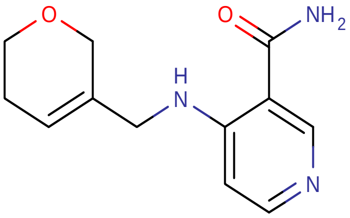

Mol Wt 233.27

Exact Mass 233.13

#	Time	Area%
1	0.641	95.31
2	0.965	1.22
3	1.074	3.47

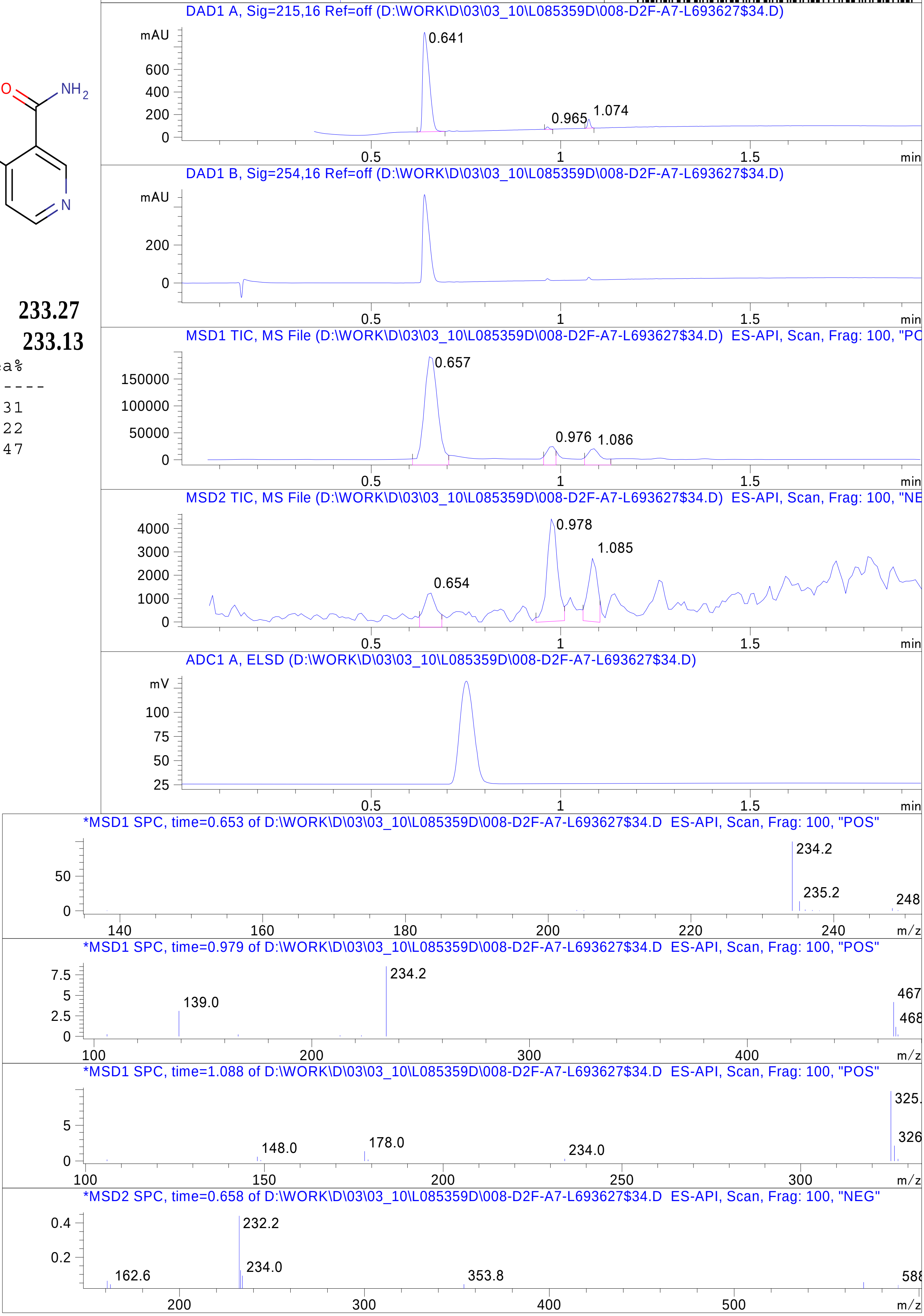

RT 0.657

RT 0.976

RT 1.086

RT 0.654

Inj.Date 3/9/2018 K <invalid> -6- Acq. Method C:\Chem32\-> ->

RT 0.978

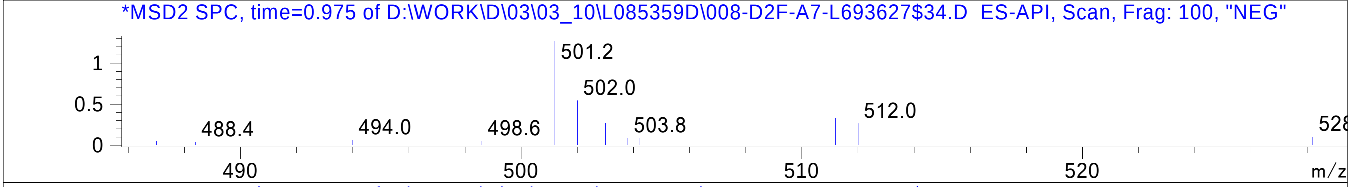

RT 1.085

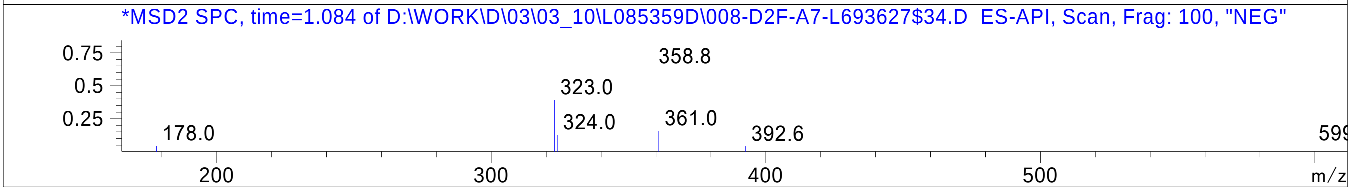

Supplement: Supplementary file 2. [file elife-53779-supp2.zip › mt_vls_62_compounds_QC_data/Compound_24_Z2143496175/Z2143496175_21549267.PDF]
