# Supplementary material for: Structure-based discovery of potent and selective melatonin receptor agonists
Source: eLife. 2020 Mar 2;9:e53779. doi: 10.7554/eLife.53779 (PMC7080406; doi:10.7554/eLife.53779)

MaxPeak: 97.51%
Ret_Time: 0.700 min

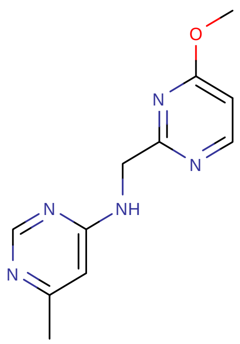

Mol Wt 231.25
Exact Mass 231.12

#	Time	Area%
1	0.700	97.51
2	0.986	2.49

L693649$4

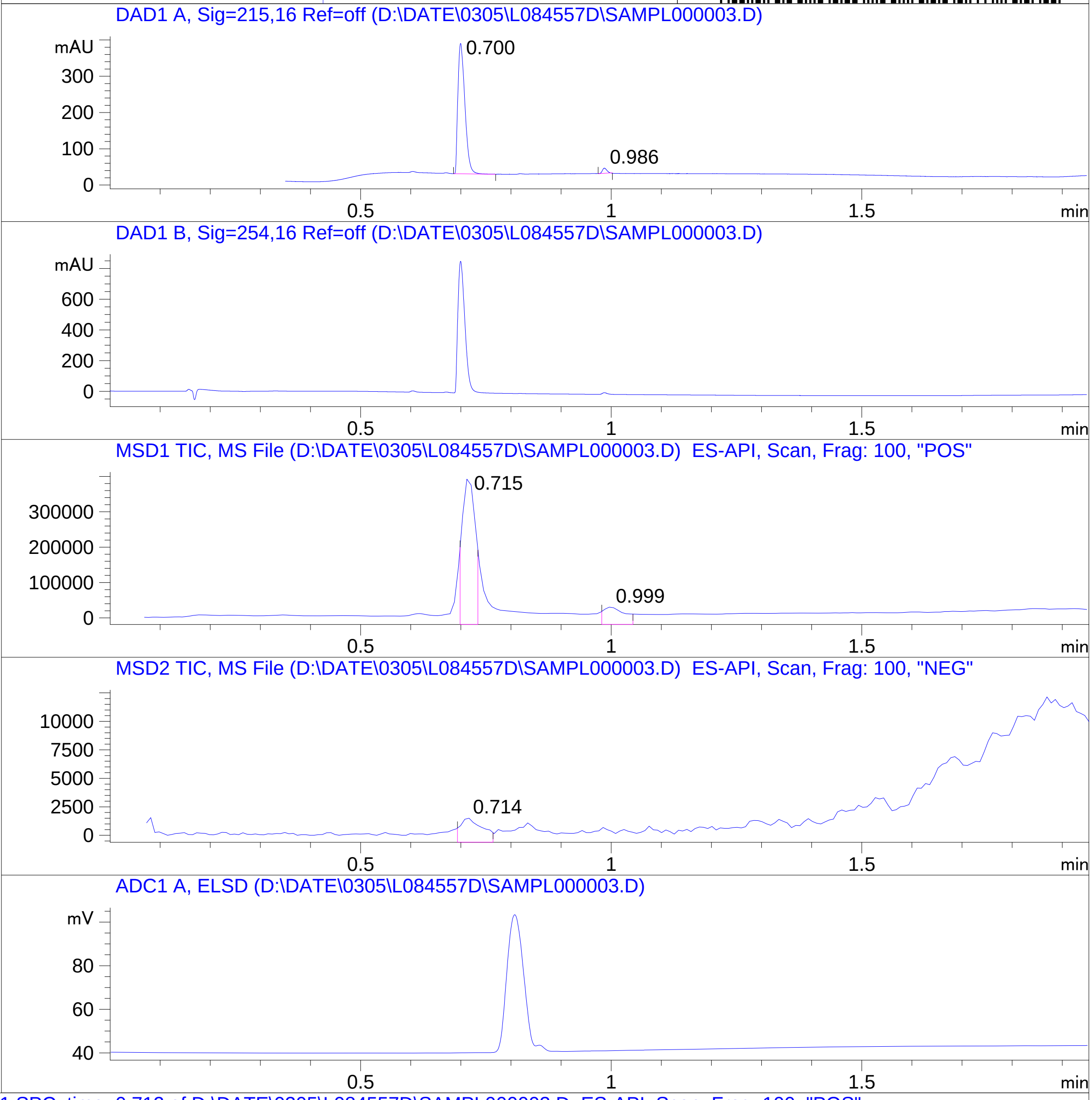

RT 0.715

RT 0.999

RT 0.714

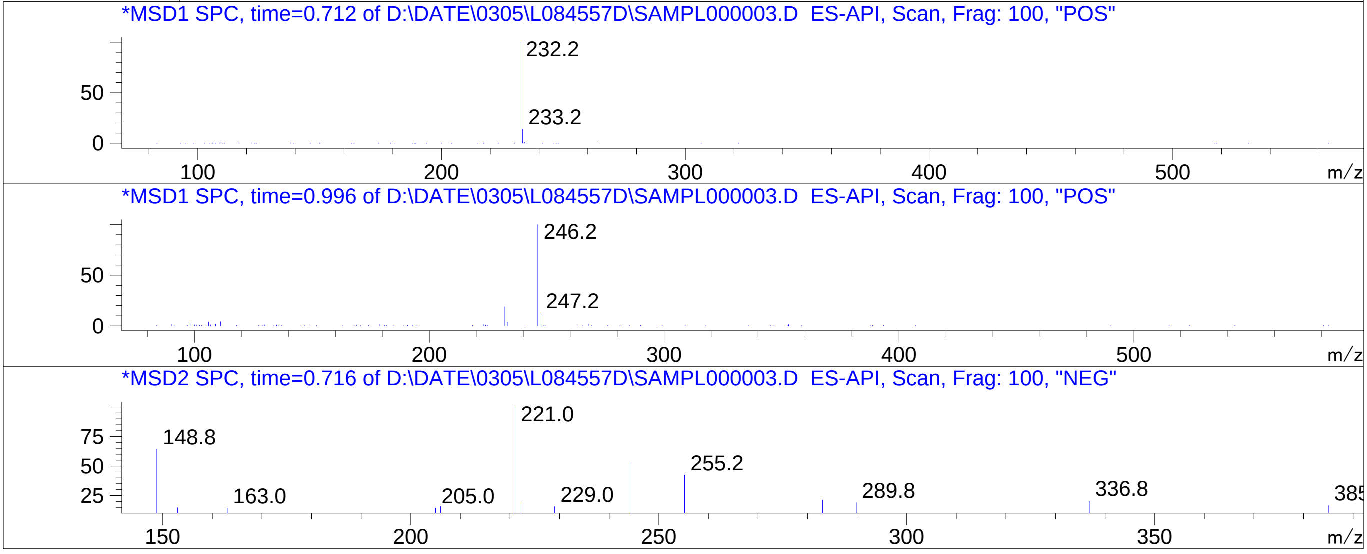

Supplement: Supplementary file 2. [file elife-53779-supp2.zip › mt_vls_62_compounds_QC_data/Compound_25_Z2409717456/Z2409717456_21507820.PDF]
