# Supplementary material for: Structure-based discovery of potent and selective melatonin receptor agonists
Source: eLife. 2020 Mar 2;9:e53779. doi: 10.7554/eLife.53779 (PMC7080406; doi:10.7554/eLife.53779)

MaxPeak: 98.89%
Ret_Time: 0.951 min

L693635$5

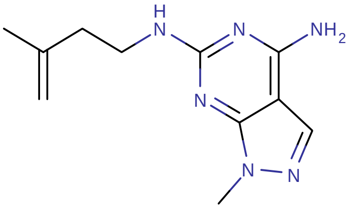

Mol Wt 232.28

Exact Mass 232.16

#	Time	Area%
1	0.951	98.89
2	1.116	1.11

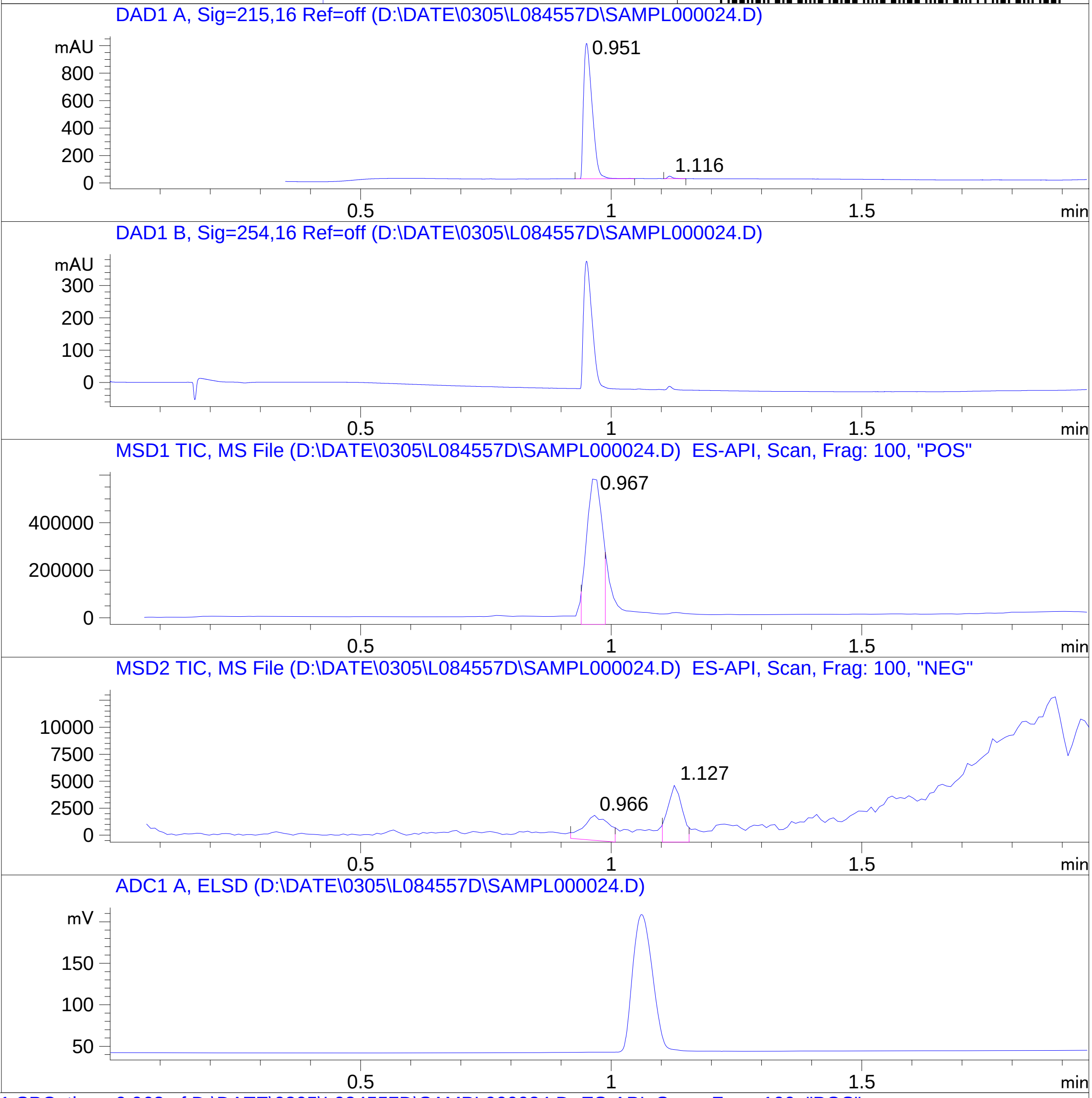

RT 0.967

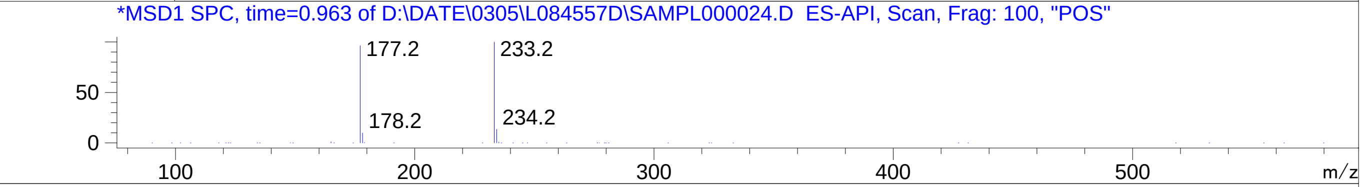

RT 0.966

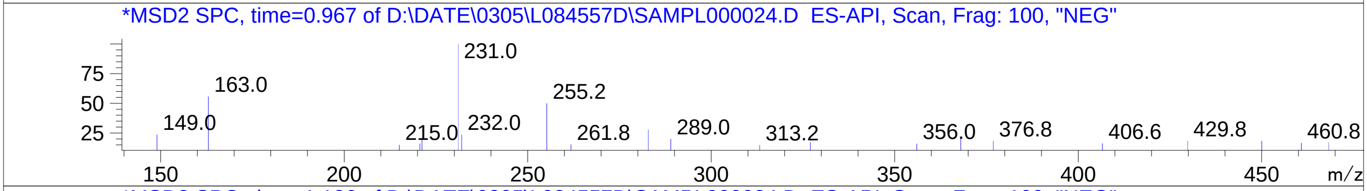

RT 1.127

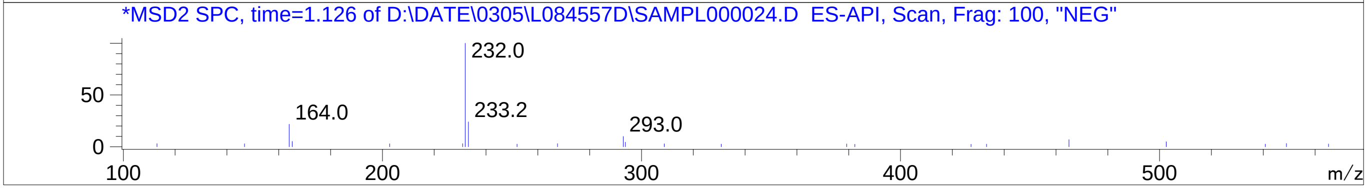

Supplement: Supplementary file 2. [file elife-53779-supp2.zip › mt_vls_62_compounds_QC_data/Compound_27_Z2273688472/Z2273688472_21507841.PDF]
