# Supplementary material for: Structure-based discovery of potent and selective melatonin receptor agonists
Source: eLife. 2020 Mar 2;9:e53779. doi: 10.7554/eLife.53779 (PMC7080406; doi:10.7554/eLife.53779)

L693625$1

MaxPeak: 100.00%
Ret_Time: 0.903 min

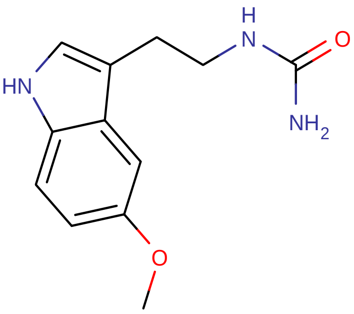

Mol Wt 233.27

Exact Mass 233.13

#	Time	Area%
1	0.903	100.00

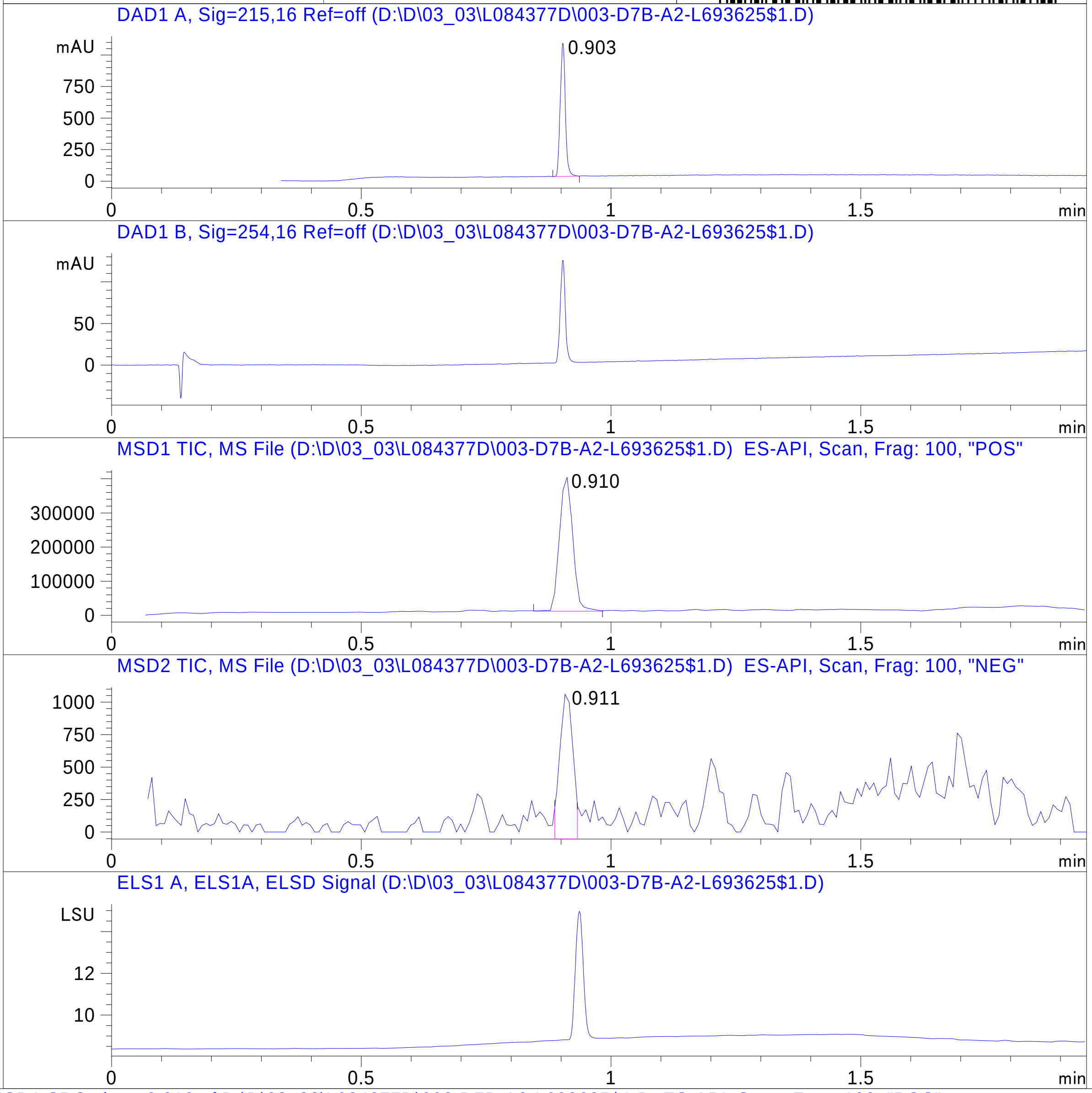

RT 0.910

RT 0.911

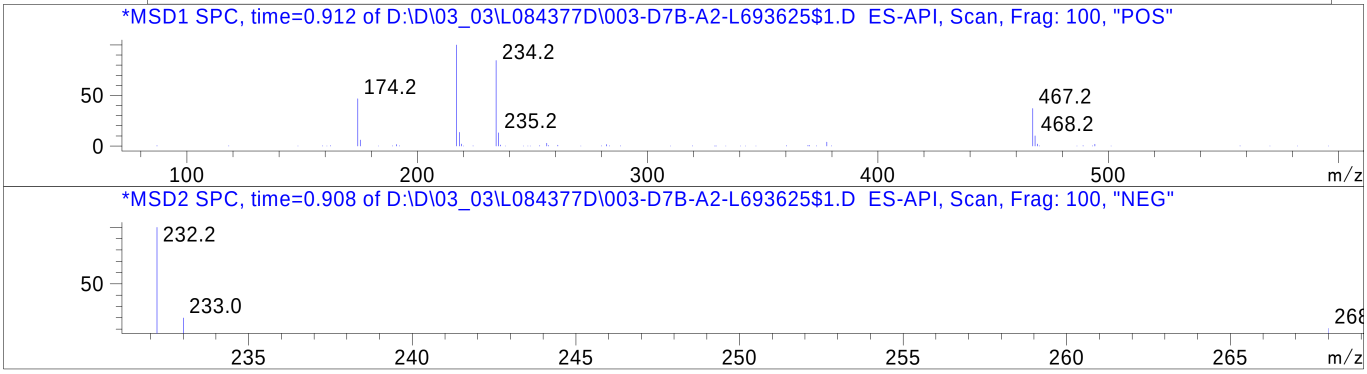

Supplement: Supplementary file 2. [file elife-53779-supp2.zip › mt_vls_62_compounds_QC_data/Compound_28_Z1196462057/Z1196462057_21481718.PDF]
