# Supplementary material for: Structure-based discovery of potent and selective melatonin receptor agonists
Source: eLife. 2020 Mar 2;9:e53779. doi: 10.7554/eLife.53779 (PMC7080406; doi:10.7554/eLife.53779)
Supplement: Supplementary file 2. [file elife-53779-supp2.zip › mt_vls_62_compounds_QC_data/Compound_29_Z2701721005/Z2701721005_21482017.PDF]

MaxPeak: 95.45%
Ret_Time: 0.731 min

L693645$4

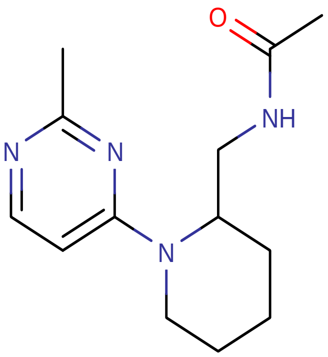

Mol Wt 248.32

Exact Mass 248.19

#	Time	Area%
1	0.731	95.45
2	0.778	1.19
3	0.984	1.49
4	1.341	1.87

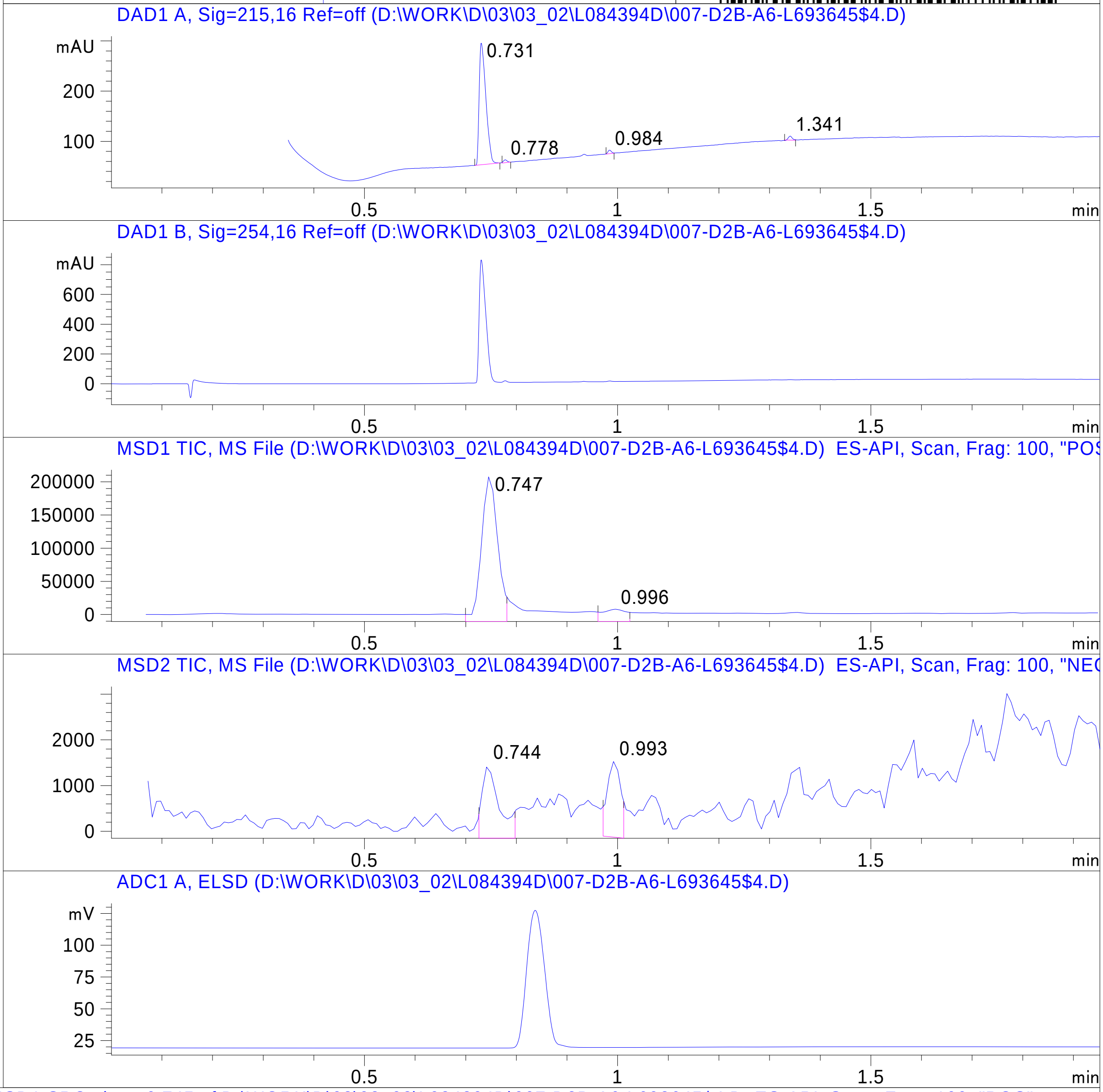

RT 0.747

RT 0.996

RT 0.744

RT 0.993

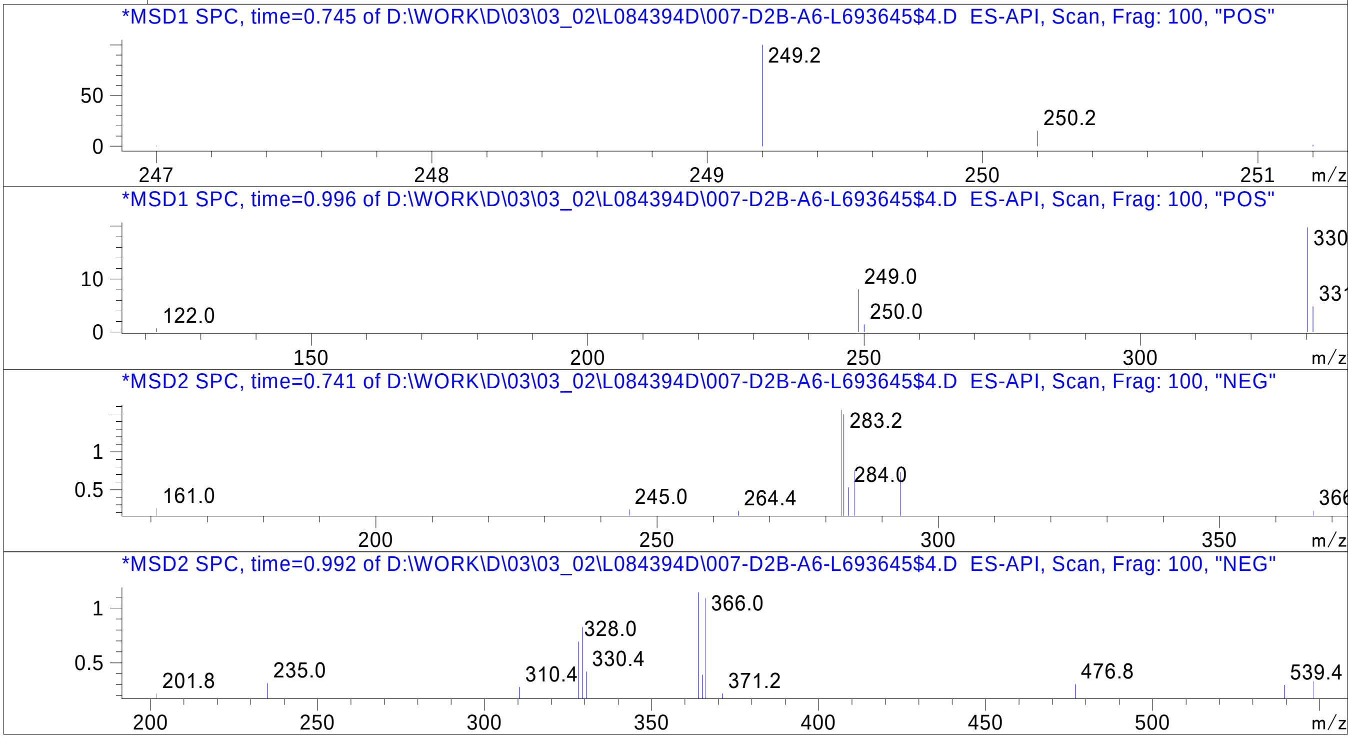

Inj.Date 3/2/2018 K <invalid> -6- Acq. Method C:\Chem32\-> ->
