# Supplementary material for: Structure-based discovery of potent and selective melatonin receptor agonists
Source: eLife. 2020 Mar 2;9:e53779. doi: 10.7554/eLife.53779 (PMC7080406; doi:10.7554/eLife.53779)
Supplement: Supplementary file 2. [file elife-53779-supp2.zip › mt_vls_62_compounds_QC_data/Compound_3_Z1687515356/Z1687515356_21517007.PDF]

MaxPeak: 96.05%
Ret_Time: 0.149 min

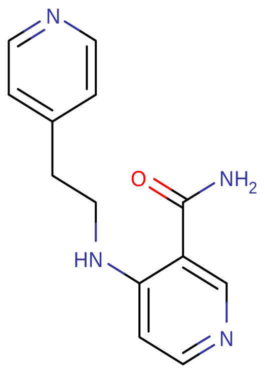

Mol Wt 242.28

Exact Mass 242.13

#	Time	Area%
1	0.149	96.05
2	0.192	3.95

L693618$4

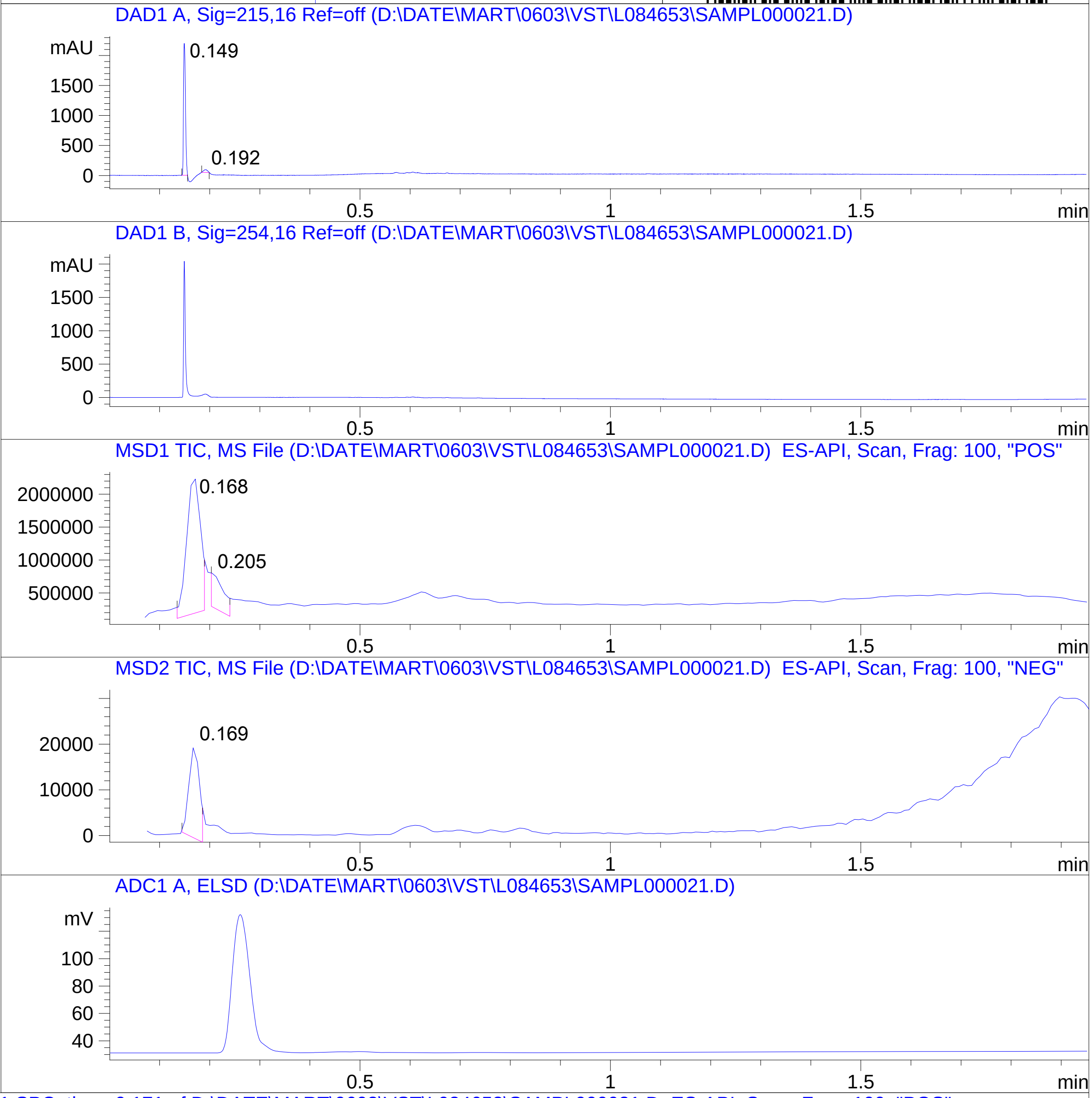

RT 0.168

RT 0.205

RT 0.169

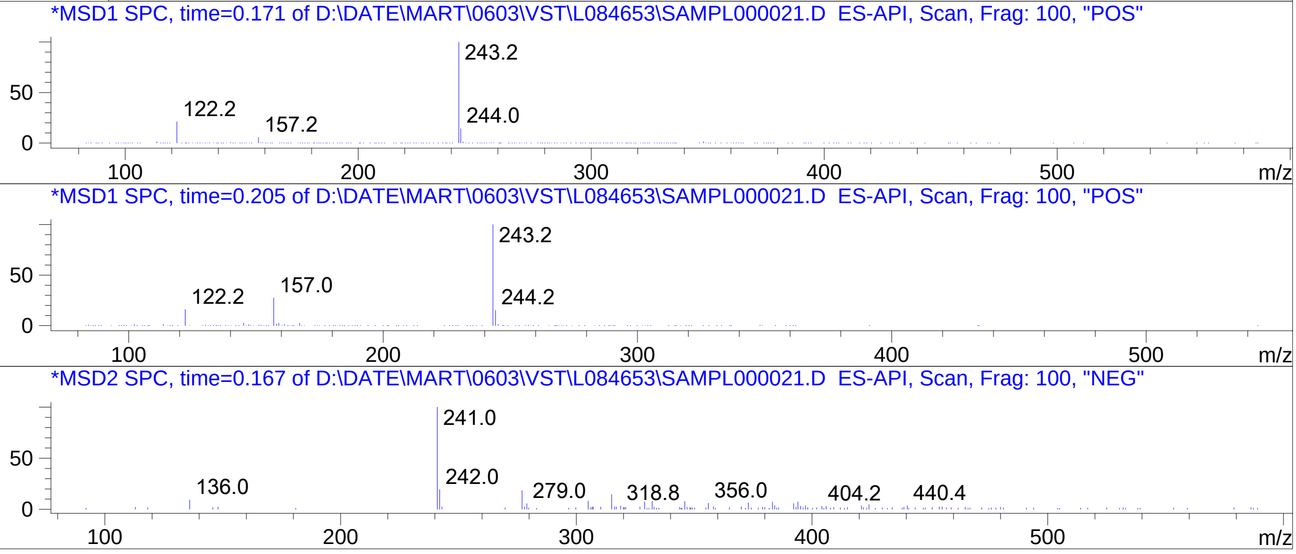

Inj.Date 3/6/2018 Y P1-A-01 -3- Acq. Method C:\CHEM32\-> ->
