# Supplementary material for: Structure-based discovery of potent and selective melatonin receptor agonists
Source: eLife. 2020 Mar 2;9:e53779. doi: 10.7554/eLife.53779 (PMC7080406; doi:10.7554/eLife.53779)
Supplement: Supplementary file 2. [file elife-53779-supp2.zip › mt_vls_62_compounds_QC_data/Compound_31_Z1337812751/Z1337812751_21518094.PDF]

MaxPeak: 100.00%
Ret_Time: 0.987 min

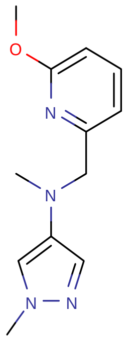

Mol Wt 232.28

Exact Mass 232.15

#	Time	Area%
1	0.987	100.00

L693648$2

DAD1 A, Sig=215,16 Ref=off (D:\DATE\0306\L084754D\SAMPL000012.D)

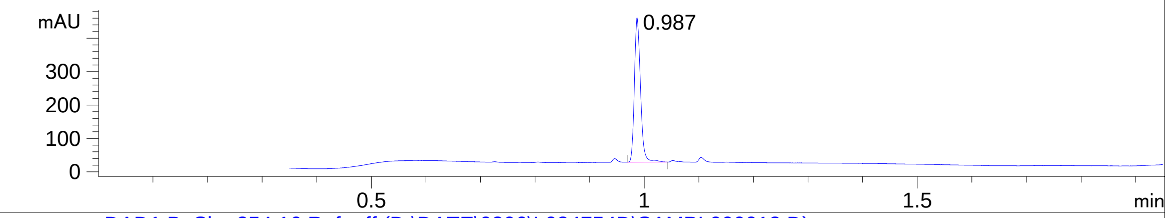

DAD1 B, Sig=254,16 Ref=off (D:\DATE\0306\L084754D\SAMPL000012.D)

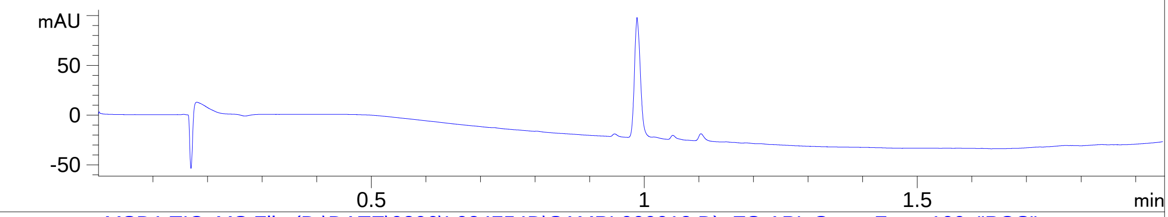

MSD1 TIC, MS File (D:\DATE\0306\L084754D\SAMPL000012.D) ES-API, Scan, Frag: 100, "POS"

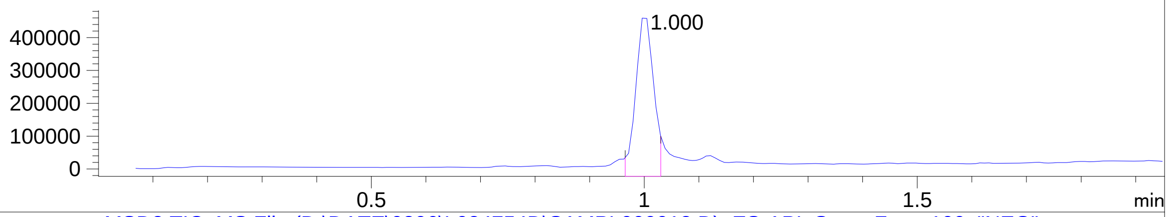

MSD2 TIC, MS File (D:\DATE\0306\L084754D\SAMPL000012.D) ES-API, Scan, Frag: 100, "NEG"

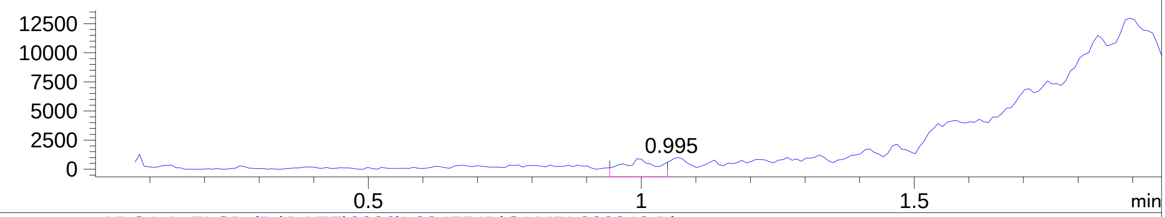

ADC1 A, ELSD (D:\DATE\0306\L084754D\SAMPL000012.D)

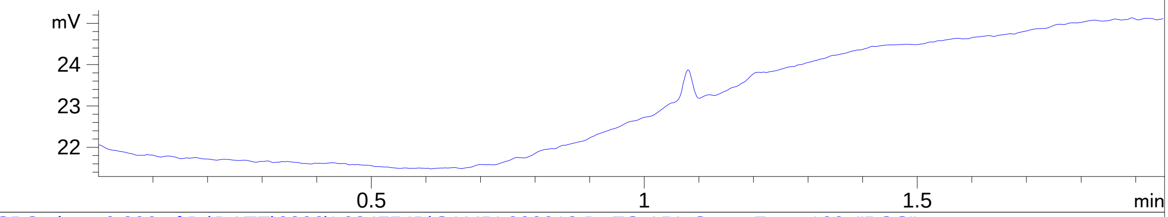

RT 1.000

*MSD1 SPC, time=0.996 of D:\DATE\0306\L084754D\SAMPL000012.D ES-API, Scan, Frag: 100, "POS"

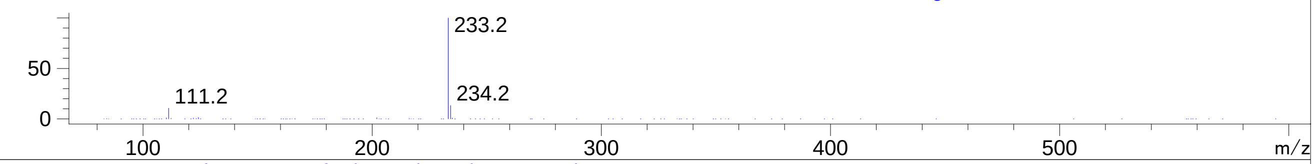

RT 0.995

*MSD2 SPC, time=0.992 of D:\DATE\0306\L084754D\SAMPL000012.D ES-API, Scan, Frag: 100, "NEG"

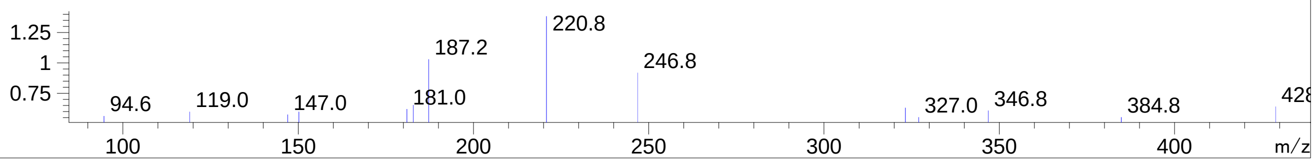

Inj.Date 3/6/2018 E P2-B-03 -5- Acq. Method C:\Chem32\-> ->
