# Supplementary material for: Structure-based discovery of potent and selective melatonin receptor agonists
Source: eLife. 2020 Mar 2;9:e53779. doi: 10.7554/eLife.53779 (PMC7080406; doi:10.7554/eLife.53779)

MaxPeak: 99.28%
Ret_Time: 0.938 min

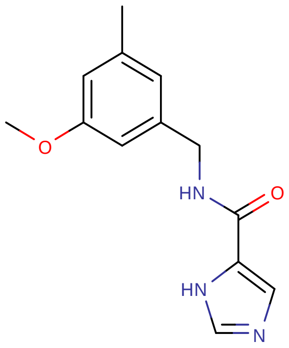

Mol Wt 245.28

Exact Mass 245.13

#	Time	Area%
1	0.840	0.72
2	0.938	99.28

L693612$4

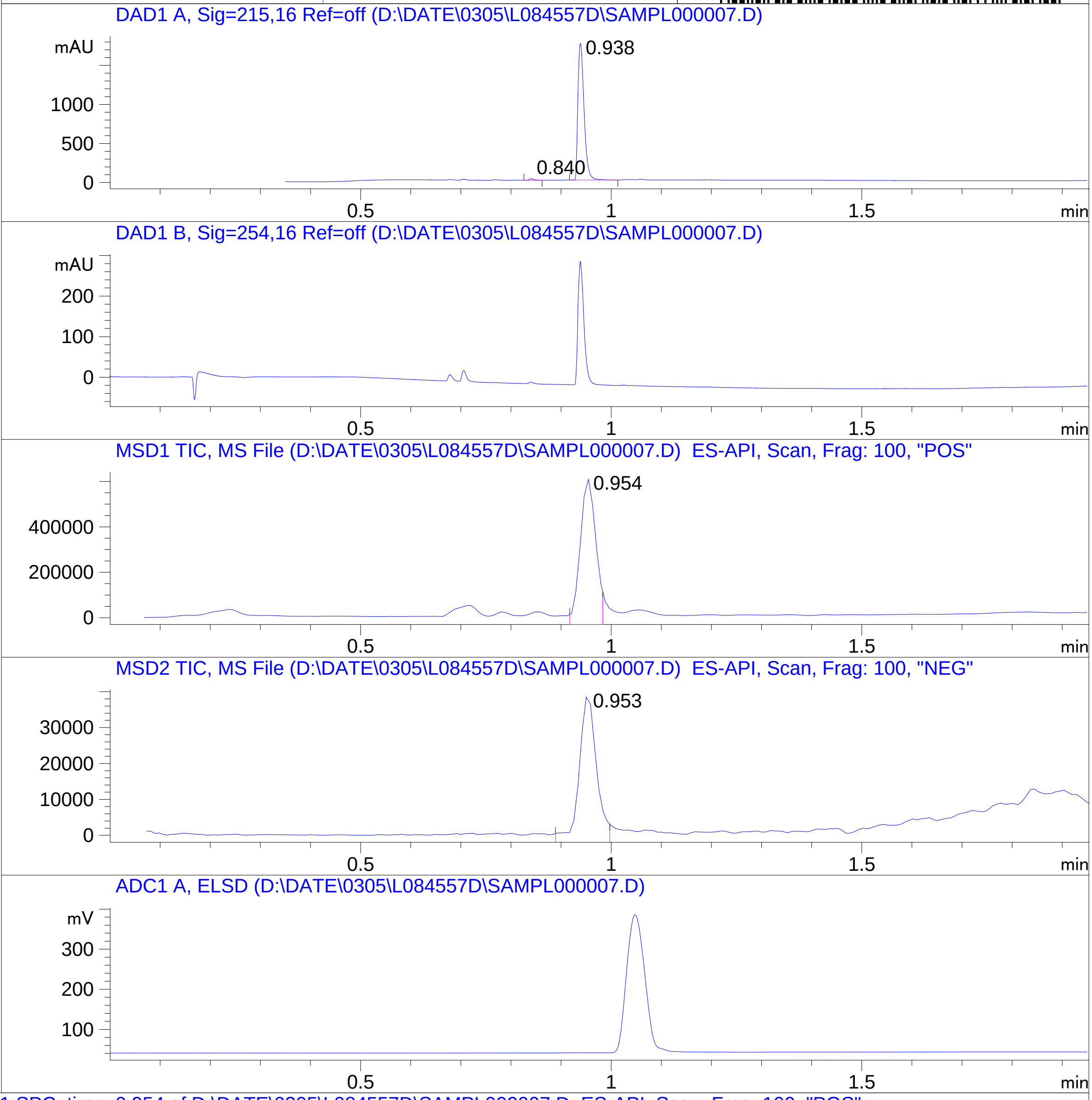

RT 0.954

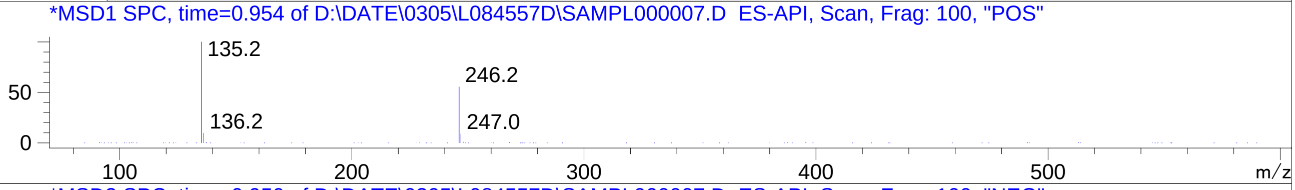

RT 0.953

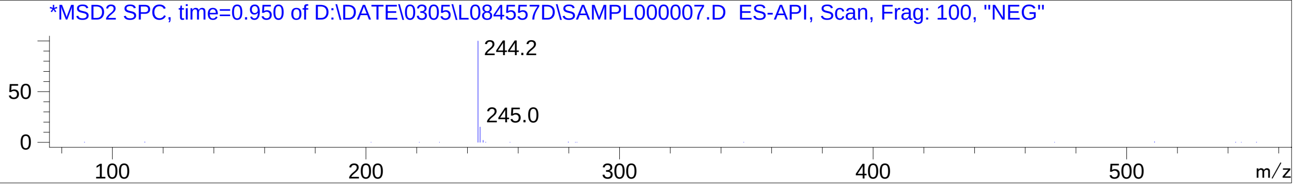

Supplement: Supplementary file 2. [file elife-53779-supp2.zip › mt_vls_62_compounds_QC_data/Compound_32_Z1499411301/Z1499411301_21507824.PDF]
