# Supplementary material for: Structure-based discovery of potent and selective melatonin receptor agonists
Source: eLife. 2020 Mar 2;9:e53779. doi: 10.7554/eLife.53779 (PMC7080406; doi:10.7554/eLife.53779)

MaxPeak: 100.00%
Ret_Time: 1.069 min

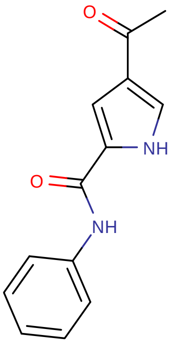

Mol Wt 228.25

Exact Mass 228.1

#	Time	Area%
1	1.069	100.00

L628494$1

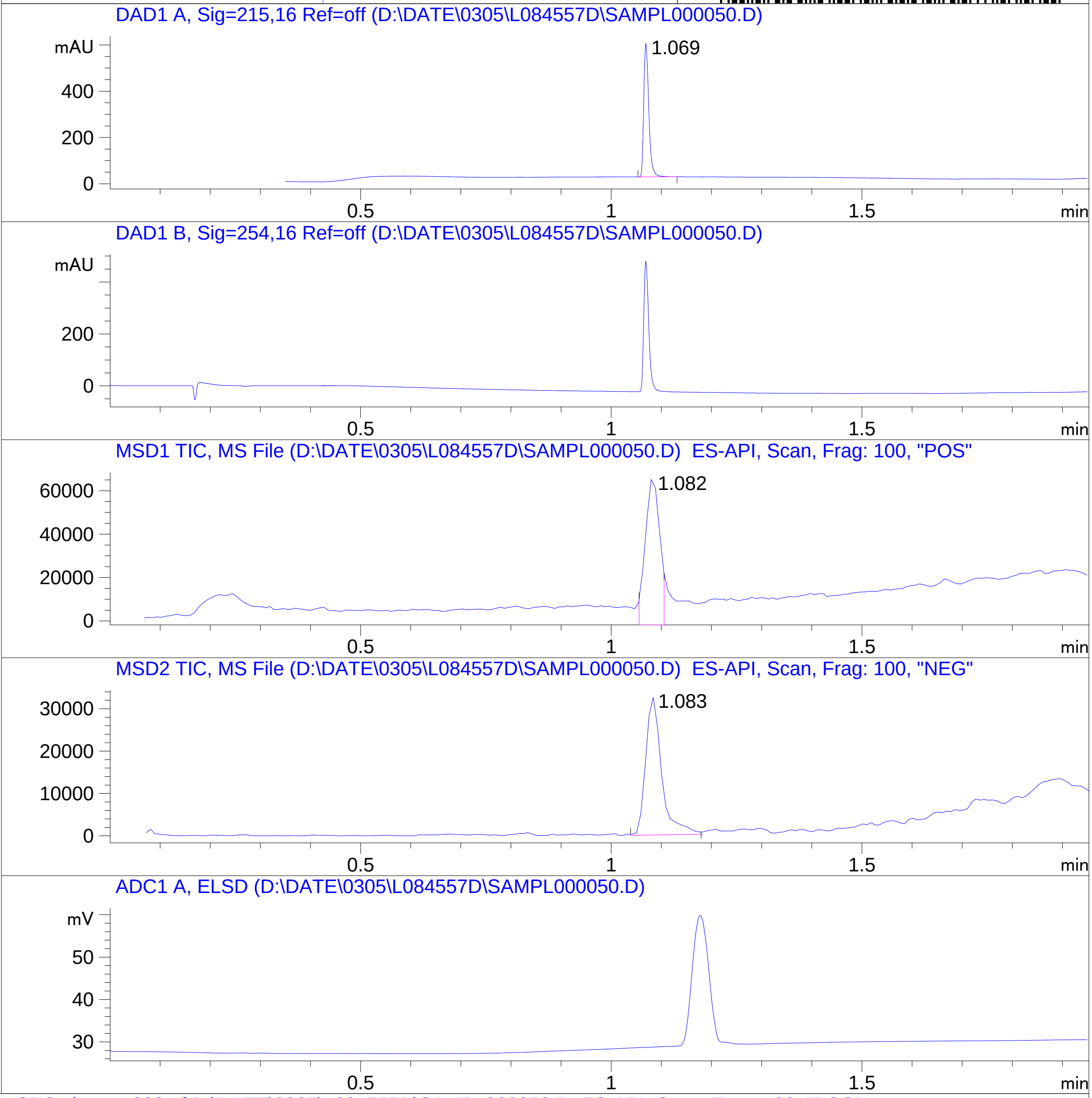

RT 1.082

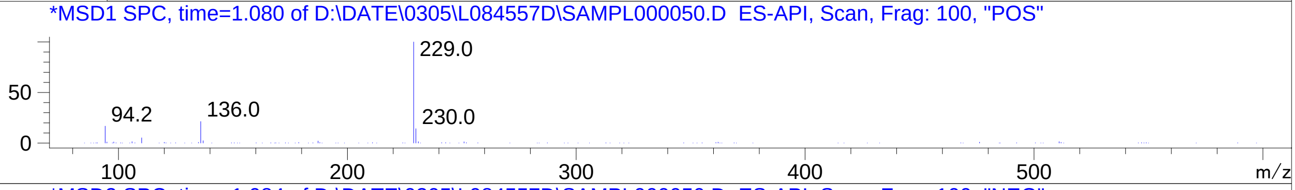

RT 1.083

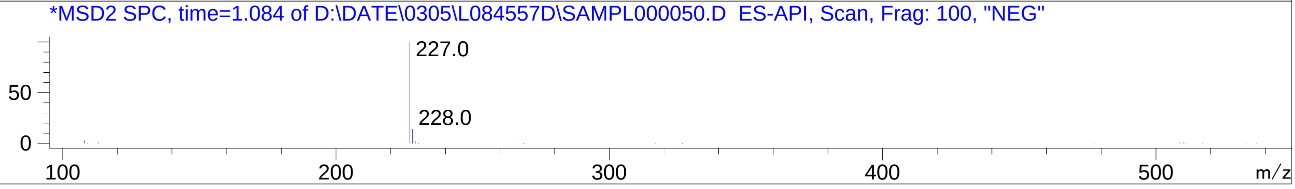

Supplement: Supplementary file 2. [file elife-53779-supp2.zip › mt_vls_62_compounds_QC_data/Compound_34_Z108630396/Z108630396_21507867.PDF]
