# Supplementary material for: Structure-based discovery of potent and selective melatonin receptor agonists
Source: eLife. 2020 Mar 2;9:e53779. doi: 10.7554/eLife.53779 (PMC7080406; doi:10.7554/eLife.53779)
Supplement: Supplementary file 2. [file elife-53779-supp2.zip › mt_vls_62_compounds_QC_data/Compound_35_Z2212371816/Z2212371816_21523448.PDF]

L693641$1

MaxPeak: 100.00%
Ret_Time: 1.178 min

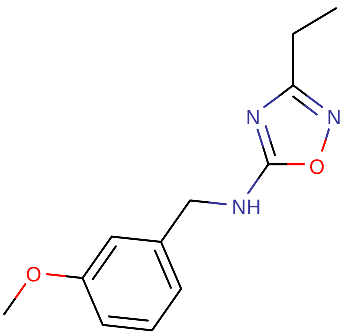

Mol Wt 233.27

Exact Mass 233.13

#	Time	Area%
1	1.178	100.00

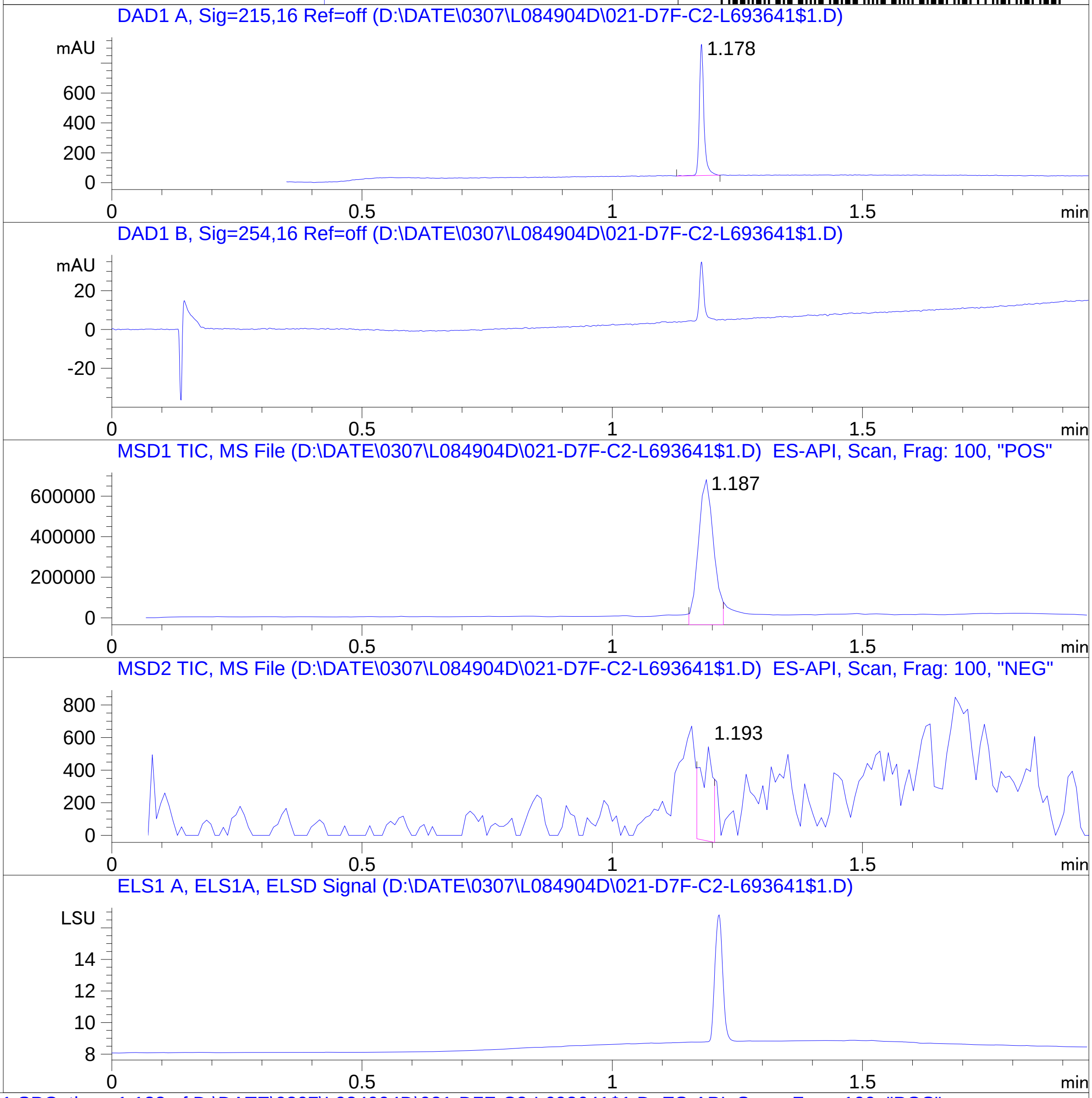

RT 1.187

RT 1.193

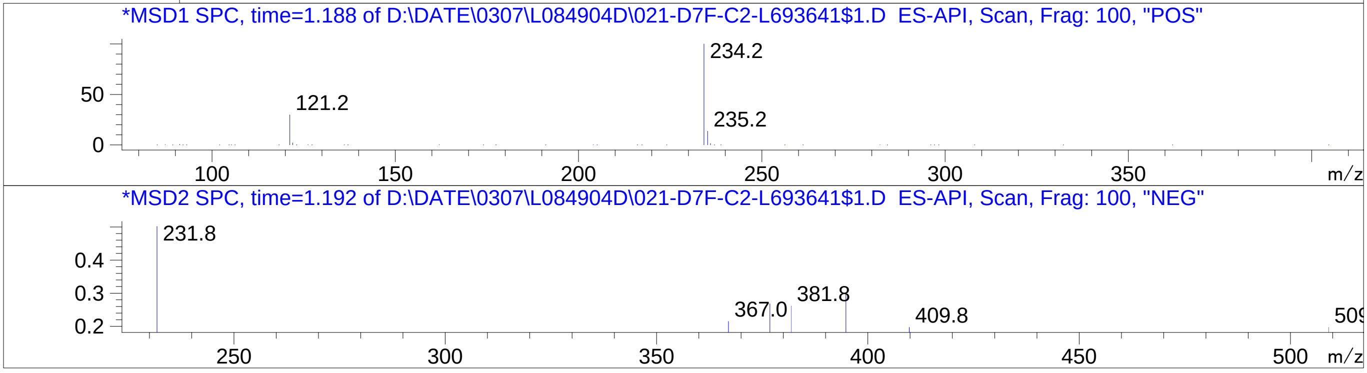

Inj.Date 3/6/2018 E <invalid> -17- Acq. Method C:\Chem32\-> ->
