# Supplementary material for: Structure-based discovery of potent and selective melatonin receptor agonists
Source: eLife. 2020 Mar 2;9:e53779. doi: 10.7554/eLife.53779 (PMC7080406; doi:10.7554/eLife.53779)

MaxPeak: 100.00%
Ret_Time: 0.813 min

L693611$7

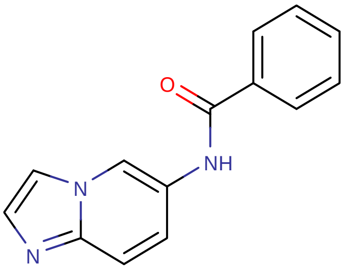

Mol Wt 237.26

Exact Mass 237.1

#	Time	Area%
1	0.813	100.00

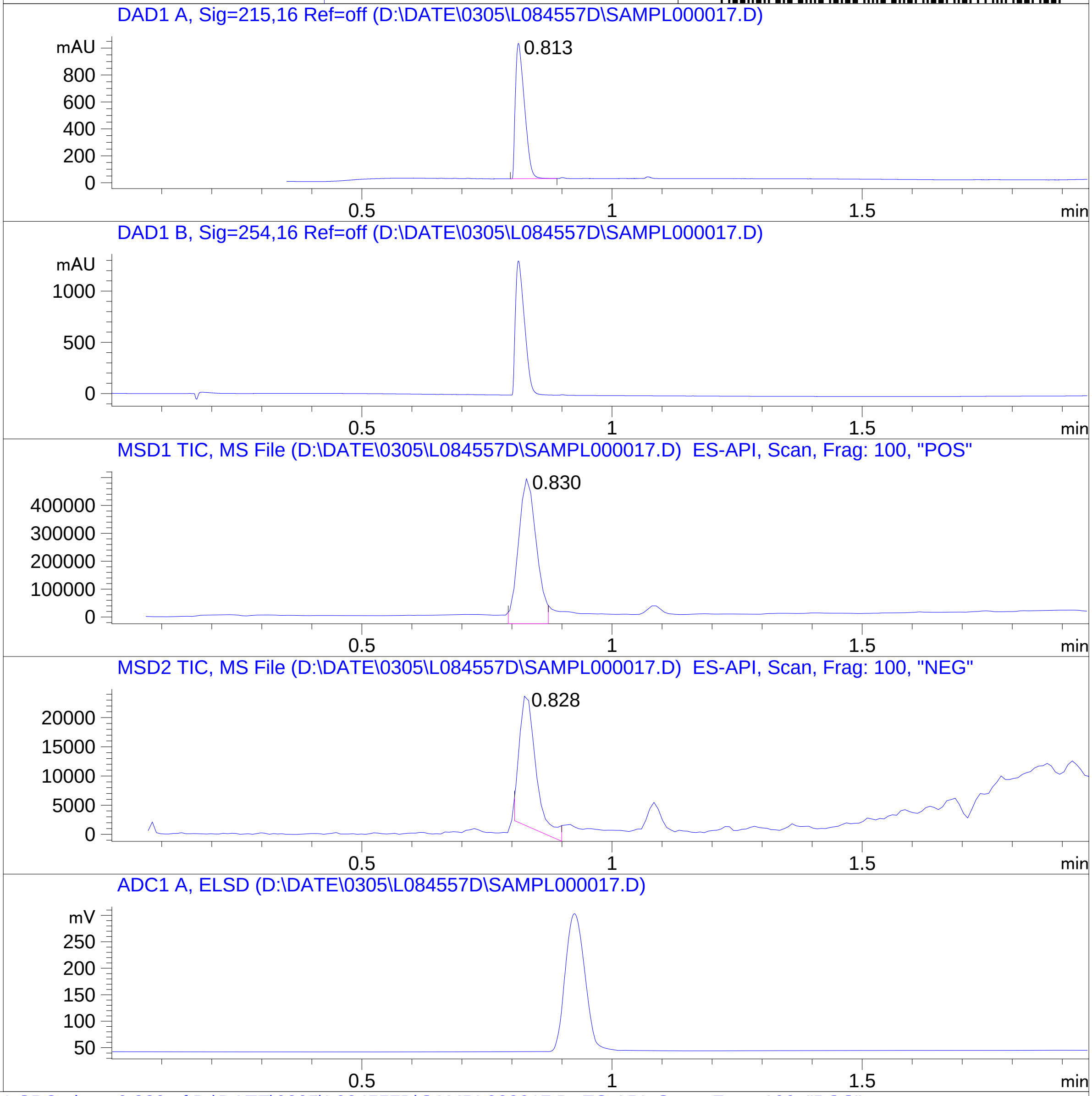

RT 0.830

RT 0.828

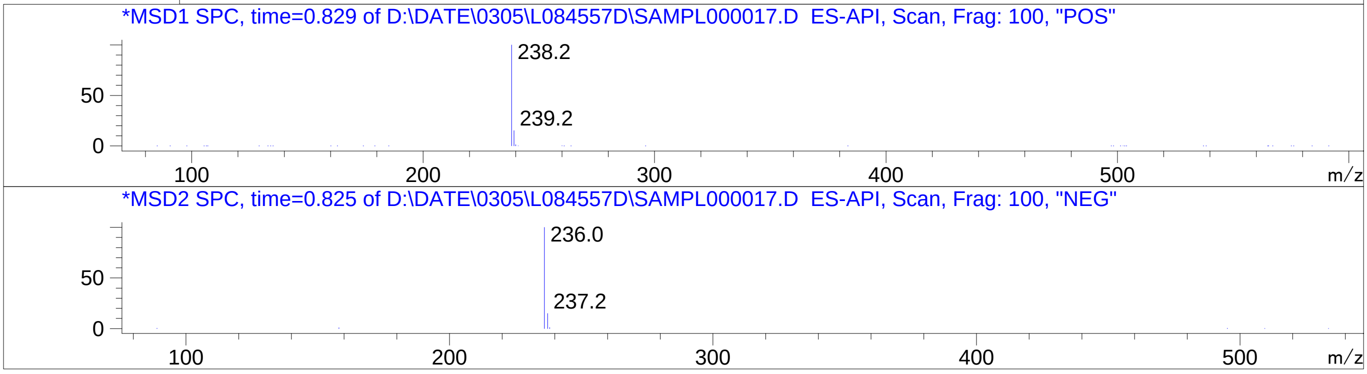

Supplement: Supplementary file 2. [file elife-53779-supp2.zip › mt_vls_62_compounds_QC_data/Compound_36_Z1203061587/Z1203061587_21507834.PDF]
