# Supplementary material for: Structure-based discovery of potent and selective melatonin receptor agonists
Source: eLife. 2020 Mar 2;9:e53779. doi: 10.7554/eLife.53779 (PMC7080406; doi:10.7554/eLife.53779)
Supplement: Supplementary file 2. [file elife-53779-supp2.zip › mt_vls_62_compounds_QC_data/Compound_44_Z1262254255/Z1262254255_5576804.PDF]

MaxPeak: 100.00%
Ret_Time: 0.613 min

R791347

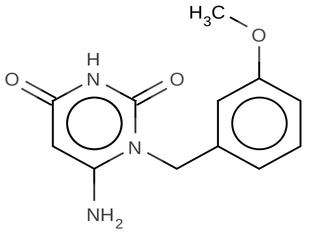

Mol Wt 247.25
Exact Mass 247.1

#	Time	Area%
1	0.613	100.00

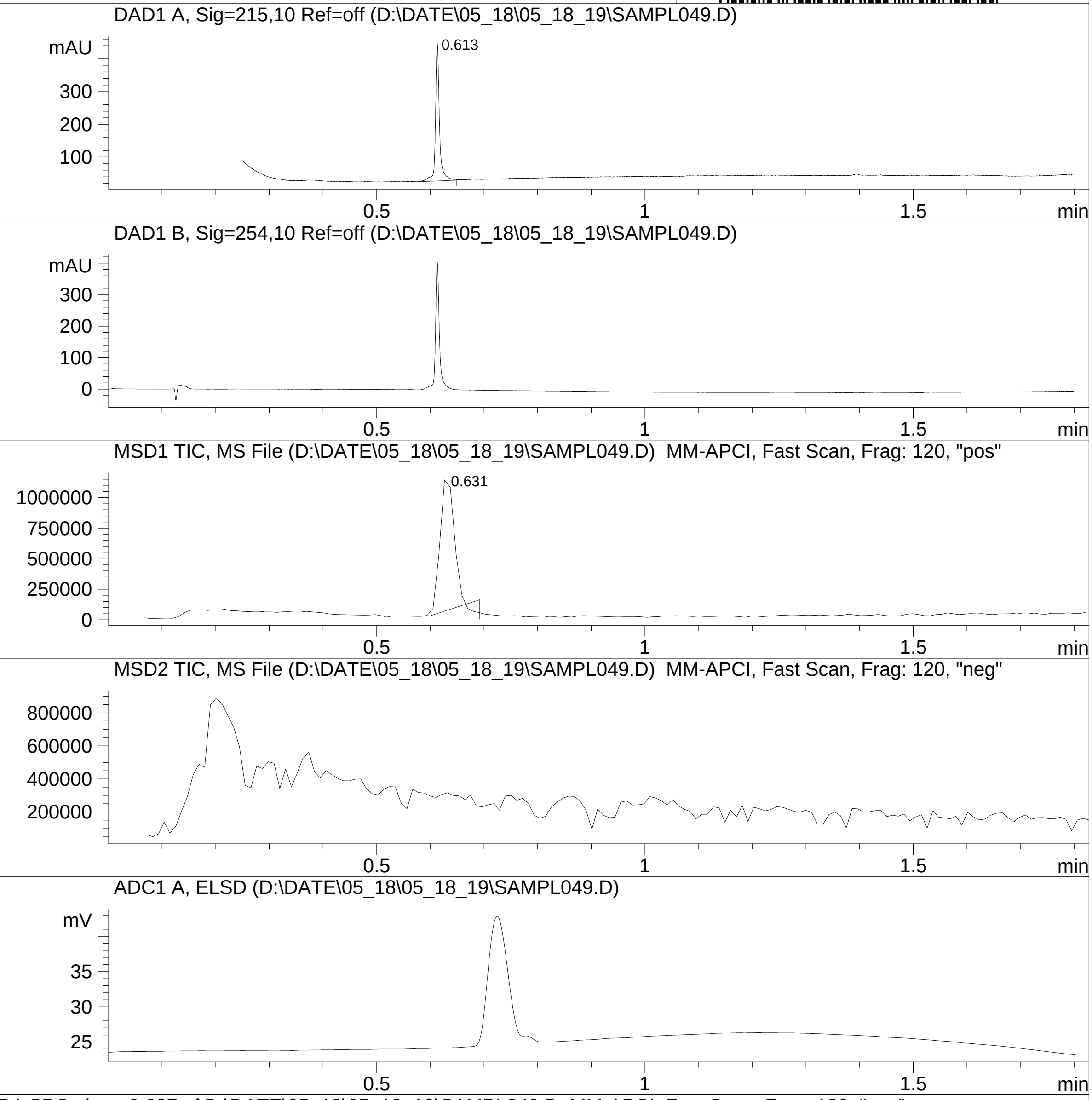

RT 0.631

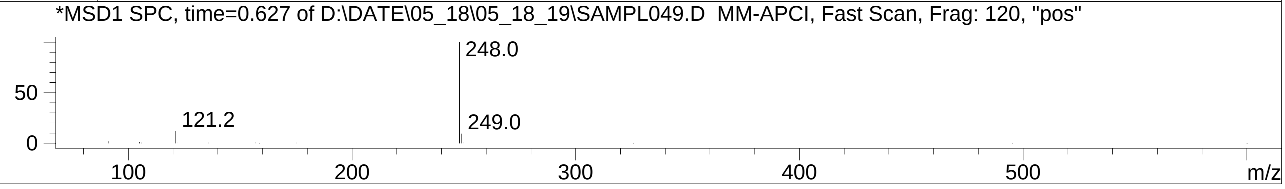

Inj.Date 5/18/2010 O P1-C-02 RR Acq. Method C:\CHEM32\-> ->
