# Supplementary material for: Structure-based discovery of potent and selective melatonin receptor agonists
Source: eLife. 2020 Mar 2;9:e53779. doi: 10.7554/eLife.53779 (PMC7080406; doi:10.7554/eLife.53779)
Supplement: Supplementary file 2. [file elife-53779-supp2.zip › mt_vls_62_compounds_QC_data/Compound_47_KO_1/9E-001-64730-NMR.pdf]

9E-001-64730

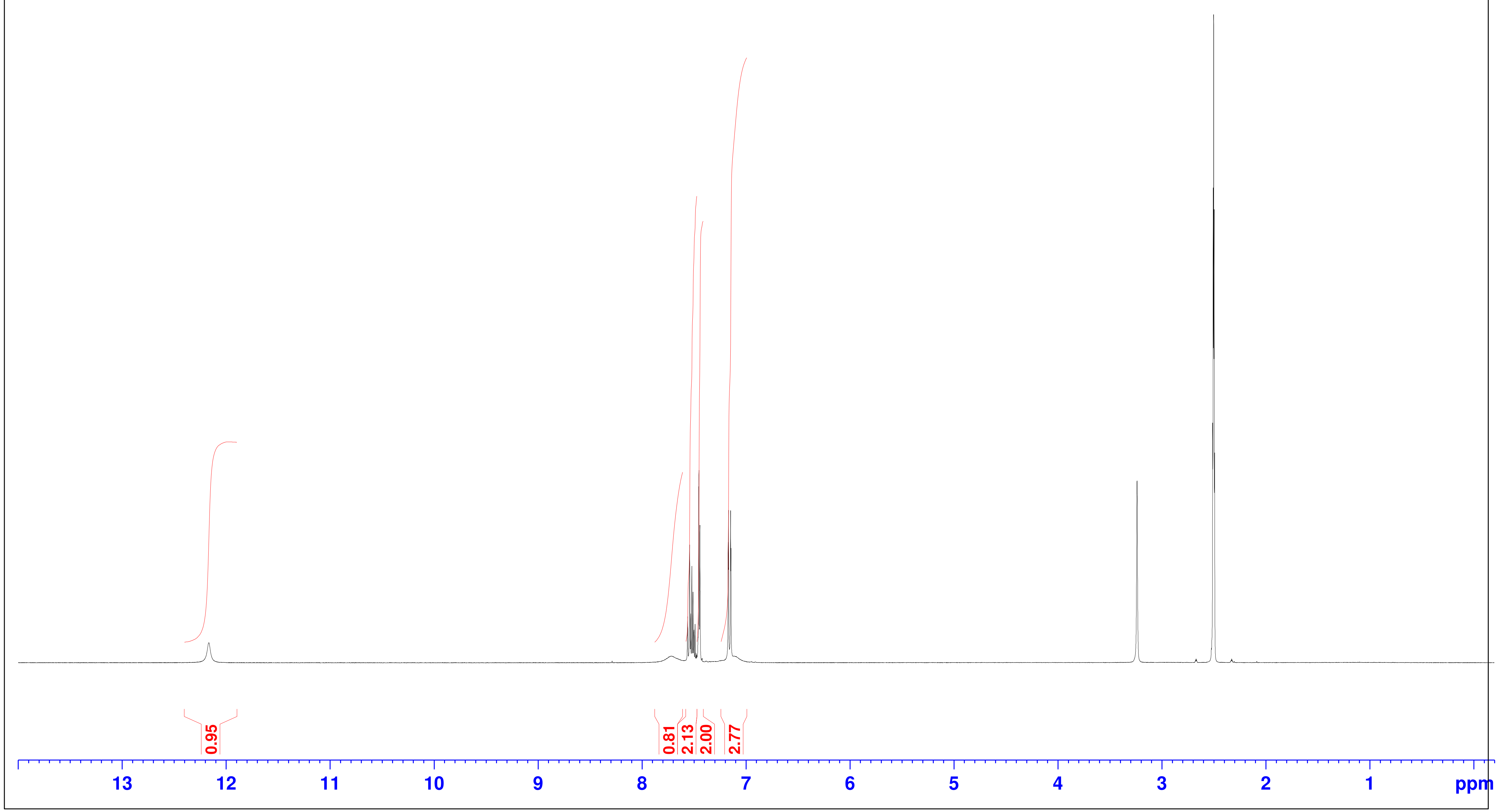

Current Data Parameters	
NAME	Sep05-2019
EXPNO	20
PROCNO	1
F2 - Acquisition Parameters	
Date_	20190905
Time	10.46 h
INSTRUM	spect
PROBHD	Z8246_0097 (PH
PULPROG	zg30
TD	65536
SOLVENT	DMSO
NS	16
DS	0
SWH	8223.685 Hz
FIDRES	0.250967 Hz
AQ	3.9845889 sec
RG	456
DW	60.800 use
DE	15.87 use
TE	309.1 K
D1	1.00000000 sec
TD0	1
SFO1	400.1328009 MHz
NUC1	1H
P1	14.80 use
PLW1	9.19999981 W
F2 - Processing parameters	
SI	65536
SF	400.1300034 MHz
WDW	EM
SSB	0
LB	0.30 Hz
GB	0
PC	1.40
