# Supplementary material for: Structure-based discovery of potent and selective melatonin receptor agonists
Source: eLife. 2020 Mar 2;9:e53779. doi: 10.7554/eLife.53779 (PMC7080406; doi:10.7554/eLife.53779)
Supplement: Supplementary file 2. [file elife-53779-supp2.zip › mt_vls_62_compounds_QC_data/Compound_47_KO_1/COA 9E-001.pdf]

Key Organics
Chemistry | Innovation | Quality
Home of BIONET

Highfield Road Industrial Estate
Camelford Cornwall PL32 9RA
United Kingdom
Telephone +44(0)1840 212137
Fax +44(0)1840 213712
enquiries@keyorganics.net

Certificate of Analysis

Key Organics Sample ID: **9E-001**

Batch No: **64730**

Product Name: **4-(2-chlorobenzoyl)-1H-pyrrole-2-carboxamide**

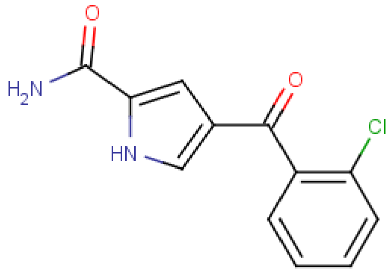

CAS Number: 338976-88-8

Molecular Formula: $C_{12}H_9ClN_2O_2$

Molecular Weight: 248.67

Date Printed: Thursday, September 05, 2019

Test	Specification	Result
Identification (NMR)	^{1}H NMR is consistent with structure	Complies
Purity (NMR)	Assessed by NMR ≥90%	Complies

Conclusion The material complies with the specification

Authorised

Project Manager

Registered in England No. 2055784. Registered Office: 12 Upper Belgrave Street, London, SW1X 8BA VAT Reg. No. GB 441 2642 78
