# Supplementary material for: Structure-based discovery of potent and selective melatonin receptor agonists
Source: eLife. 2020 Mar 2;9:e53779. doi: 10.7554/eLife.53779 (PMC7080406; doi:10.7554/eLife.53779)

L693633$2

MaxPeak: 100.00%
Ret_Time: 0.859 min

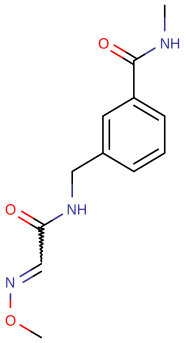

Mol Wt 249.27

Exact Mass 249.12

#	Time	Area%
1	0.859	100.00

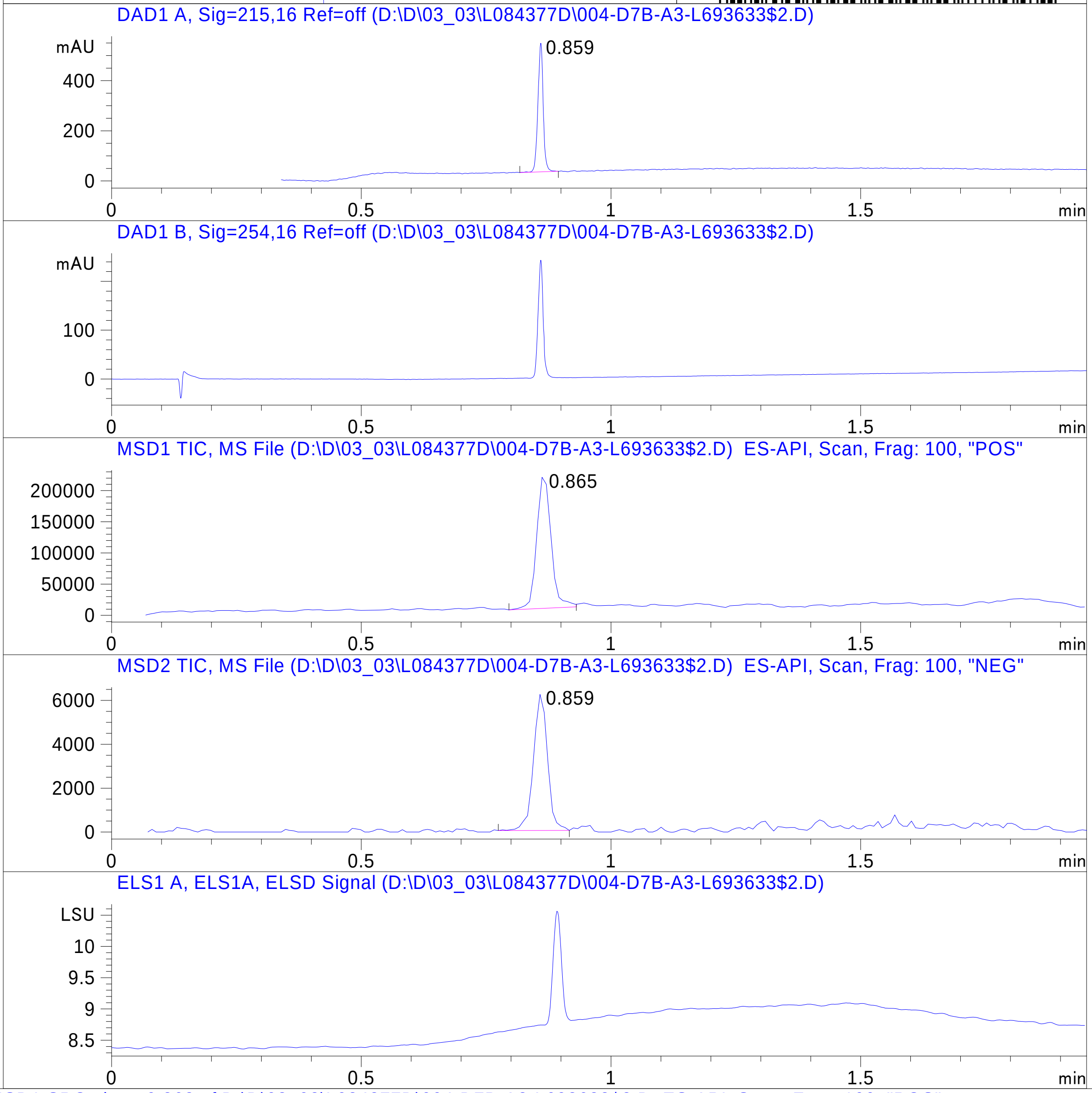

RT 0.865

RT 0.859

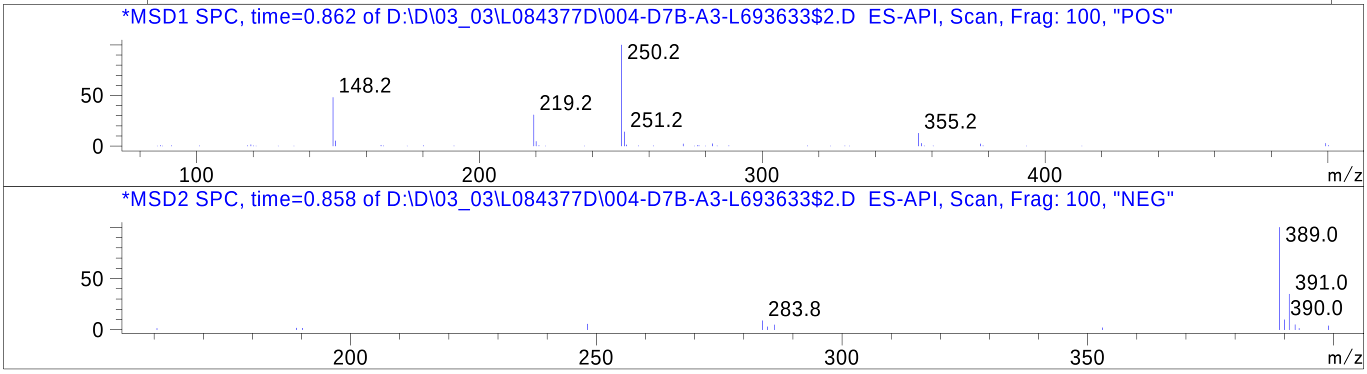

Supplement: Supplementary file 2. [file elife-53779-supp2.zip › mt_vls_62_compounds_QC_data/Compound_5_Z2161843026/Z2161843026_21481719.PDF]
