# Supplementary material for: Structure-based discovery of potent and selective melatonin receptor agonists
Source: eLife. 2020 Mar 2;9:e53779. doi: 10.7554/eLife.53779 (PMC7080406; doi:10.7554/eLife.53779)
Supplement: Supplementary file 2. [file elife-53779-supp2.zip › mt_vls_62_compounds_QC_data/Compound_51_STL146796.pdf]

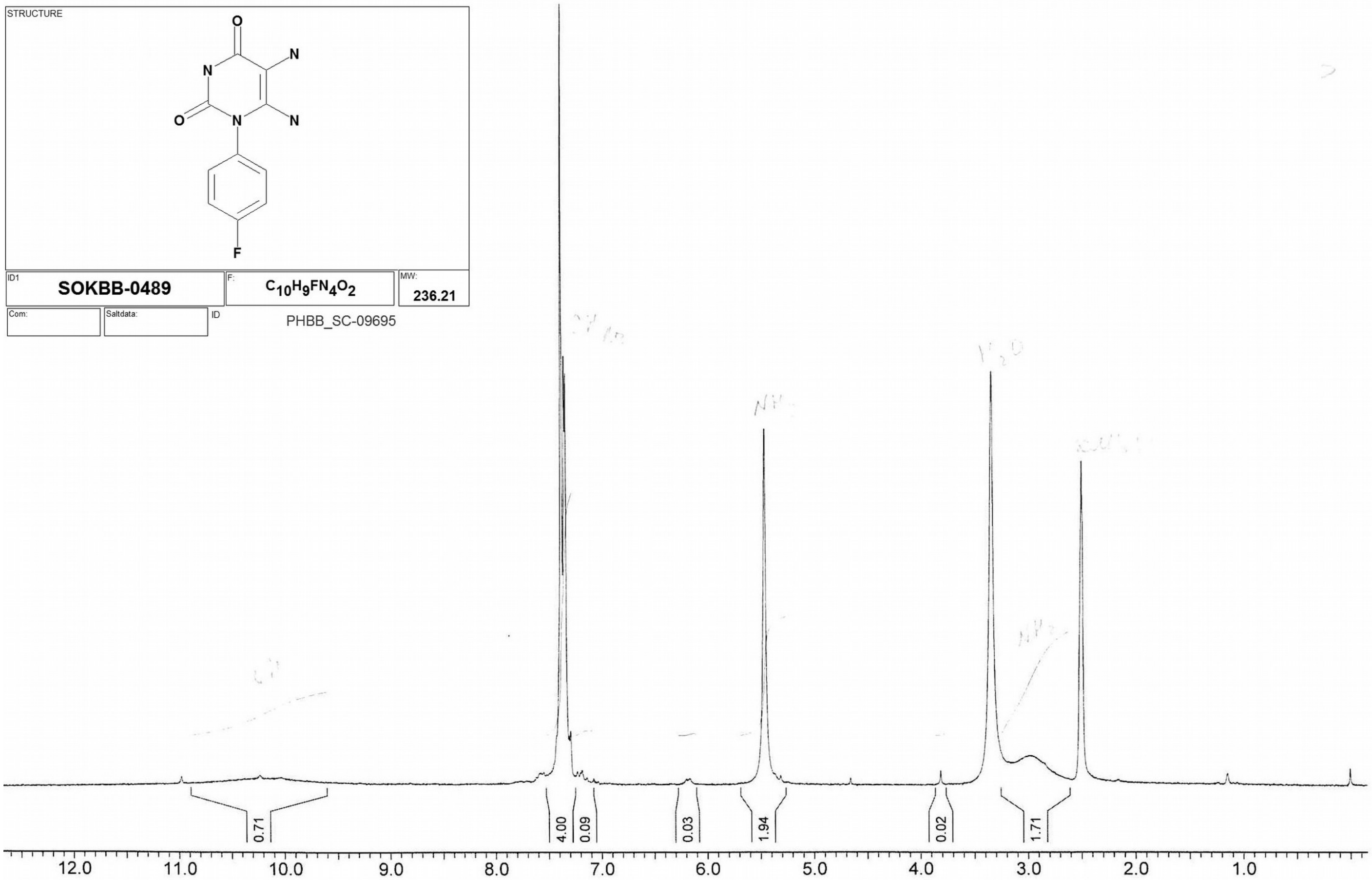

File name: SOKBB-0489.dx	Operator: root	SF: 200.1300 MHz	NSC: 16	PW: 0.00 usec, RG: 500	SI: 16384
	Solvent: DMSO	SW: 3019 Hz	TE: 300 K	AQ: 1.36 sec, RD: 0.00 sec	26.10.[illegible]0
