# Supplementary material for: Structure-based discovery of potent and selective melatonin receptor agonists
Source: eLife. 2020 Mar 2;9:e53779. doi: 10.7554/eLife.53779 (PMC7080406; doi:10.7554/eLife.53779)
Supplement: Supplementary file 2. [file elife-53779-supp2.zip › mt_vls_62_compounds_QC_data/Compound_55_KO_2/GE-0720-101179-NMR.pdf]

GE-0720-101179

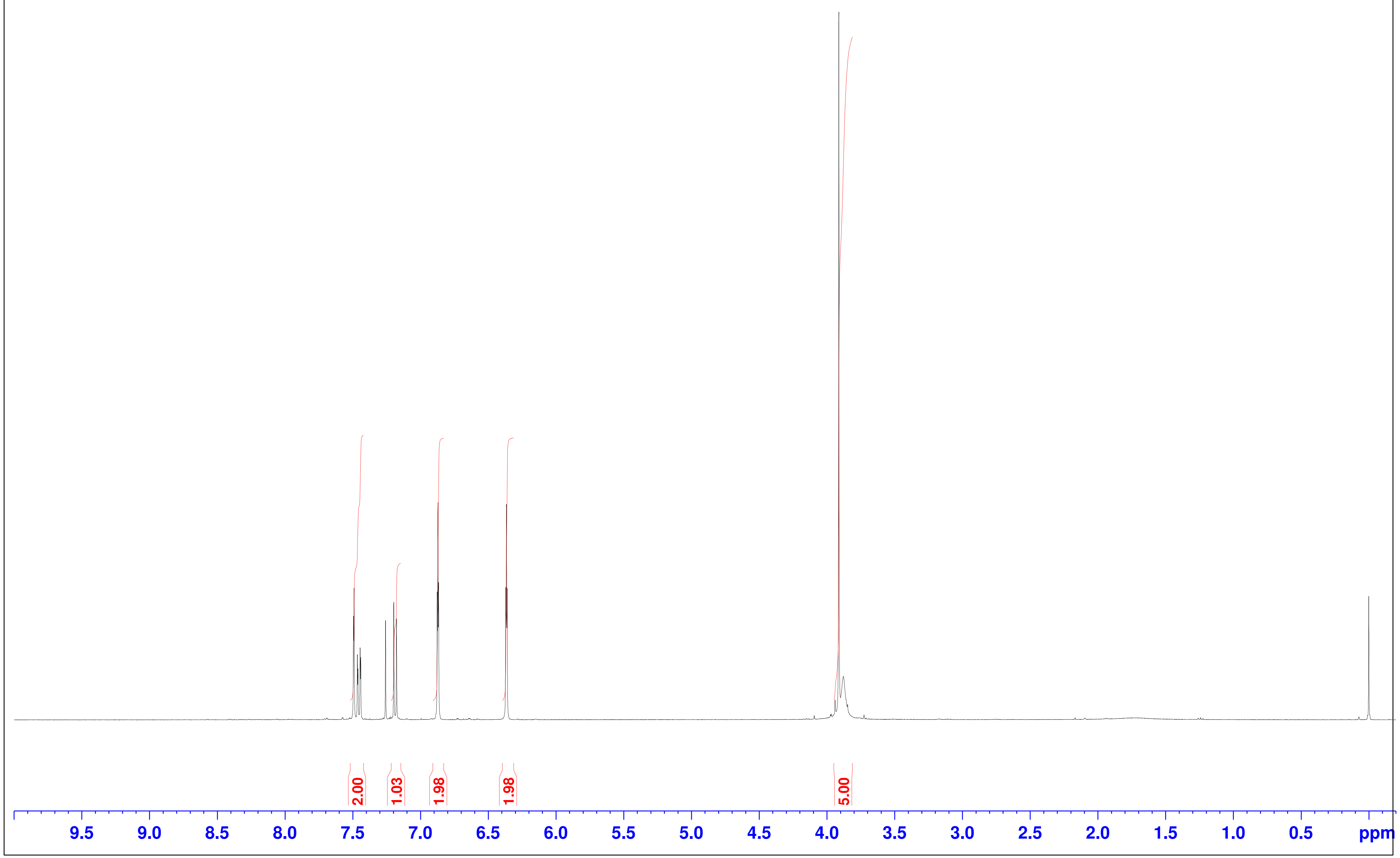

Current Data Parameters

NAME	Sep05-2019	
EXPNO	10	
PROCNO	1	

F2 - Acquisition Parameters

Date_	20190905	
Time	10.21	h
INSTRUM	spect	
PROBHD	Z8246_0097 (PH	
PULPROG	zg30	
TD	65536	
SOLVENT	CDCl3	
NS	16	
DS	0	
SWH	8223.685	Hz
FIDRES	0.250967	Hz
AQ	3.9845889	sec
RG	456	
DW	60.800	use
DE	15.87	use
TE	299.9	K
D1	1.00000000	sec
TD0	1	
SFO1	400.1328009	MHz
NUC1	1H	
P1	14.80	use
PLW1	9.19999981	W

F2 - Processing parameters

SI	65536	
SF	400.1300105	MHz
WDW	EM	
SSB	0	
LB	0.30	Hz
GB	0	
PC	1.40	
