# Supplementary material for: Structure-based discovery of potent and selective melatonin receptor agonists
Source: eLife. 2020 Mar 2;9:e53779. doi: 10.7554/eLife.53779 (PMC7080406; doi:10.7554/eLife.53779)

MaxPeak: 100.00%
Ret_Time: 0.964 min

L693616$4

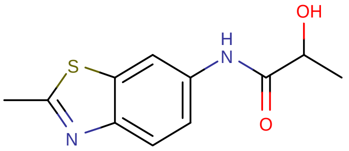

Mol Wt 236.29

Exact Mass 236.07

#	Time	Area%
1	0.964	100.00

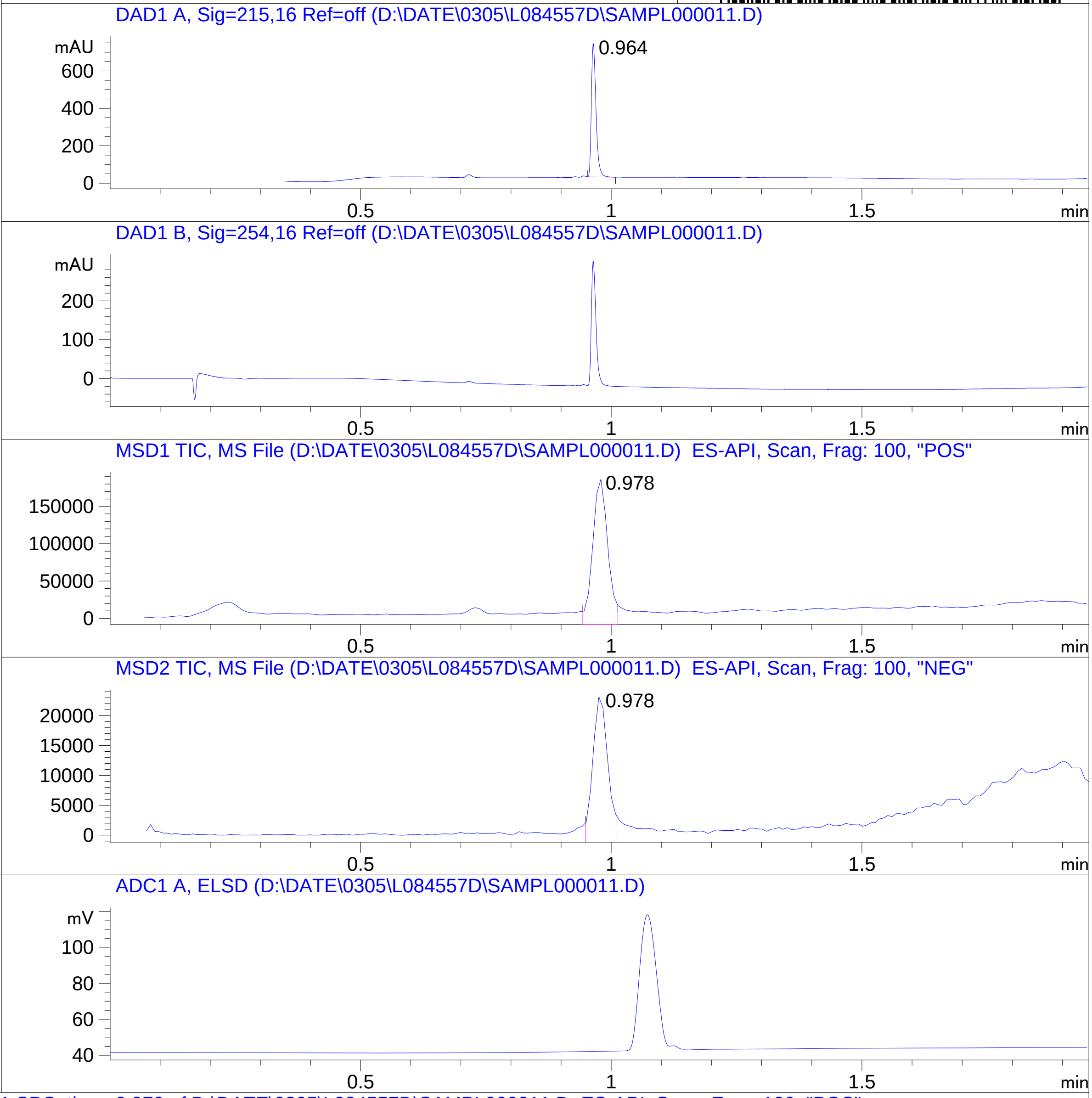

RT 0.978

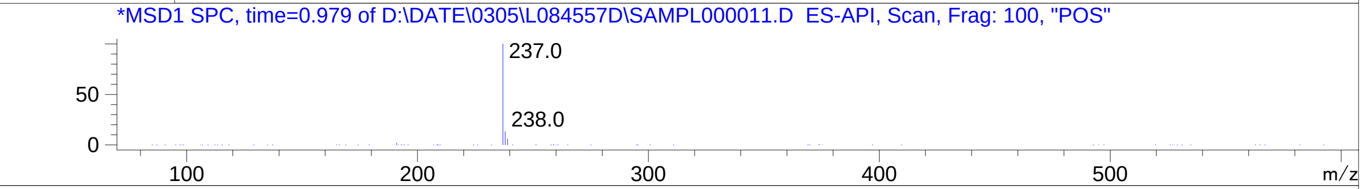

RT 0.978

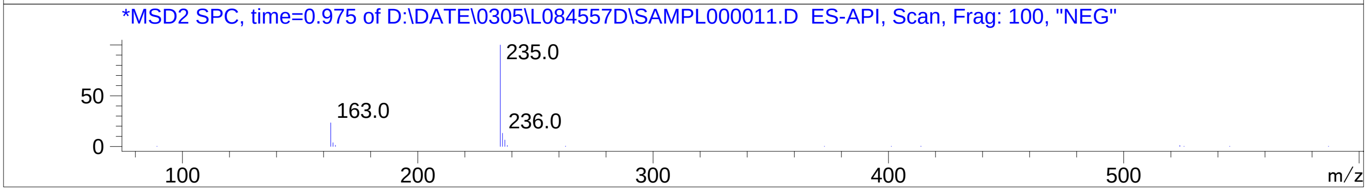

Supplement: Supplementary file 2. [file elife-53779-supp2.zip › mt_vls_62_compounds_QC_data/Compound_6_Z2252522873/Z2252522873_21507828.PDF]
