# Supplementary material for: Structure-based discovery of potent and selective melatonin receptor agonists
Source: eLife. 2020 Mar 2;9:e53779. doi: 10.7554/eLife.53779 (PMC7080406; doi:10.7554/eLife.53779)
Supplement: Supplementary file 2. [file elife-53779-supp2.zip › mt_vls_62_compounds_QC_data/Compound_8_Z1480824591/Z1480824591.docx]

CERTIFICATE of ANALYSIS

1. Identification

| Structure | 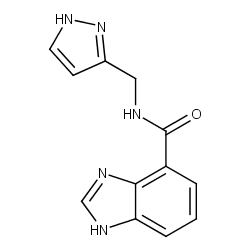 |
| --- | --- |
| Codes | Z1480824591 |
| Name | N-[(1H-pyrazol-3-yl)methyl]-1H-1,3-benzodiazole-4-carboxamide |
| Formula | C12H11N5O |
| Formula weight | 241.24864 |
| CAS number |  |

2. Description

| Appearance | crystalline powder |
| --- | --- |
| Color | pink |
| Melting point, °C | N/A |
| Boiling point, °C | Not determined |

3. NMR spectra

| File name, *wmf | H0935656 |
| --- | --- |
| Identity | Agrees with the structure |

4. LCMS data

| File name, *pdf | N/A |
| --- | --- |
| UV Area, % | 91 |

5. Comments

| Comments | No special comments. |
| --- | --- |

SAFETY DATA SHEET

**1. Product Information**

Product Name: **N-[(1H-pyrazol-3-yl)methyl]-1H-1,3-benzodiazole-4-carboxamide**

Product Catalogue Number: Z1480824591

CAS number:

Information of company: ENAMINE Ltd.

78 Chervonotkatska Street

02094 Kyiv

Ukraine

Telephone: +38 044 495 88 17

E-mail address: sds@enamine.net

**Experimental Product for Research&Development Use Only. Not for Drug, Household or Other Use**

**2. HAZARDS IDENTIFICATION**

2.1 Classification of the substance / mixture

Not a hazardous substance or mixture according to 29 CFR 1910 (OSHA HCS) and Regulation (EC) No. 1272/2008.

2.2 Label elements

The product does not need to be labelled in accordance with EC/OSHA HCS directives or respective national laws.

Special note: Excessive pressure may build up in a container. Open container with caution to prevent container damage and accidental release.

2.3 Other hazards

N/A

**3. COMPOSITION/INFORMATION ON INGREDIENTS**

Product Name: **N-[(1H-pyrazol-3-yl)methyl]-1H-1,3-benzodiazole-4-carboxamide**

Formula: C12H11N5O

Molecular Weight: 241.24864

Hazardous ingredients according to 29 CFR 1910 (OSHA HCS) and Regulation (EC) No. 1272/2008.

Component: N-[(1H-pyrazol-3-yl)methyl]-1H-1,3-benzodiazole-4-carboxamide

Concentration, %: «91»

For the full text of the H-Statements mentioned in this Section, see Section 2.2.

**4 - First Aid Measures**

__________________________________________________________________

AFTER INHALATION

If inhaled, remove to fresh air. If not breathing give artificial respiration. If breathing is difficult, give oxygen.

AFTER SKIN CONTACT

In case of skin contact, flush with copious amounts of water for at least 15 minutes. Remove contaminated clothing and shoes.

Call a physician.

AFTER EYE CONTACT

In case of contact with eyes, flush with copious amounts of water for at least 15 minutes. Assure adequate flushing by separating the eyelids with fingers. Call a physician.

AFTER INGESTION

If swallowed, wash out mouth with water provided person is conscious. Call a physician.

_______________________________________________________________________

**5 - Fire Fighting Measures**

______________________________________________________________________

EXTINGUISHING MEDIA

Suitable: Water spray. Carbon dioxide, dry chemical powder, or appropriate foam.

SPECIAL RISKS

Specific Hazard(s): Emits toxic fumes under fire conditions.

SPECIAL PROTECTIVE EQUIPMENT FOR FIREFIGHTERS

Wear self-contained breathing apparatus and protective clothing to prevent contact with skin and eyes.

________________________________________________________________________

**6 - Accidental Release Measures**

_________________________________________________________________________

PERSONAL PRECAUTION PROCEDURES TO BE FOLLOWED IN CASE OF LEAK OR SPILL

Evacuate area.

PROCEDURE(S) OF PERSONAL PRECAUTION(S)

Wear self-contained breathing apparatus, rubber boots, and heavy

rubber gloves.

METHODS FOR CLEANING UP

Wipe dry, place a rag in a bag and hold for waste disposal. Avoid fumes inhaling. Ventilate area and wash spill site after material pickup is complete.

_________________________________________________________________________________

**7 - Handling and Storage**

_________________________________________________________________________________

HANDLING

Directions for Safe Handling: Do not breathe vapor. Avoid contact with eyes, skin, and clothing. Avoid prolonged or repeated exposure.

STORAGE

Conditions of Storage: Keep tightly closed. Keep in room temperature.

Expire date: not available, reanalysis is required no more than once a year.

SPECIAL REQUIREMENTS: -

____________________________________________________________________________________

**8 - Exposure Controls / Personal Protection**

____________________________________________________________________________________

ENGINEERING CONTROLS

Safety shower and eye bath. Mechanical exhaust required.

GENERAL HYGIENE MEASURES

Wash thoroughly after handling.

PERSONAL PROTECTIVE EQUIPMENT

Respiratory Protection: Government approved respirator.

Hand Protection: Compatible chemical-resistant gloves.

Eye Protection: Chemical safety goggles.

____________________________________________________________________________________

**9 - Physical and Chemical Properties**

___________________________________________________________________________________

Property Value At Temperature or Pressure

pH N/A

MP/MP Range, ˚C N/A

Flash Point N/A

Flammability N/A

Autoignition Temp N/A

Oxidizing Properties N/A

Explosive Properties N/A

Explosion Limits N/A

Vapor Pressure N/A

SG/Density N/A

Partition Coefficient N/A

Viscosity N/A

Vapor Density N/A

Saturated Vapor Conc. N/A

Evaporation Rate N/A

Bulk Density N/A

Decomposition Temp. N/A

Solvent Content N/A

Water Content N/A

Surface Tension N/A

Conductivity N/A

Miscellaneous Data N/A

Solubility N/A

____________________________________________________________________________________**10 - Stability and Reactivity**

____________________________________________________________________________________

STABILITY

Stable: Stable.

Conditions of Instability:

Materials to Avoid: Strong oxidizing agents, Strong acids.

HAZARDOUS DECOMPOSITION PRODUCTS

Hazardous Decomposition Products: Carbon monoxide, Carbon dioxide, Nitrogen oxides.

HAZARDOUS POLYMERIZATION

Hazardous Polymerization: Will not occur

____________________________________________________________________________________

**11 - Toxicological Information**

____________________________________________________________________________________

N/A

Toxicological properties were not fully evaluated.

___________________________________________________________________________________

**12 - Ecological Information**

___________________________________________________________________________________

N/A

Toxicological properties were not fully evaluated.

___________________________________________________________________________________

**13 - Disposal Considerations**

___________________________________________________________________________________

SUBSTANCE DISPOSAL

Contact a licensed professional waste disposal service to dispose of this material. Dissolve or mix the material with a combustible solvent and burn in a chemical incinerator equipped with an afterburner and scrubber. Observe all federal, state, and local environmental regulations.

___________________________________________________________________________________

**14 - Transport Information**

___________________________________________________________________________________

**14.1 UN number**

DOT: - IMDG: - IATA: -

14.2 **UN proper shipping name**

DOT: Not dangerous goods

IMDG: Not dangerous goods

IATA: Not dangerous goods

**14.3 Transport hazard class(es)**

DOT: - IMDG: - IATA: -

**14.4 Packaging group**

DOT: - IMDG: - IATA: -

**14.5 Environmental hazards**

DOT: no IMDG: no IATA: no

**14.6 Special precautions for user**

No data available

___________________________________________________________________________________

**15 - Regulatory Information**

___________________________________________________________________________________

Safety Statements: Do not breathe dust. Avoid contact with skin and eyes.

________________________________________________________________________________

__

**16 - Other Information**

___________________________________________________________________________________

For R&D use only. Not for drug, household or other uses.

This is an experimental product whose properties are not fully evaluated yet. The information contained herein is based on the present state of our knowledge and therefore does not guarantee certain properties. Recipients of our product must take responsibility for observing existing laws and regulations.

_________________________________________________________________________________

End of Safety Data Sheet
