# Supplementary material for: Structure-based discovery of potent and selective melatonin receptor agonists
Source: eLife. 2020 Mar 2;9:e53779. doi: 10.7554/eLife.53779 (PMC7080406; doi:10.7554/eLife.53779)

L693644$12

MaxPeak: 97.26%
Ret_Time: 0.691 min

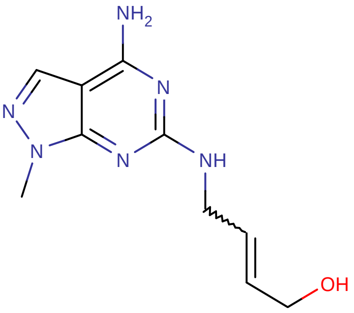

Mol Wt 234.26

Exact Mass 234.13

#	Time	Area%
1	0.691	97.26
2	0.791	2.74

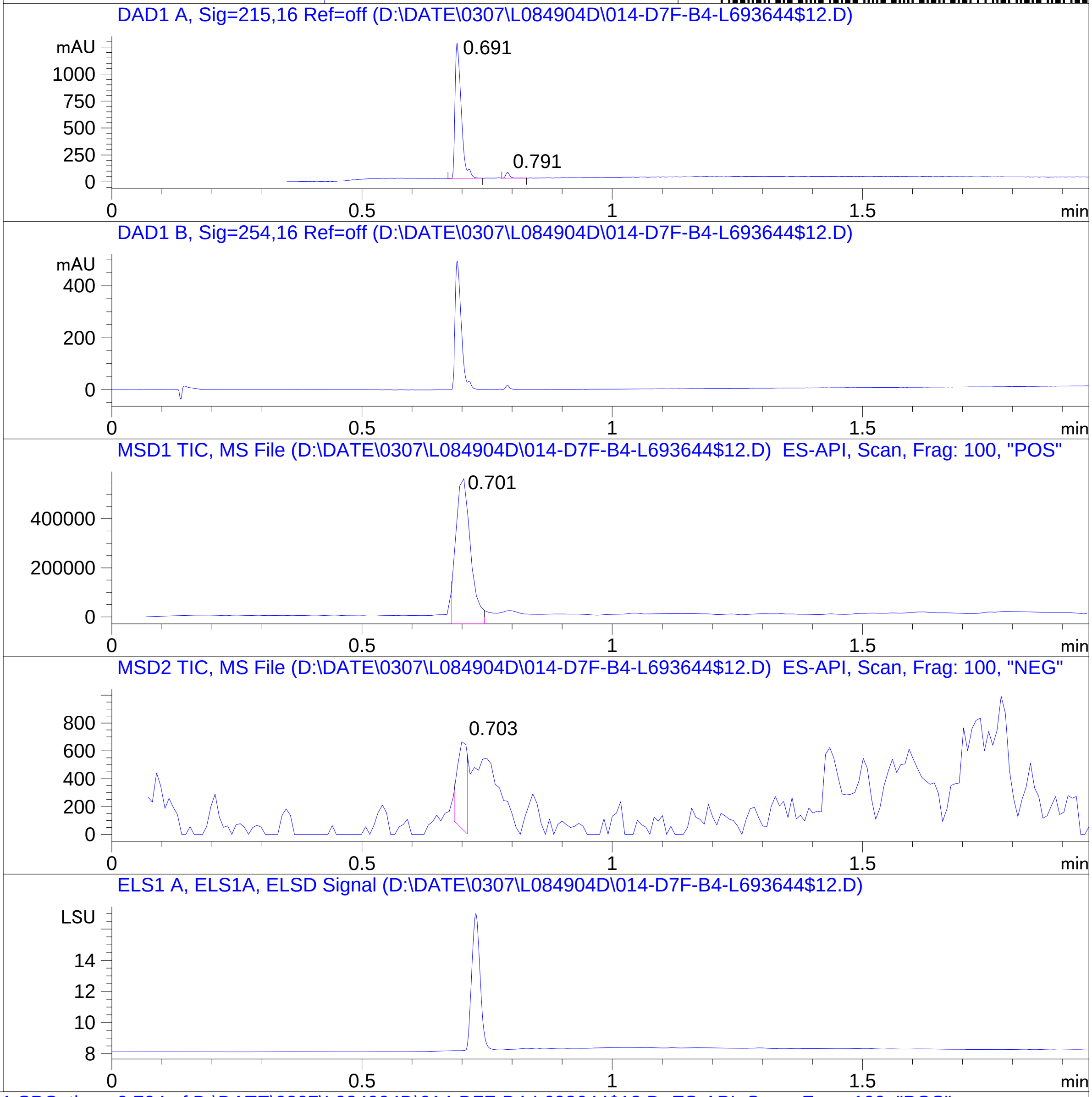

RT 0.701

RT 0.703

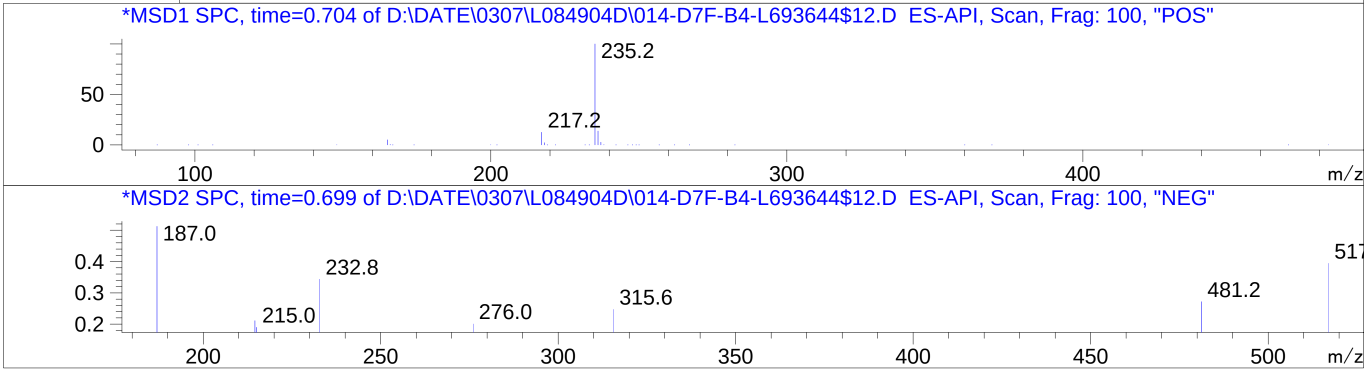

Supplement: Supplementary file 2. [file elife-53779-supp2.zip › mt_vls_62_compounds_QC_data/Compound_9_Z2614615475/Z2614615475_21523441.PDF]
