# Supplementary figures and images for: Structure-based discovery of potent and selective melatonin receptor agonists
Source: eLife. 2020 Mar 2;9:e53779. doi: 10.7554/eLife.53779 (PMC7080406; doi:10.7554/eLife.53779)

L693631\$12

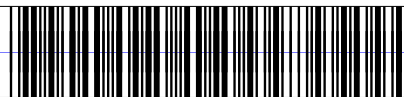

MaxPeak: 90.02%  
Ret\_Time: 0.985 min

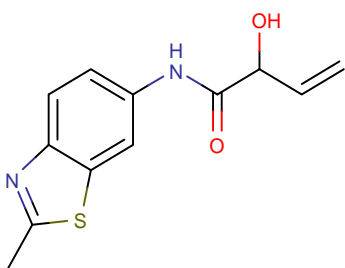

Mol Wt 248.3  
Exact Mass 248.07

| # | Time  | Area% |
|---|-------|-------|
| 1 | 0.687 | 2.63  |
| 2 | 0.985 | 90.02 |
| 3 | 1.020 | 3.51  |
| 4 | 1.162 | 3.84  |

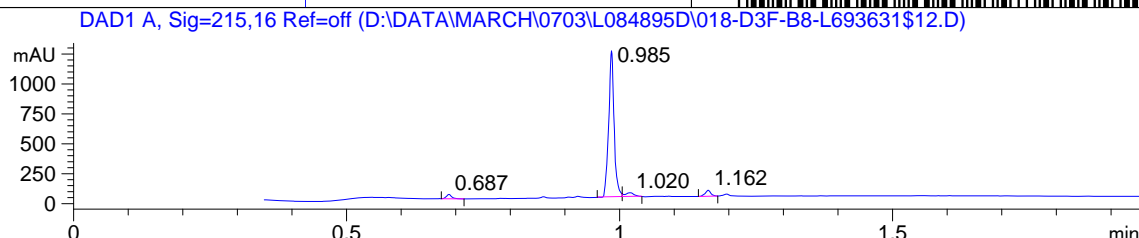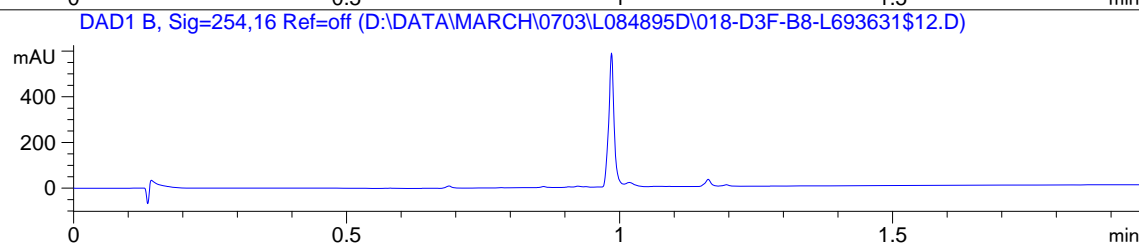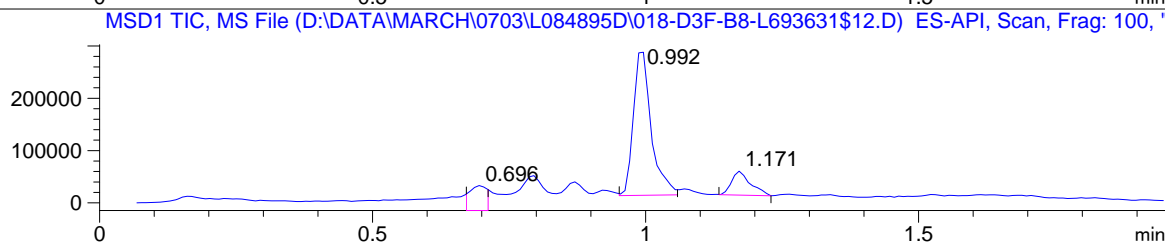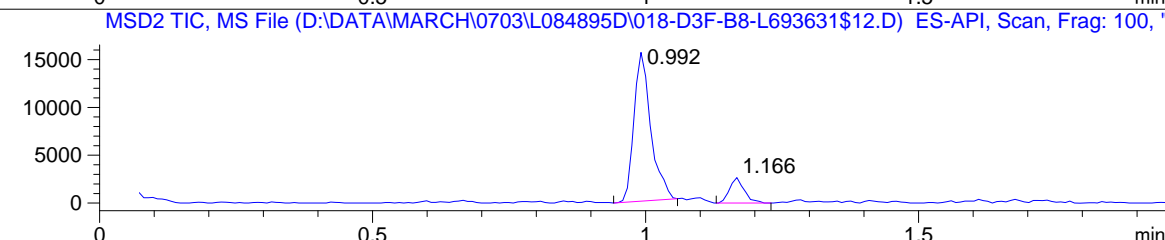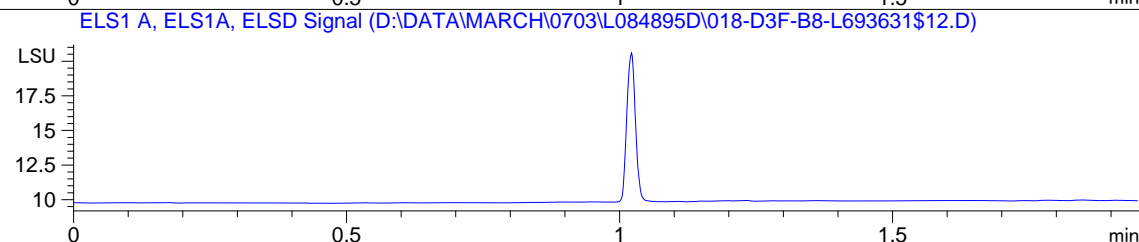

RT 0.696

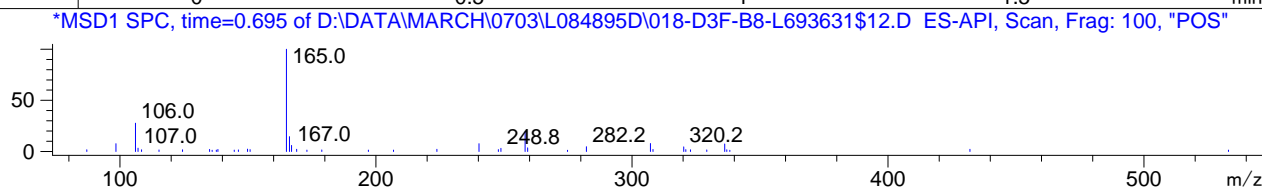

RT 0.992

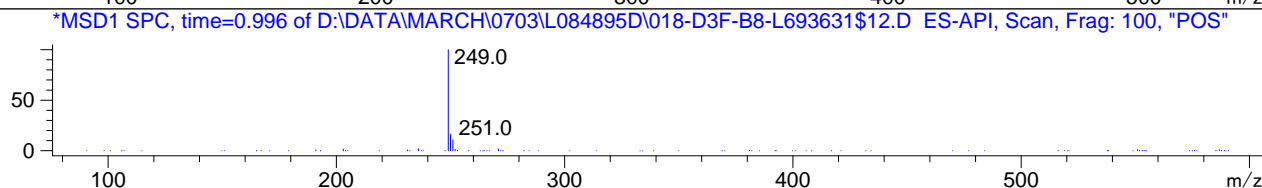

RT 1.171

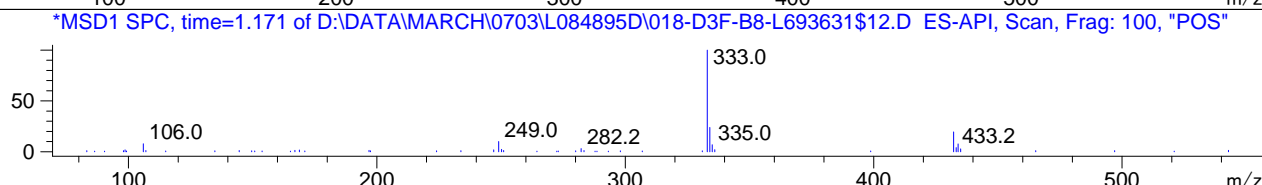

RT 0.992

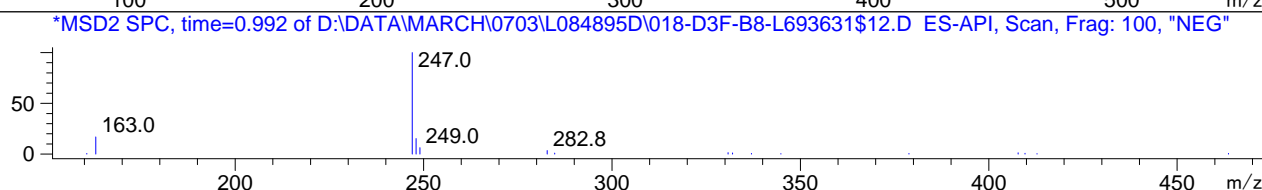

RT 1.166

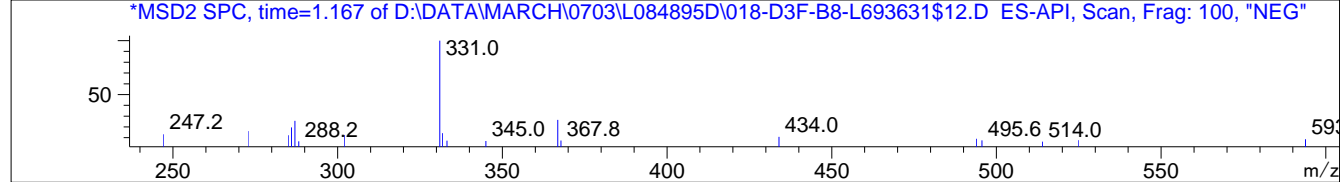

Supplement: Supplementary file 2. [file elife-53779-supp2.zip › mt_vls_62_compounds_QC_data/Compound_1_Z2228498436/Z2228498436_21523254.PDF]

L693617\$2

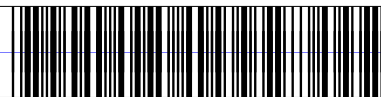

MaxPeak: 61.29%  
Ret\_Time: 0.759 min

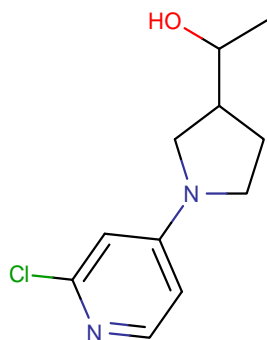

Mol Wt 226.7  
Exact Mass 226.11

| # | Time  | Area% |
|---|-------|-------|
| 1 | 0.740 | 38.71 |
| 2 | 0.759 | 61.29 |

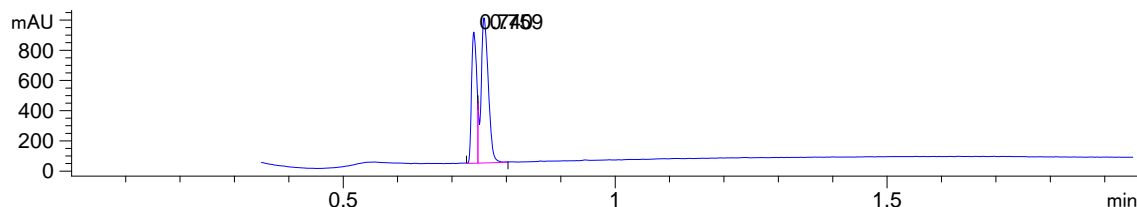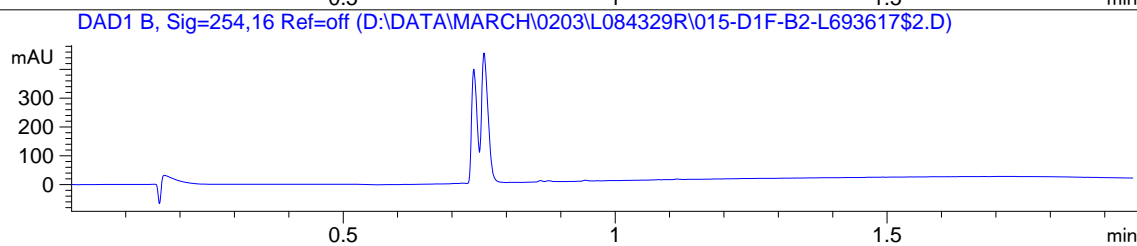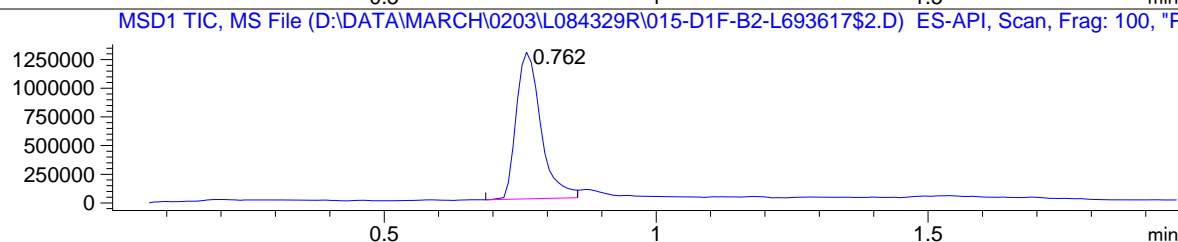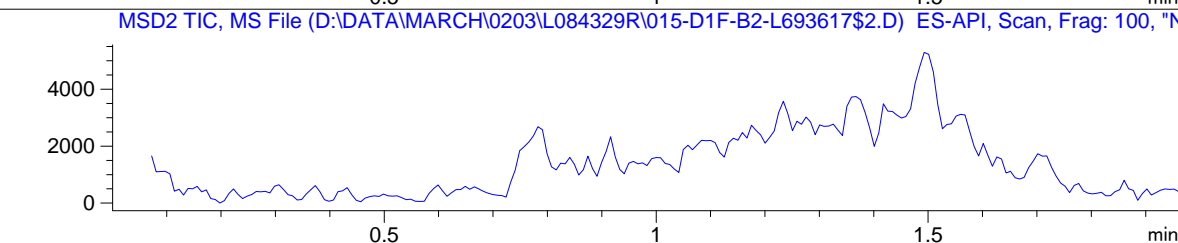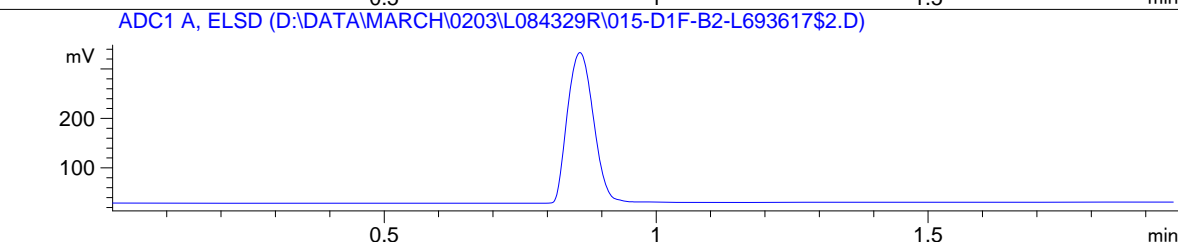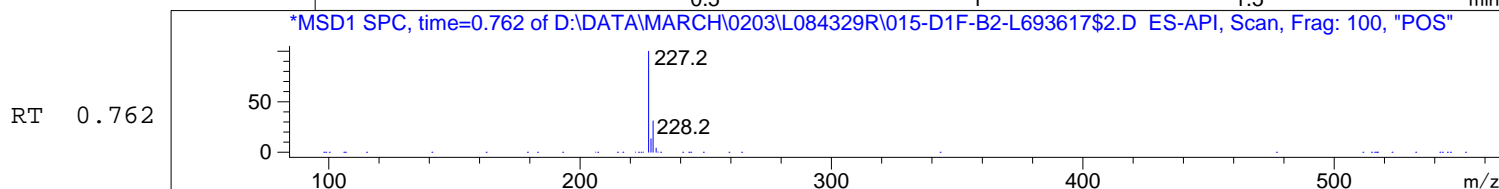

Supplement: Supplementary file 2. [file elife-53779-supp2.zip › mt_vls_62_compounds_QC_data/Compound_10_Z1627633848/Z1627633848_21478626.PDF]

MaxPeak: 95.11%  
Ret\_Time: 0.577 min

L693643\$8

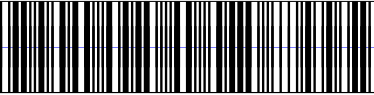

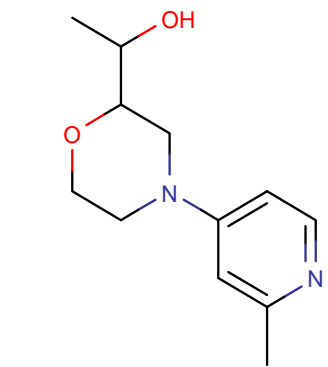

Mol Wt 222.28  
Exact Mass 222.16

| # | Time  | Area% |
|---|-------|-------|
| 1 | 0.577 | 95.11 |
| 2 | 0.643 | 4.89  |

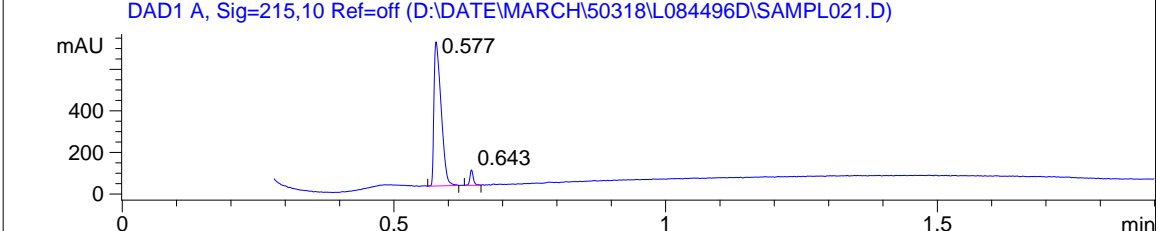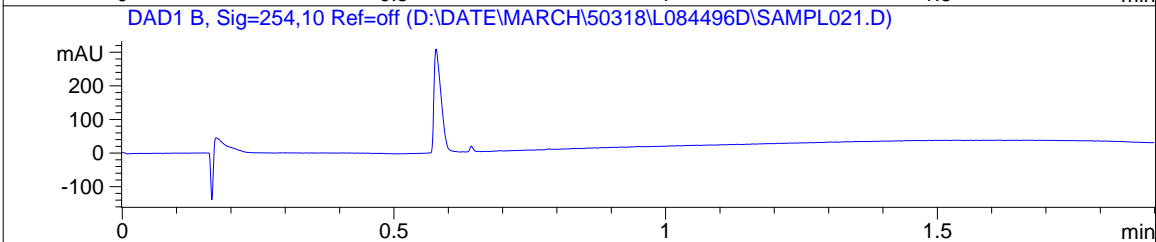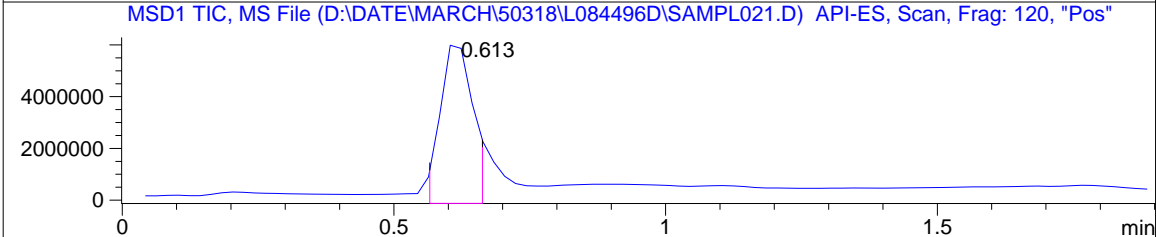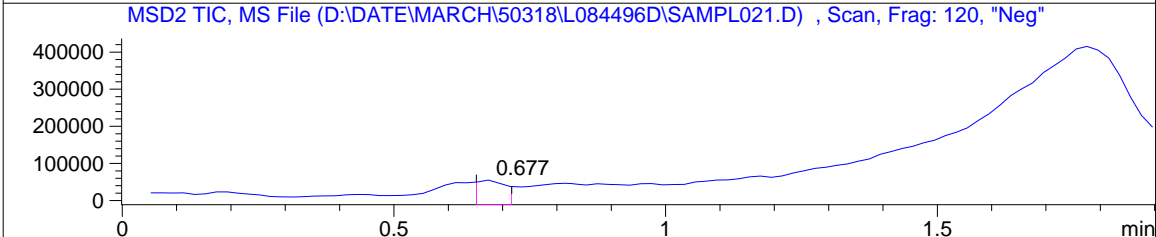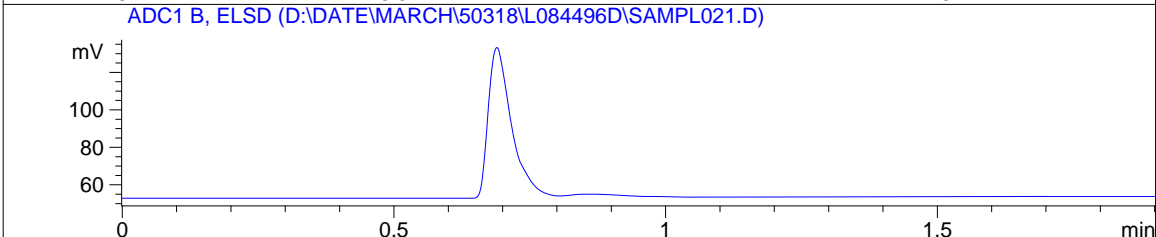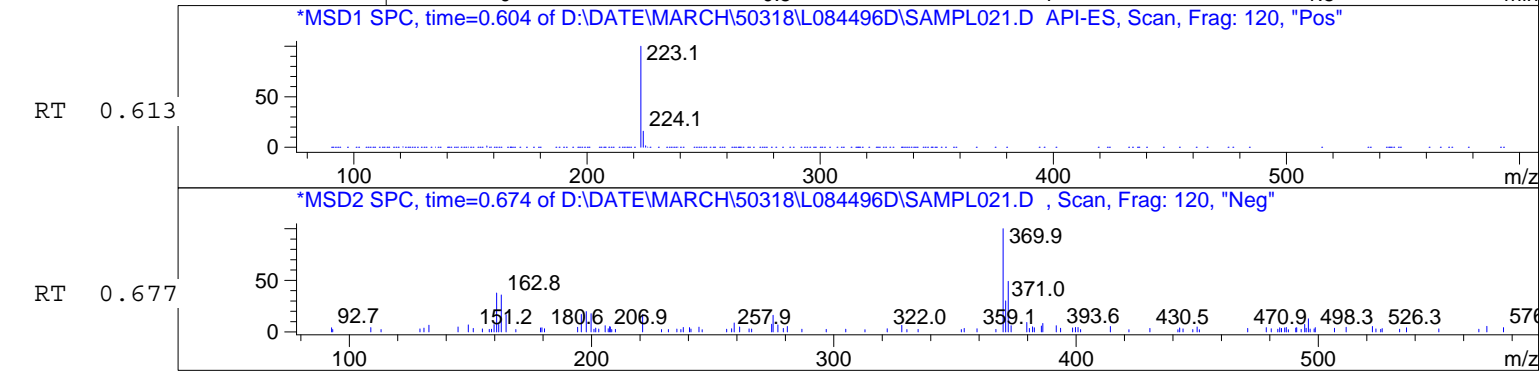

Supplement: Supplementary file 2. [file elife-53779-supp2.zip › mt_vls_62_compounds_QC_data/Compound_12_Z2348423828/Z2348423828_21487768.PDF]

MaxPeak: 100.00%  
Ret\_Time: 0.883 min

L693638\$6

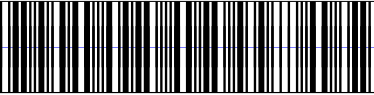

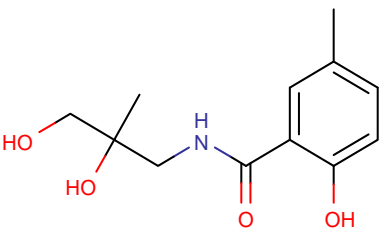

Mol Wt 239.27  
Exact Mass 239.13

| # | Time  | Area%  |
|---|-------|--------|
| 1 | 0.883 | 100.00 |

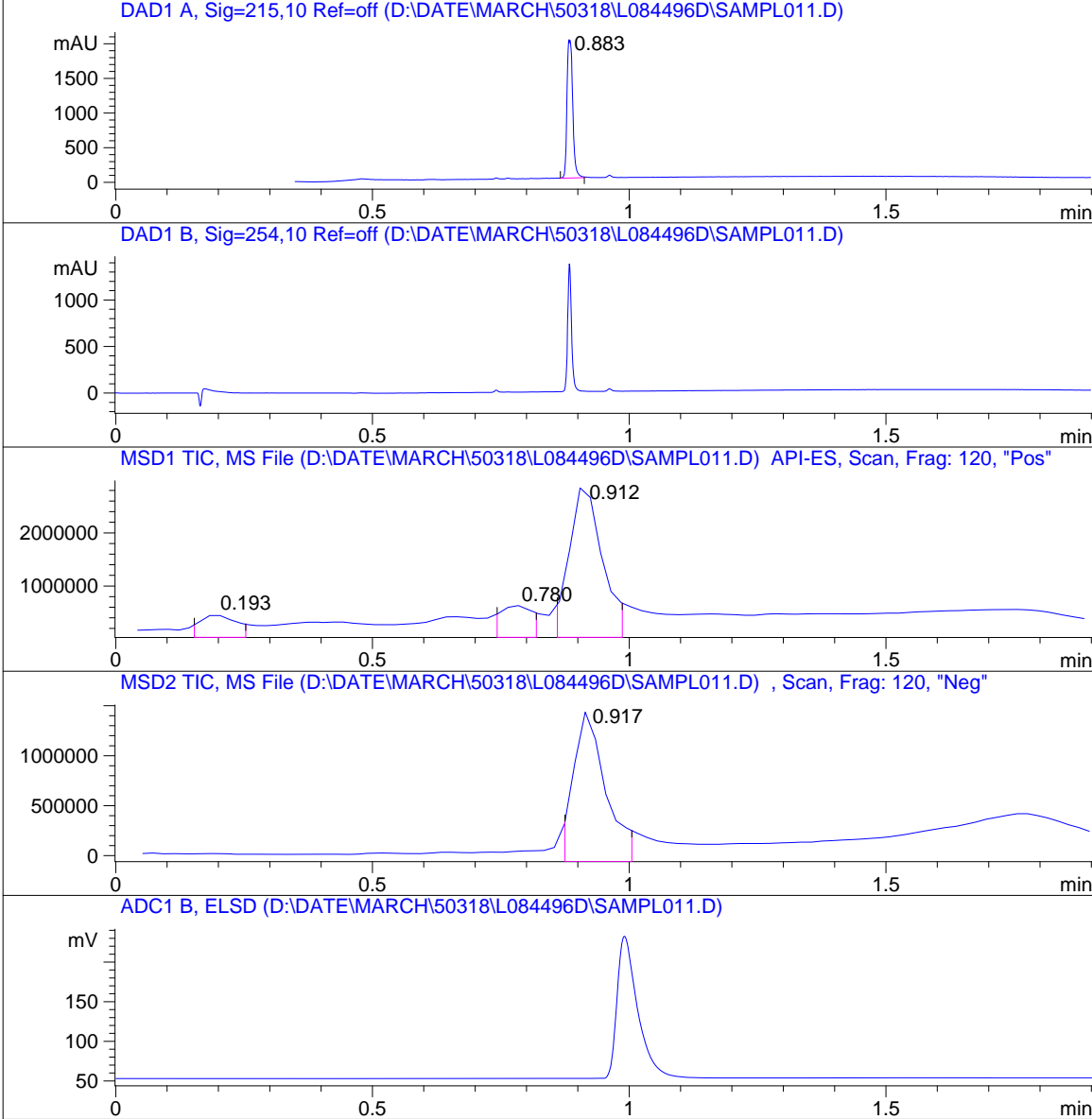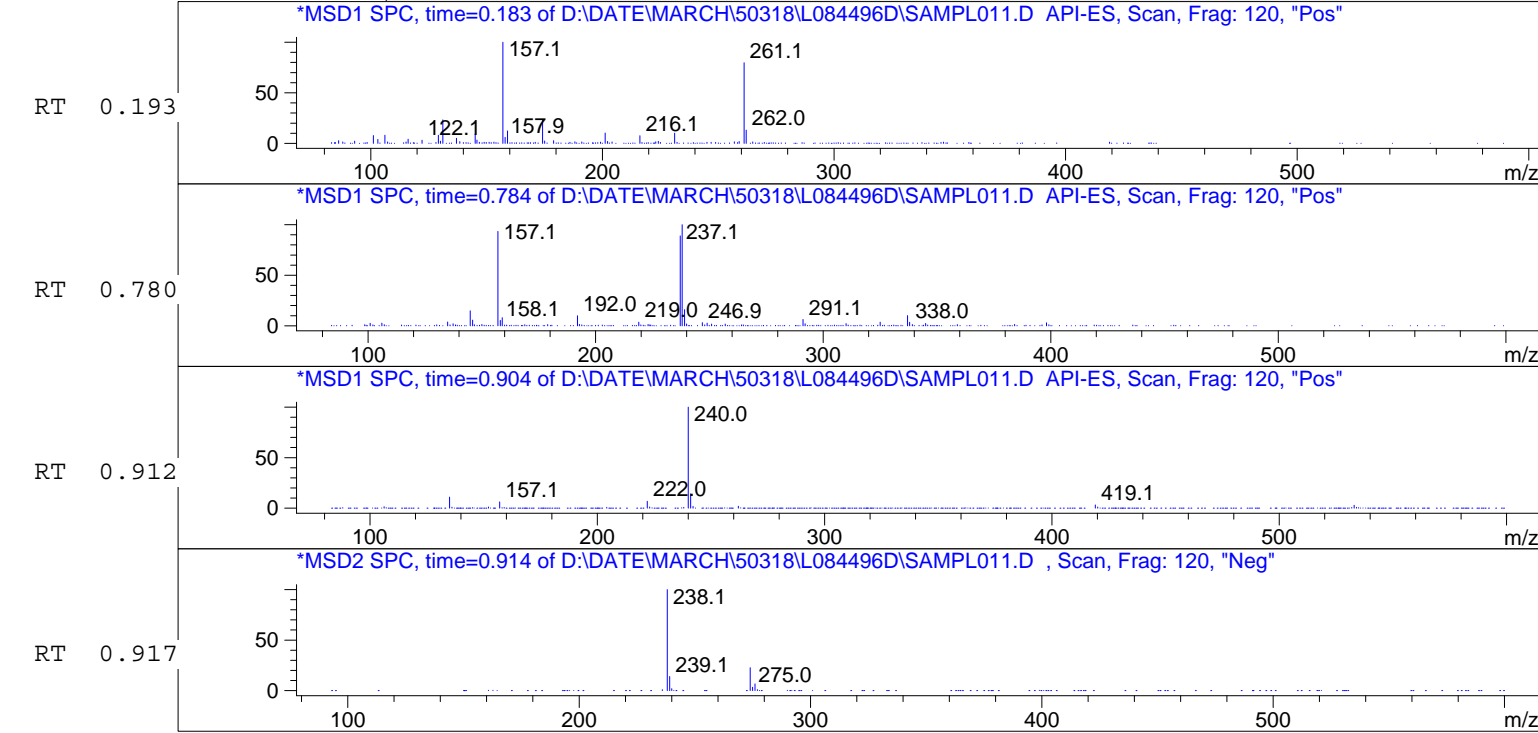

Supplement: Supplementary file 2. [file elife-53779-supp2.zip › mt_vls_62_compounds_QC_data/Compound_14_Z1981813878/Z1981813878_21487758.PDF]

L693622\$1

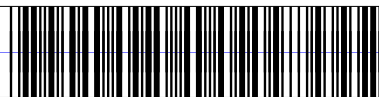

MaxPeak: 96.70%  
Ret\_Time: 0.610 min

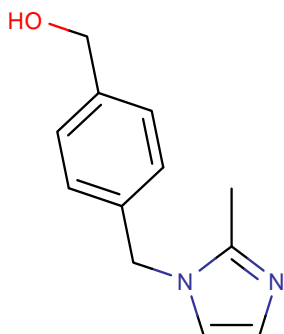

Mol Wt 202.25  
Exact Mass 202.13

| # | Time  | Area% |
|---|-------|-------|
| 1 | 0.610 | 96.70 |
| 2 | 0.791 | 1.98  |
| 3 | 0.893 | 1.32  |

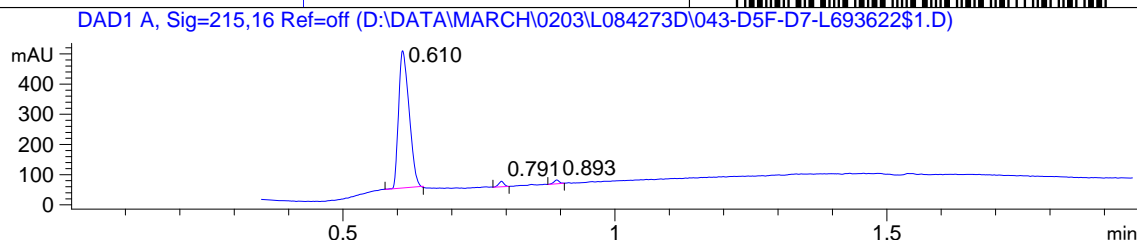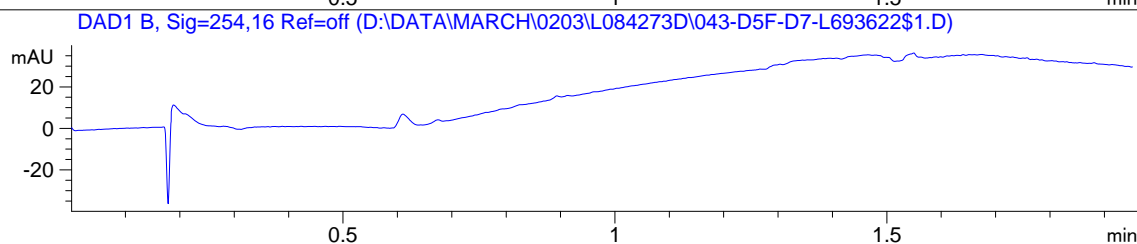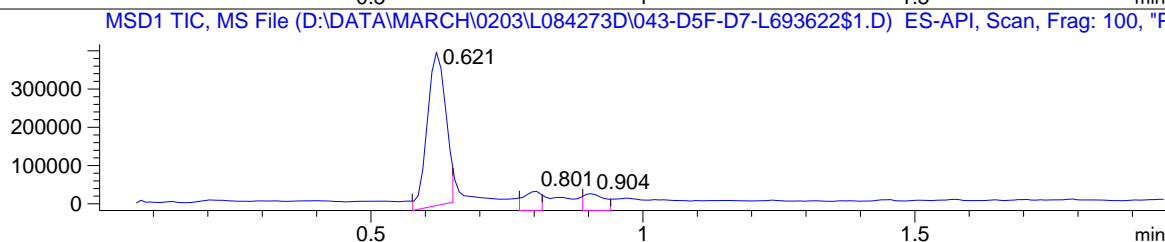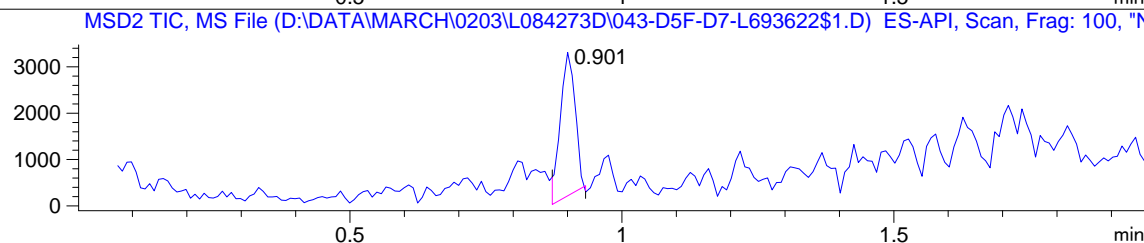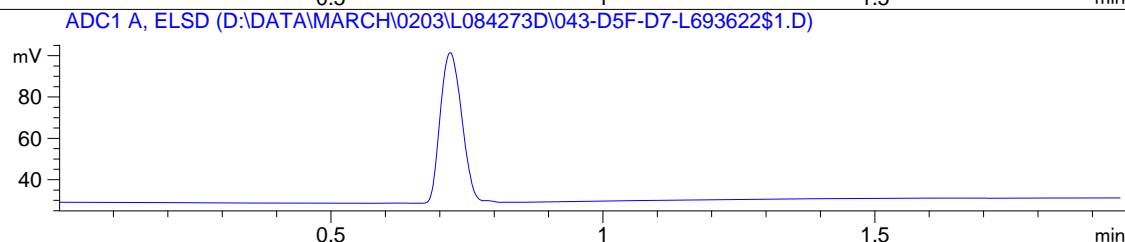

RT 0.621

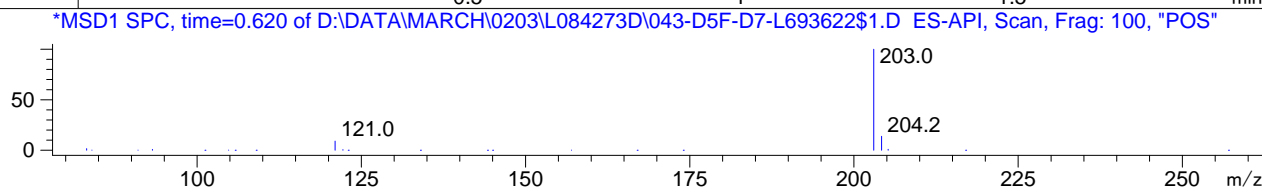

RT 0.801

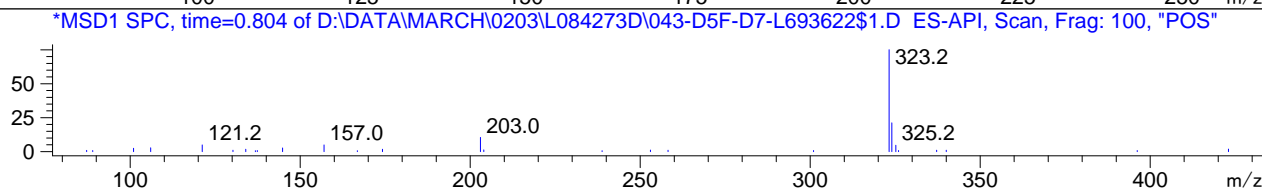

RT 0.904

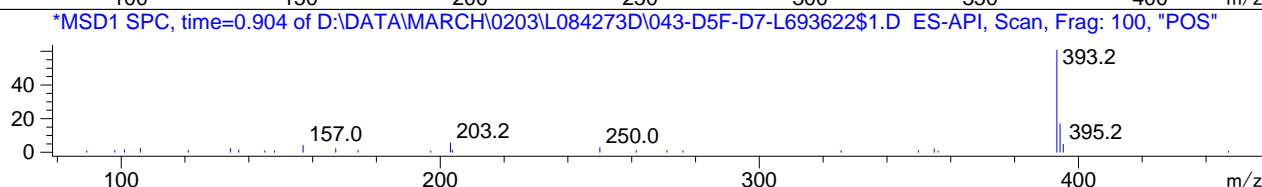

RT 0.901

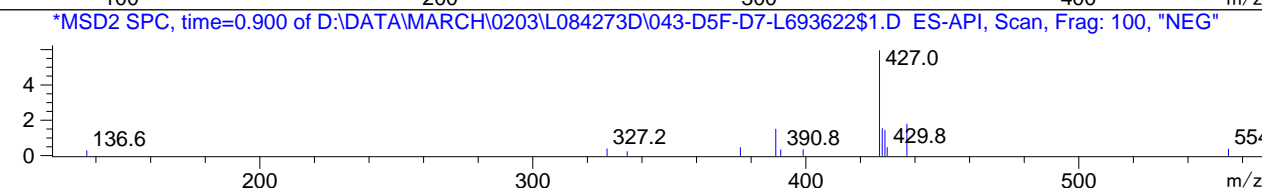

Supplement: Supplementary file 2. [file elife-53779-supp2.zip › mt_vls_62_compounds_QC_data/Compound_15_Z1618072048/Z1618072048_21478901.PDF]

L693637\$2

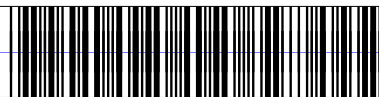

MaxPeak: 100.00%  
Ret\_Time: 0.774 min

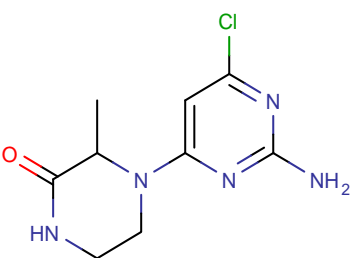

Mol Wt 241.68  
Exact Mass 241.08

| # | Time  | Area%  |
|---|-------|--------|
| 1 | 0.774 | 100.00 |

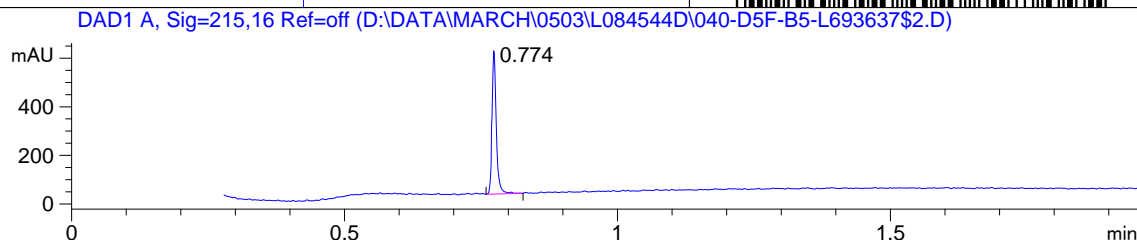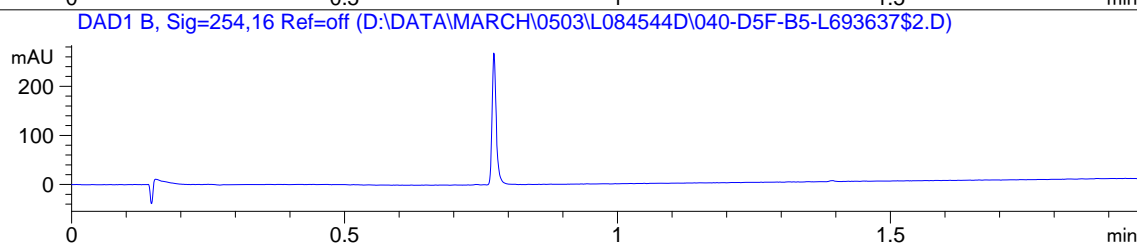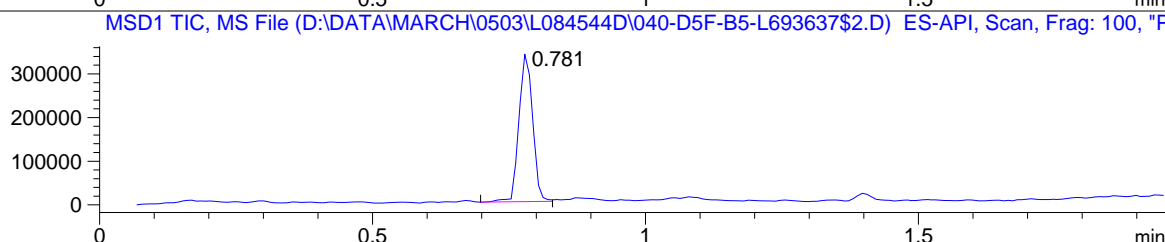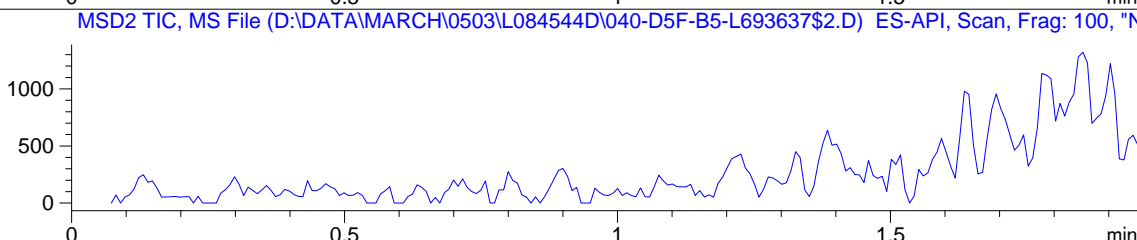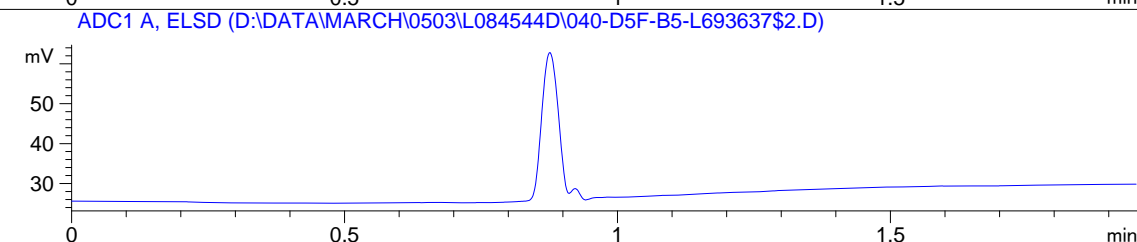

RT 0.781

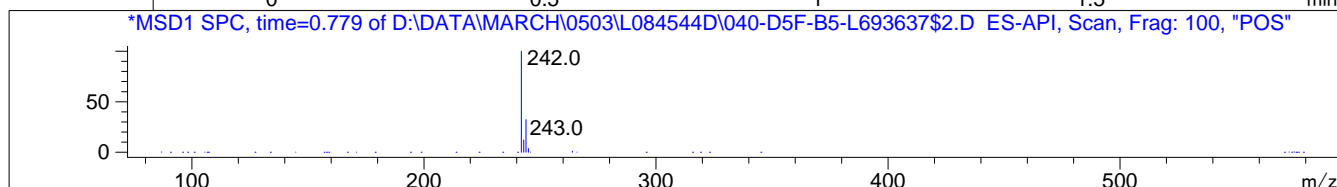

Supplement: Supplementary file 2. [file elife-53779-supp2.zip › mt_vls_62_compounds_QC_data/Compound_20_Z1343343581/Z1343343581_21507576.PDF]

MaxPeak: 98.91%  
Ret\_Time: 0.602 min

L693629\$4

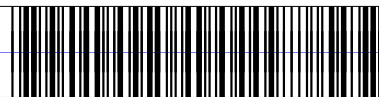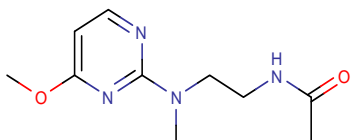

Mol Wt 224.26  
Exact Mass 224.14

| # | Time  | Area% |
|---|-------|-------|
| 1 | 0.602 | 98.91 |
| 2 | 0.945 | 1.09  |

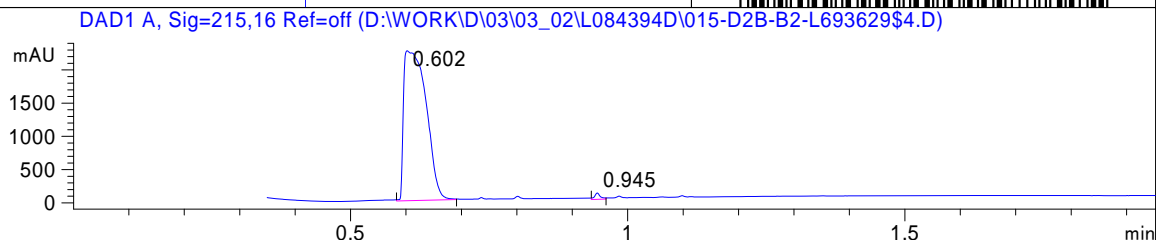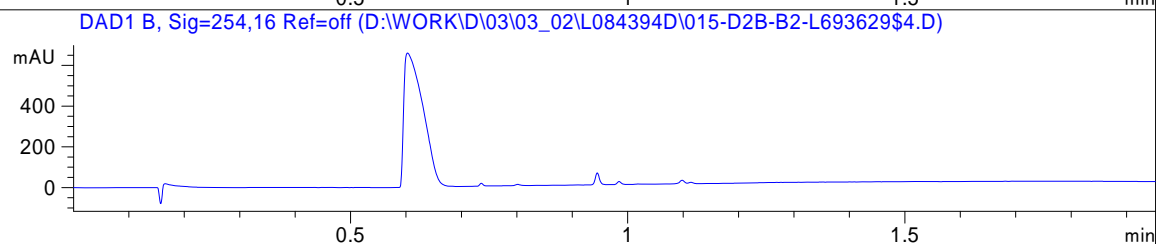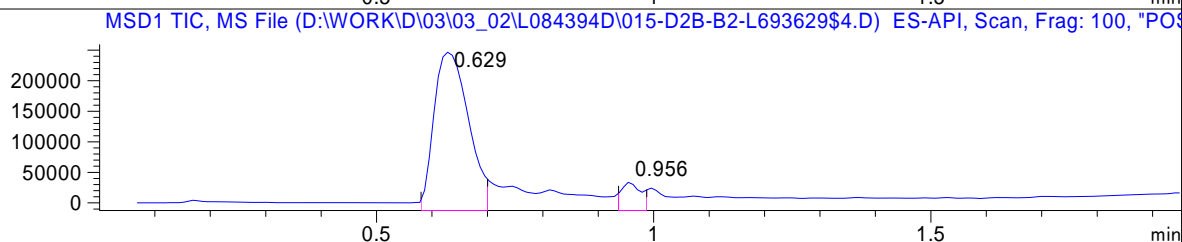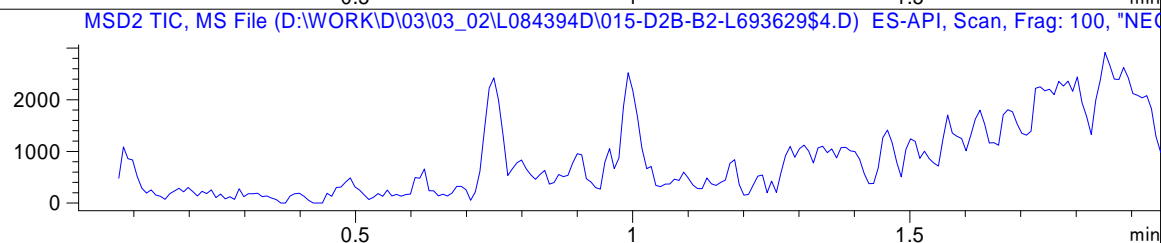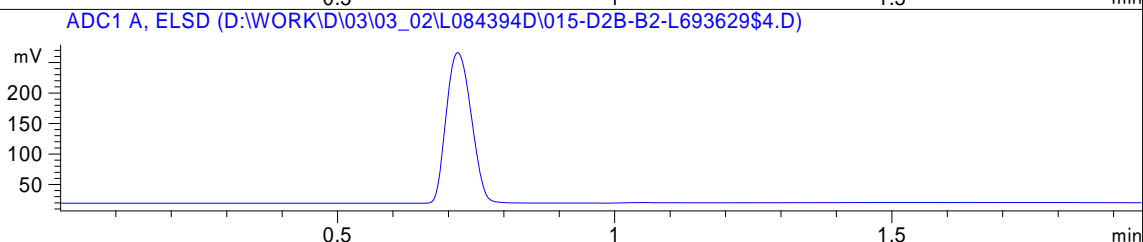

RT 0.629

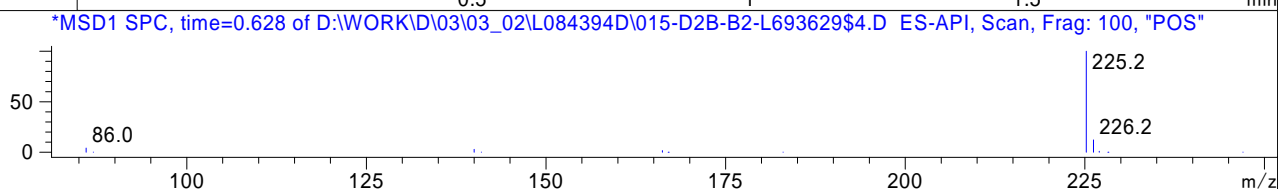

RT 0.956

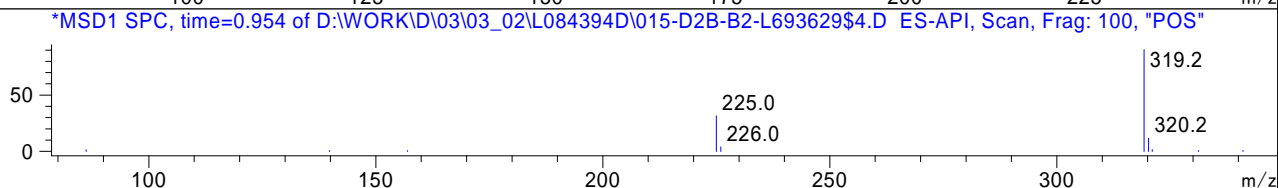

Supplement: Supplementary file 2. [file elife-53779-supp2.zip › mt_vls_62_compounds_QC_data/Compound_21_Z2434357410/Z2434357410_21482025.PDF]

MaxPeak: 96.10%  
Ret\_Time: 0.636 min

L693628\$5

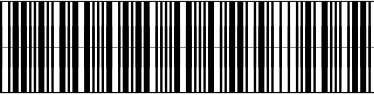

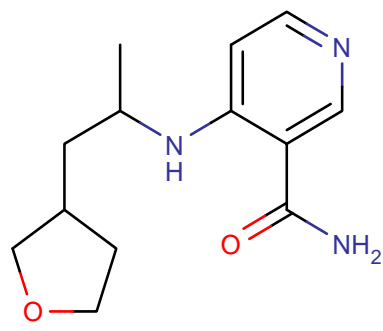

Mol Wt 249.31  
Exact Mass 249.17

| # | Time  | Area% |
|---|-------|-------|
| 1 | 0.636 | 96.10 |
| 2 | 0.738 | 2.41  |
| 3 | 0.962 | 1.49  |

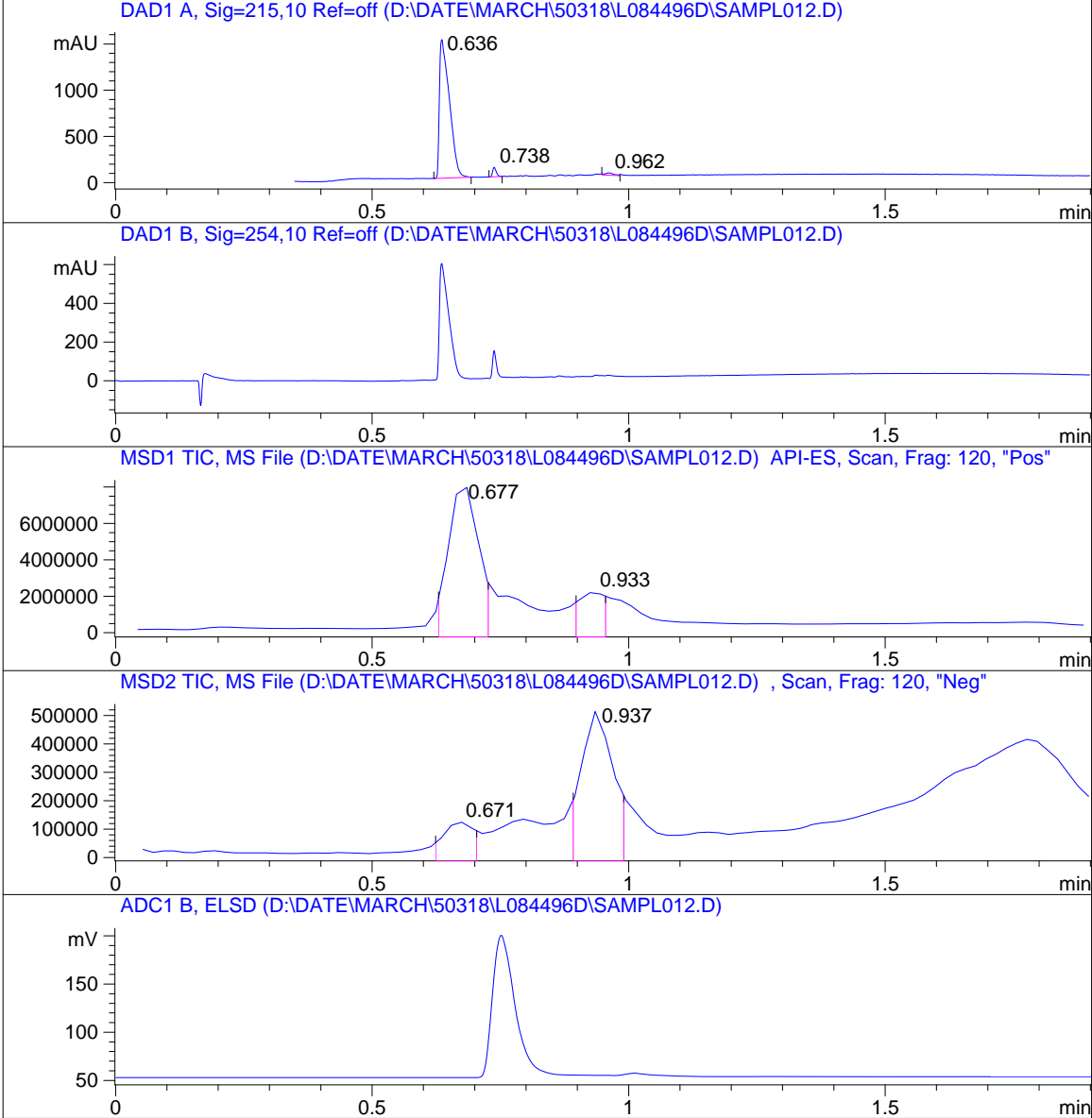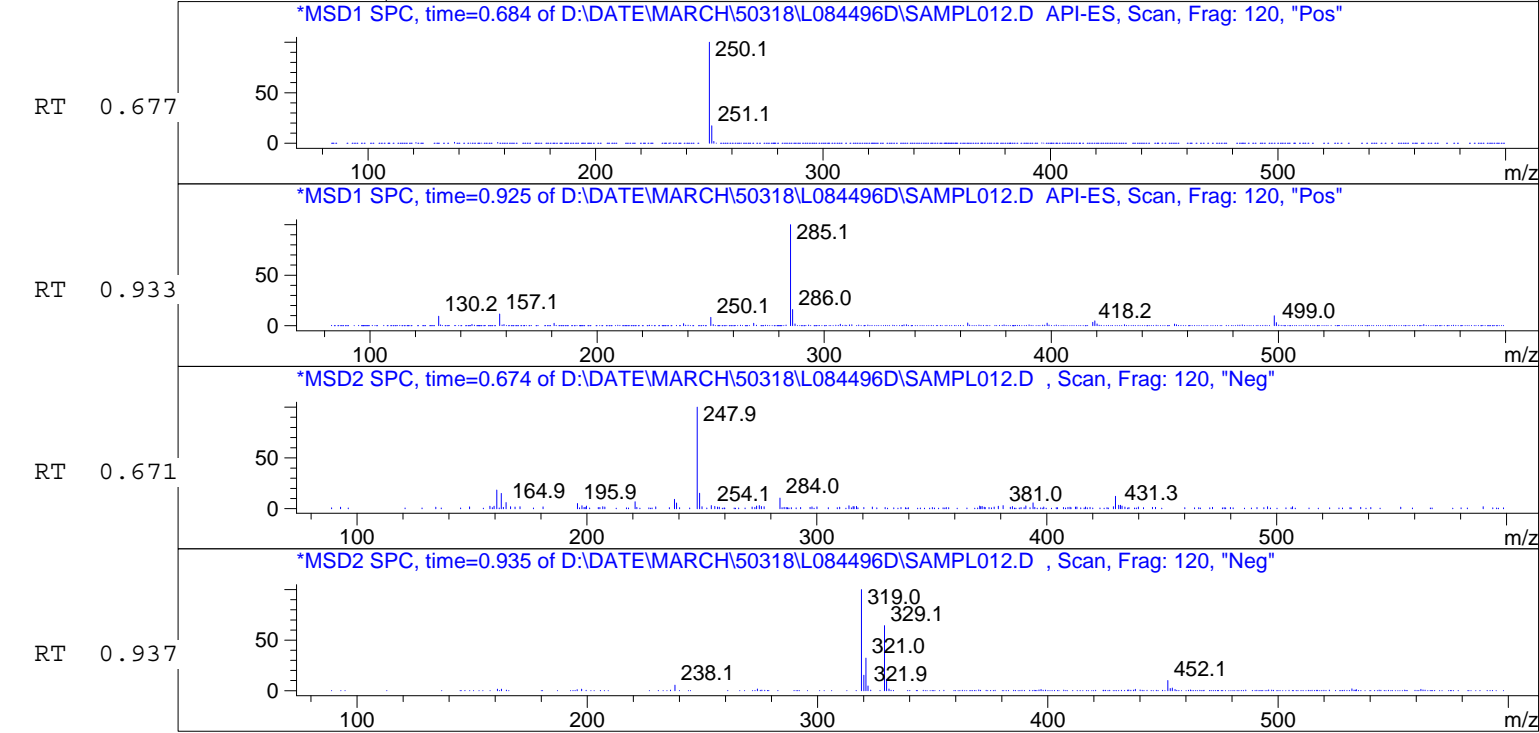

Supplement: Supplementary file 2. [file elife-53779-supp2.zip › mt_vls_62_compounds_QC_data/Compound_26_Z2179535626/Z2179535626_21487759.PDF]

MaxPeak: 100.00%  
Ret\_Time: 0.966 min

L693647\$1

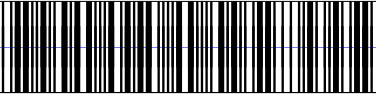

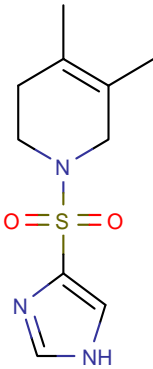

Mol Wt 241.31  
Exact Mass 241.1

| # | Time  | Area%  |
|---|-------|--------|
| 1 | 0.966 | 100.00 |

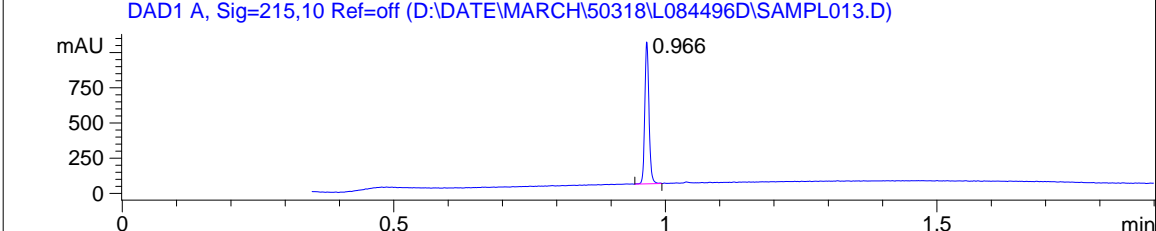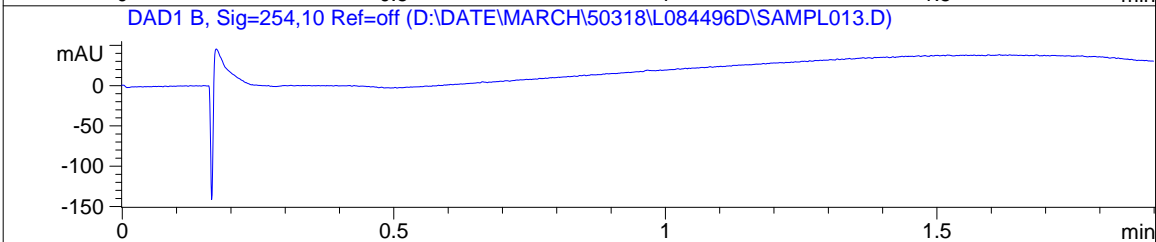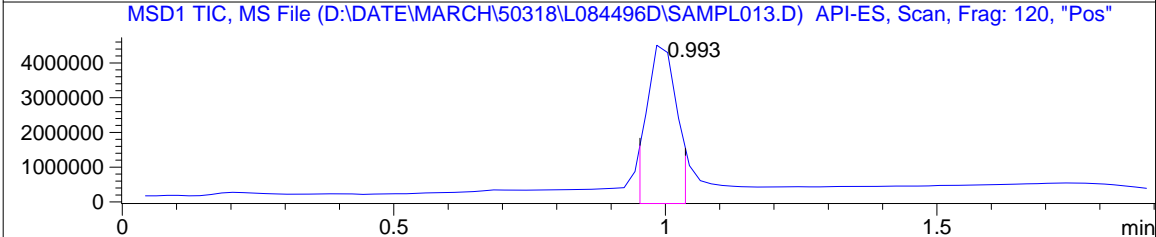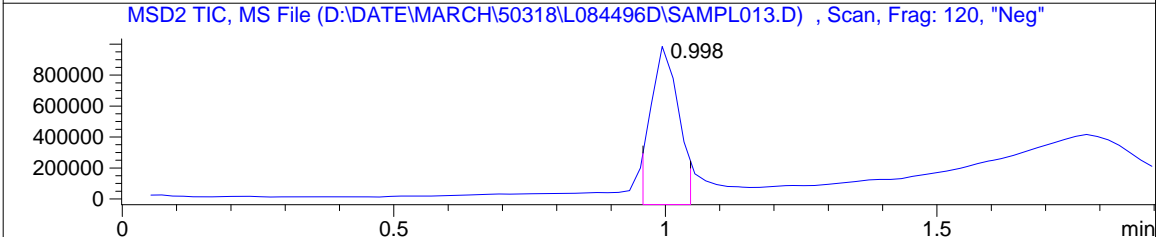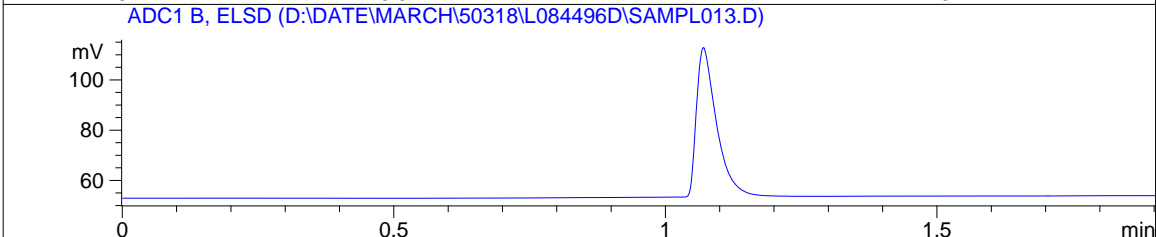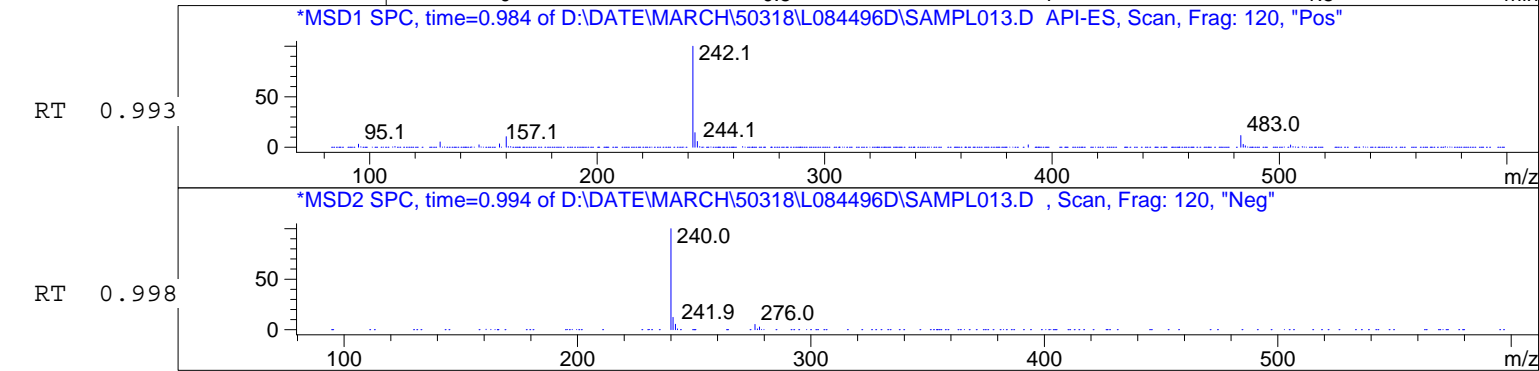

Supplement: Supplementary file 2. [file elife-53779-supp2.zip › mt_vls_62_compounds_QC_data/Compound_30_Z1959154430/Z1959154430_21487760.PDF]

# L693613\$3

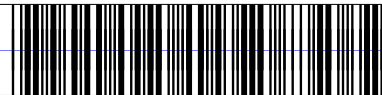

MaxPeak: 100.00%  
Ret\_Time: 0.818 min

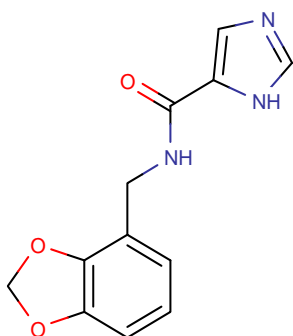

Mol Wt **245.23**  
Exact Mass **245.08**

| # | Time  | Area%  |
|---|-------|--------|
| 1 | 0.818 | 100.00 |

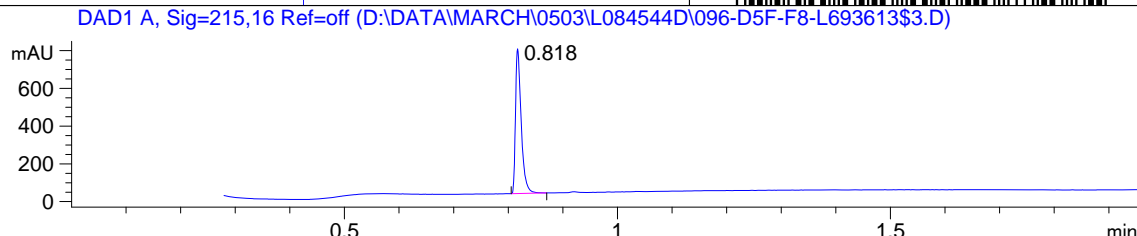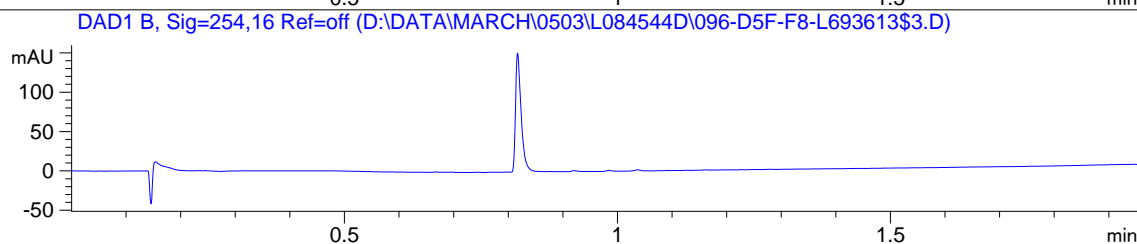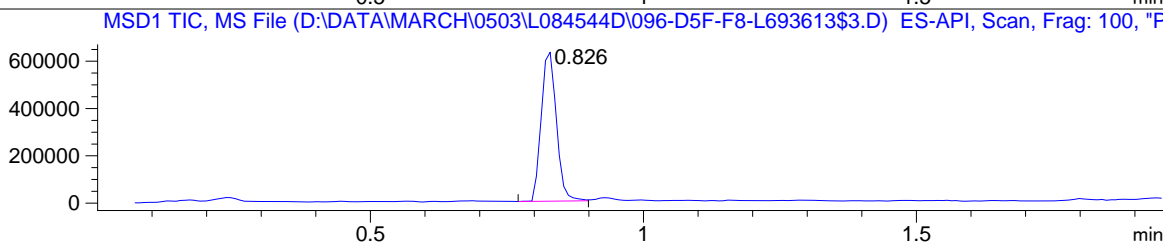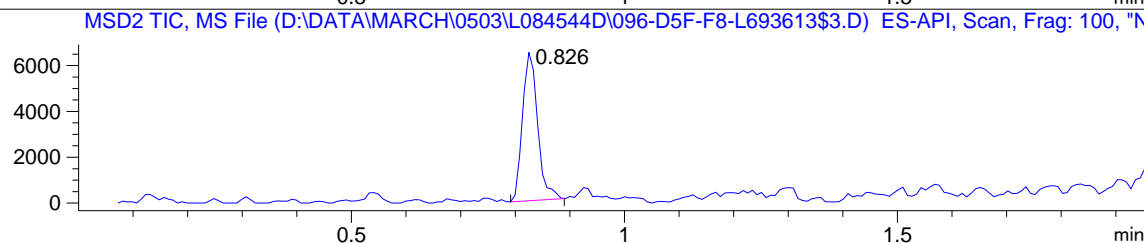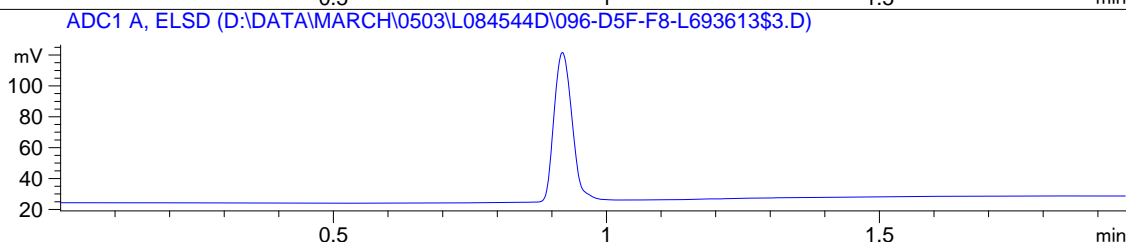

RT 0.826

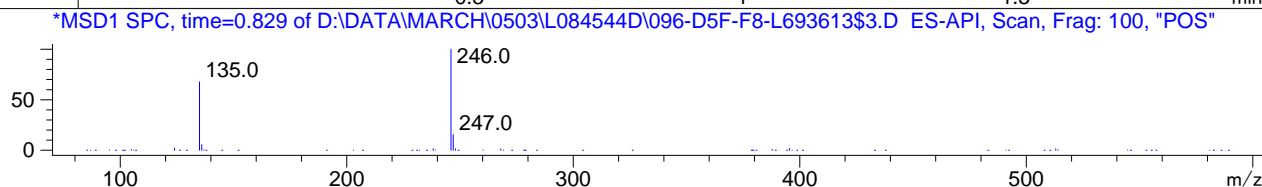

RT 0.826

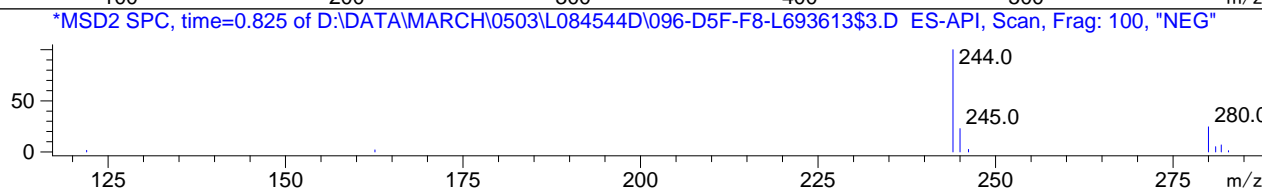

Supplement: Supplementary file 2. [file elife-53779-supp2.zip › mt_vls_62_compounds_QC_data/Compound_33_Z1005838414/Z1005838414_21507615.PDF]

MaxPeak: 98.43%  
Ret\_Time: 1.150 min

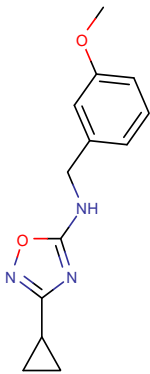

Mol Wt 245.28  
Exact Mass 245.13

| # | Time  | Area% |
|---|-------|-------|
| 1 | 0.591 | 1.57  |
| 2 | 1.150 | 98.43 |

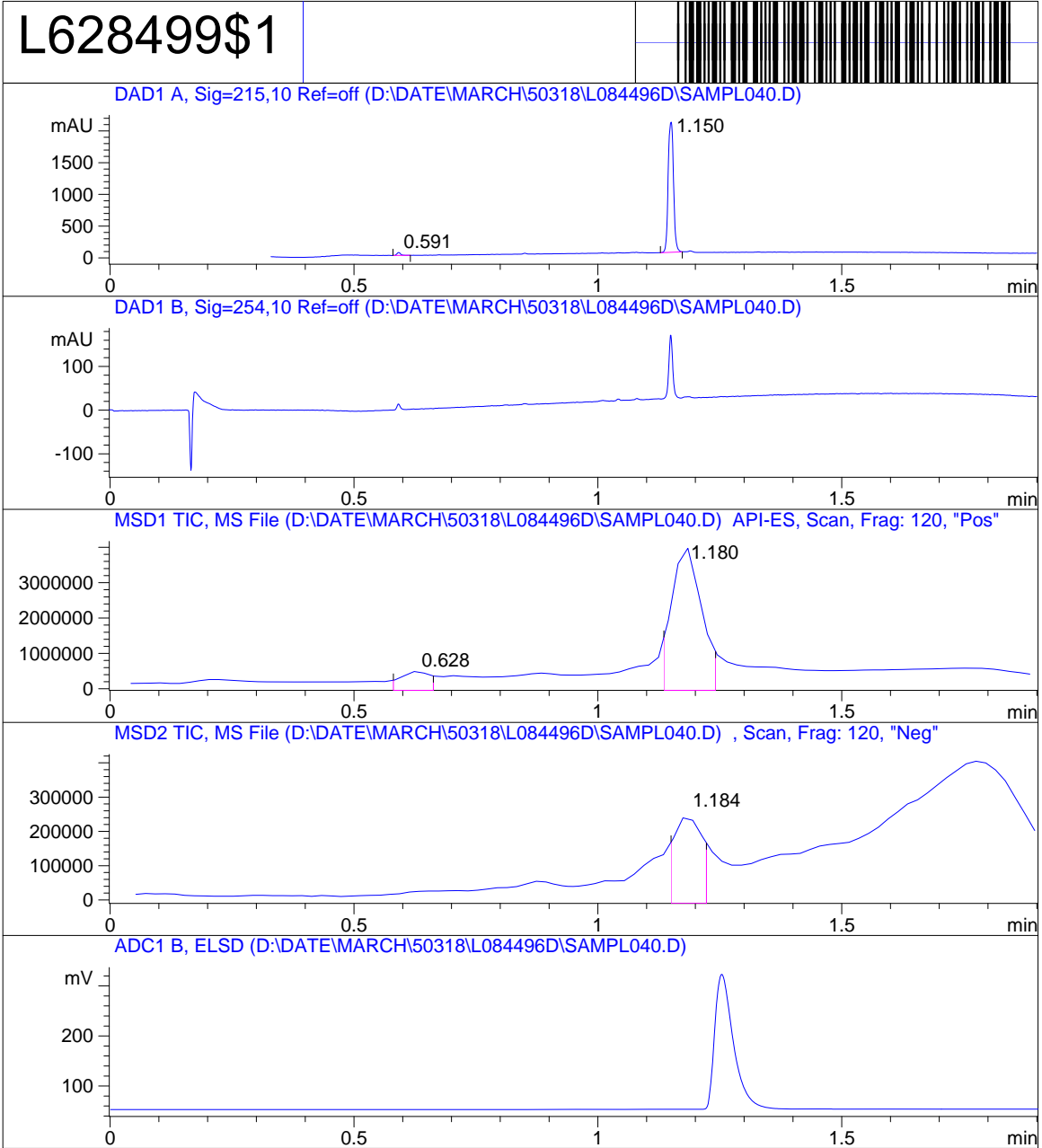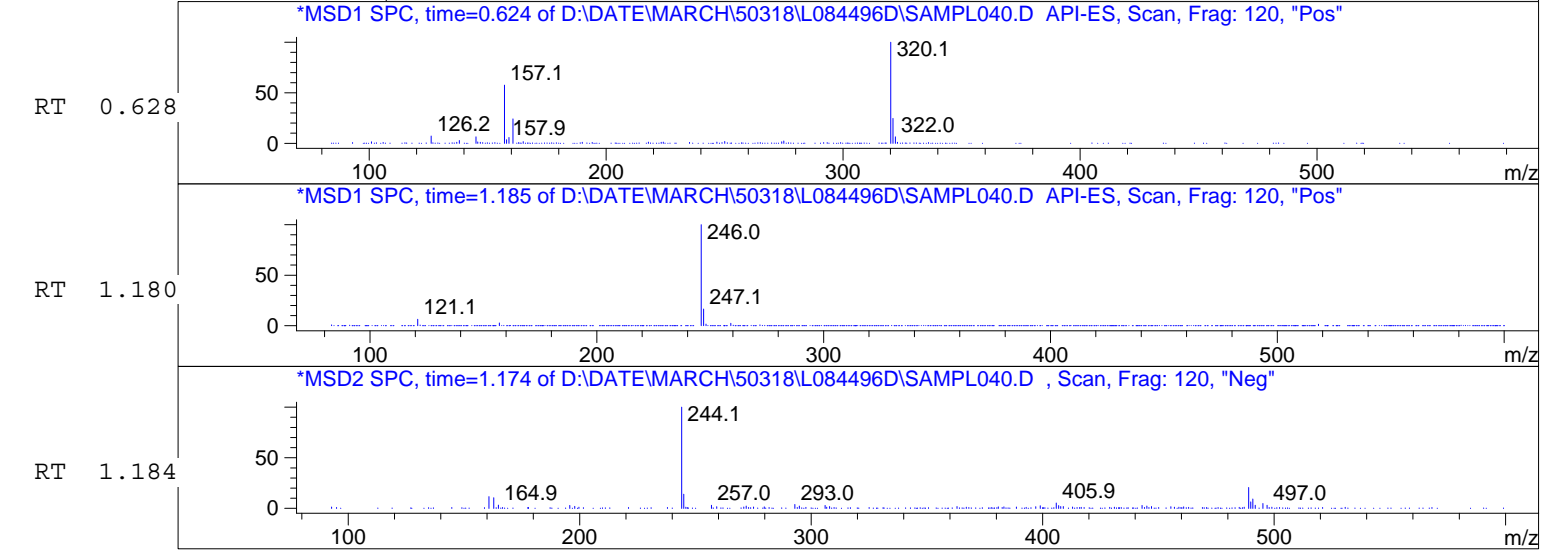

Supplement: Supplementary file 2. [file elife-53779-supp2.zip › mt_vls_62_compounds_QC_data/Compound_37_Z1230796252/Z1230796252_21487786.PDF]

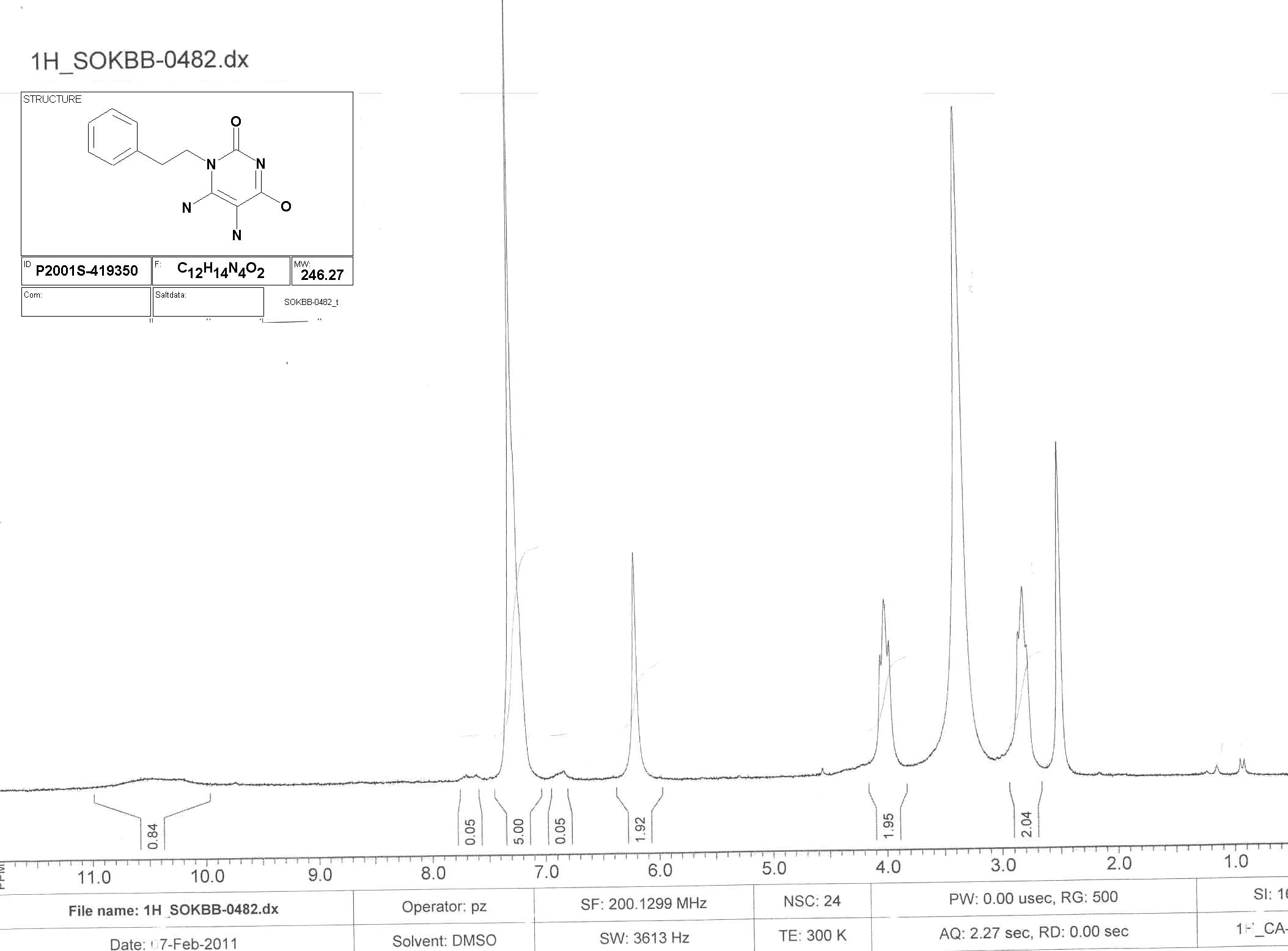

Supplement: Supplementary file 2. [file elife-53779-supp2.zip › mt_vls_62_compounds_QC_data/Compound_38_STL159214.jpg]

Bruker DRX-500, SF=500.13 MHz, 05-09-2019 Base: BBB7074-7

IVY71515 in DMSO-d6/CCl4

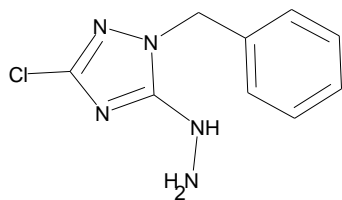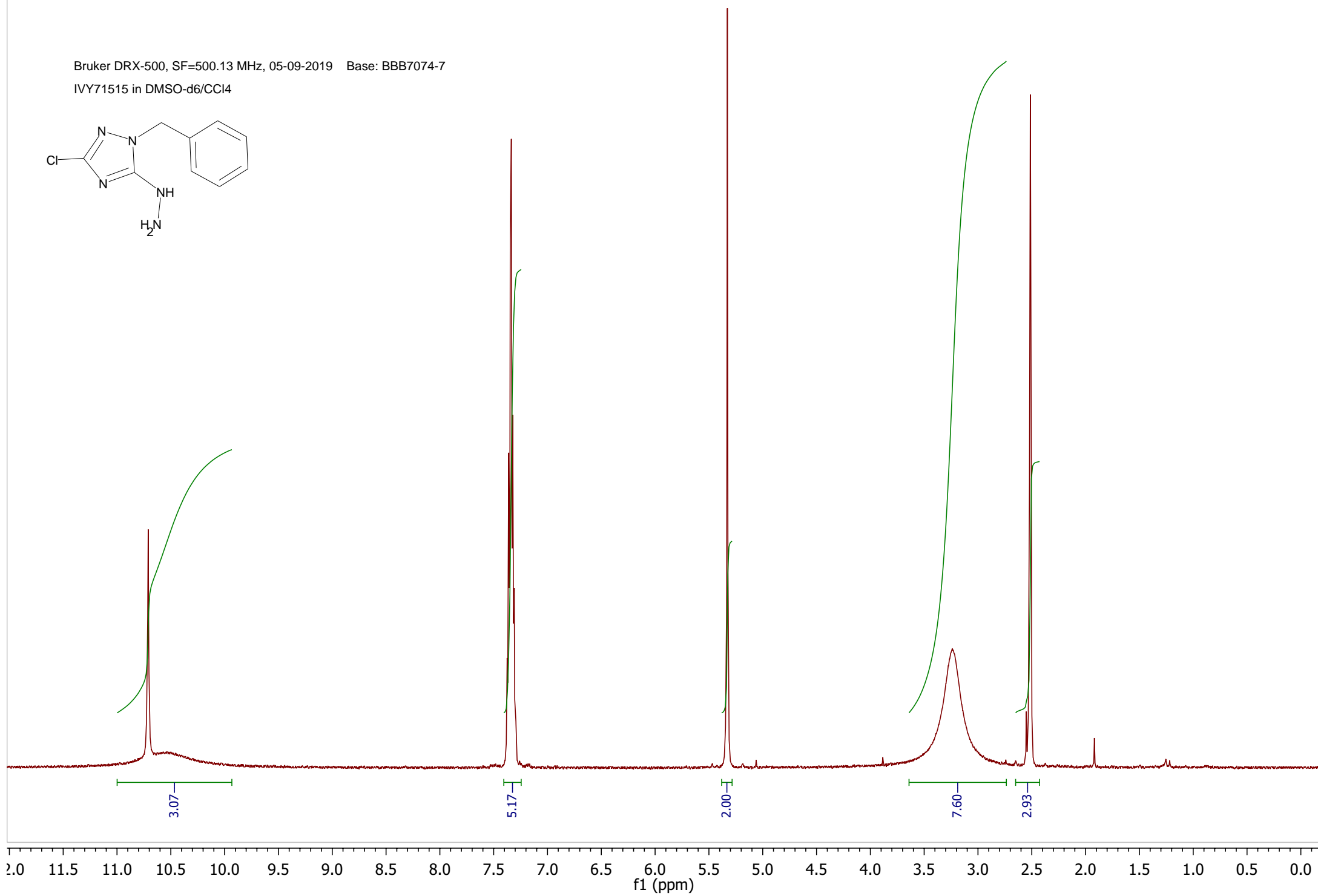

Supplement: Supplementary file 2. [file elife-53779-supp2.zip › mt_vls_62_compounds_QC_data/Compound_39_STL152525.PDF]

L693642\$15

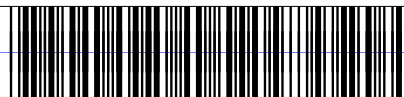

MaxPeak: 91.53%  
Ret\_Time: 1.091 min

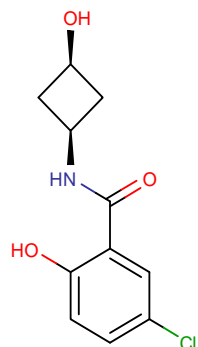

Mol Wt 241.67  
Exact Mass 241.06

| # | Time  | Area% |
|---|-------|-------|
| 1 | 0.737 | 4.52  |
| 2 | 0.867 | 1.48  |
| 3 | 0.937 | 2.47  |
| 4 | 1.091 | 91.53 |

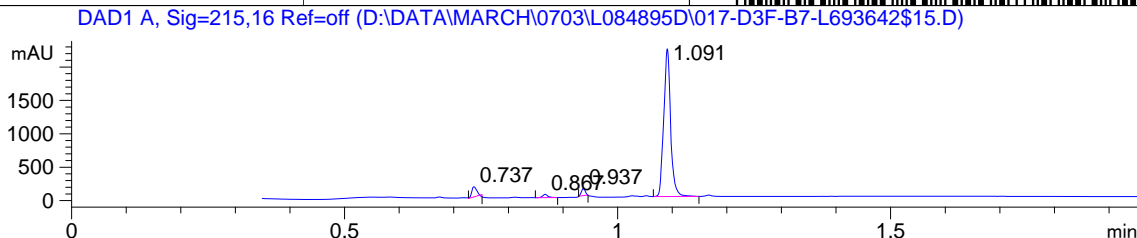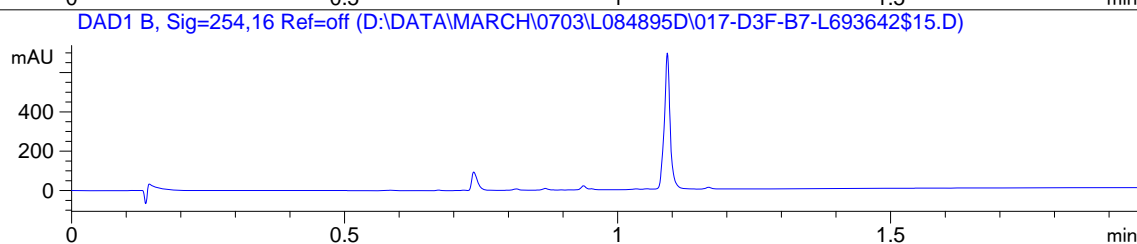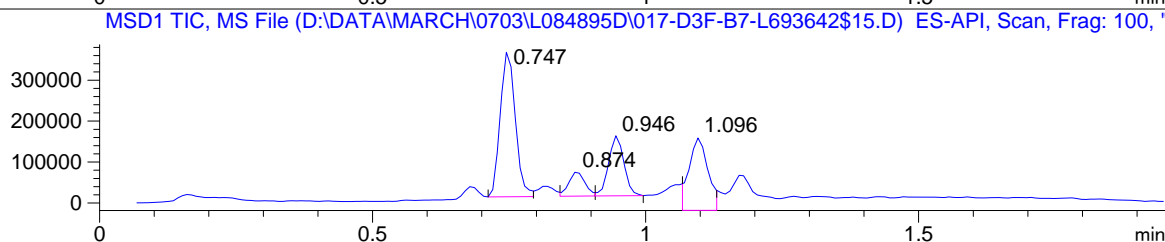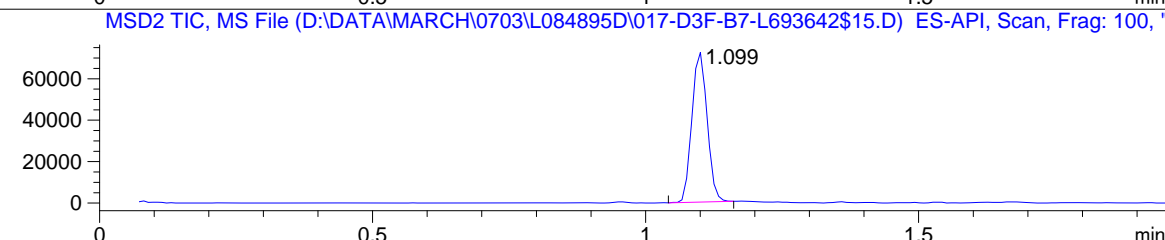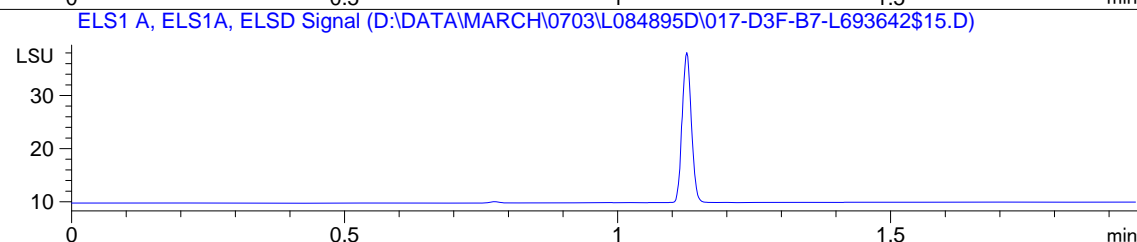

RT 0.747

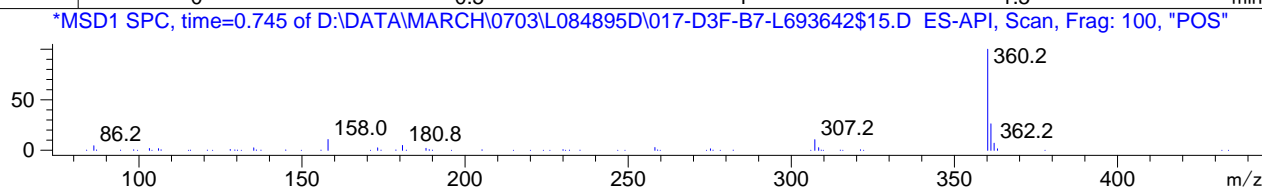

RT 0.874

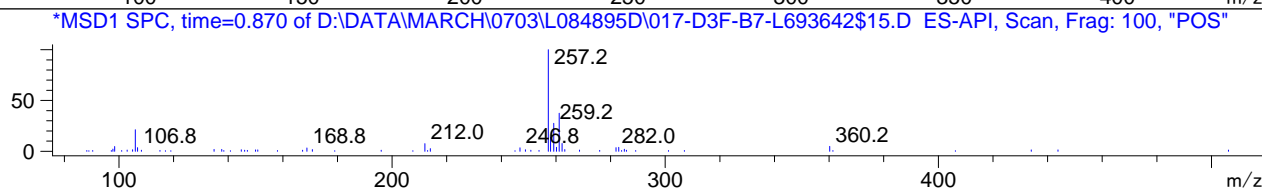

RT 0.946

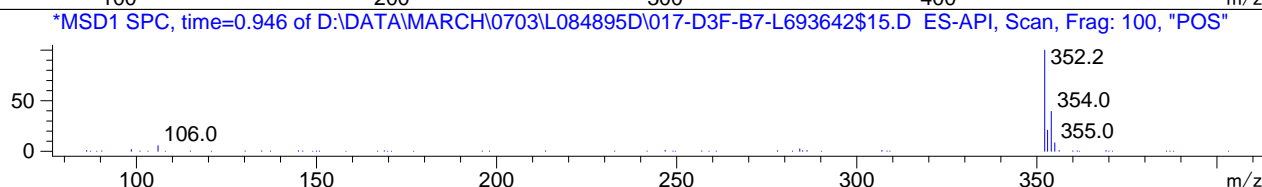

RT 1.096

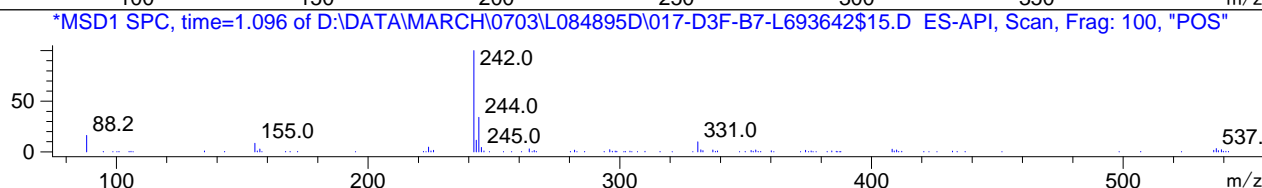

RT 1.099

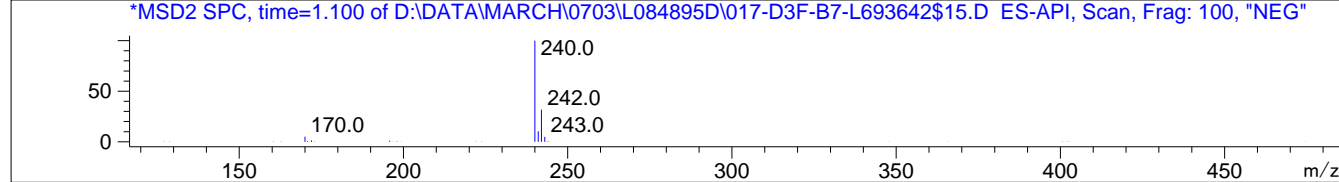

Supplement: Supplementary file 2. [file elife-53779-supp2.zip › mt_vls_62_compounds_QC_data/Compound_4_Z2333538374/Z2333538374_21523252.PDF]

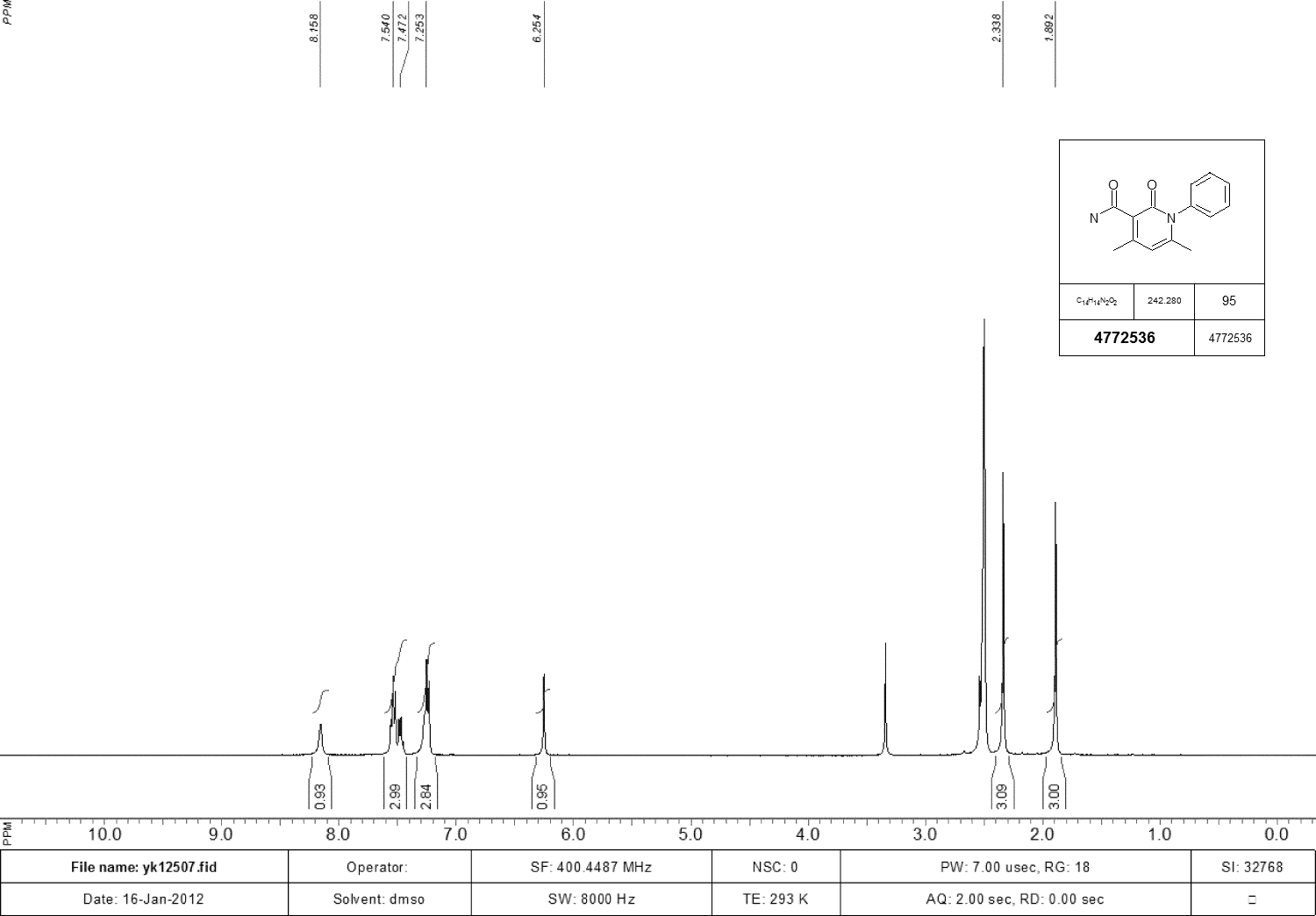

Supplement: Supplementary file 2. [file elife-53779-supp2.zip › mt_vls_62_compounds_QC_data/Compound_40_otava_1/4772536.tif]

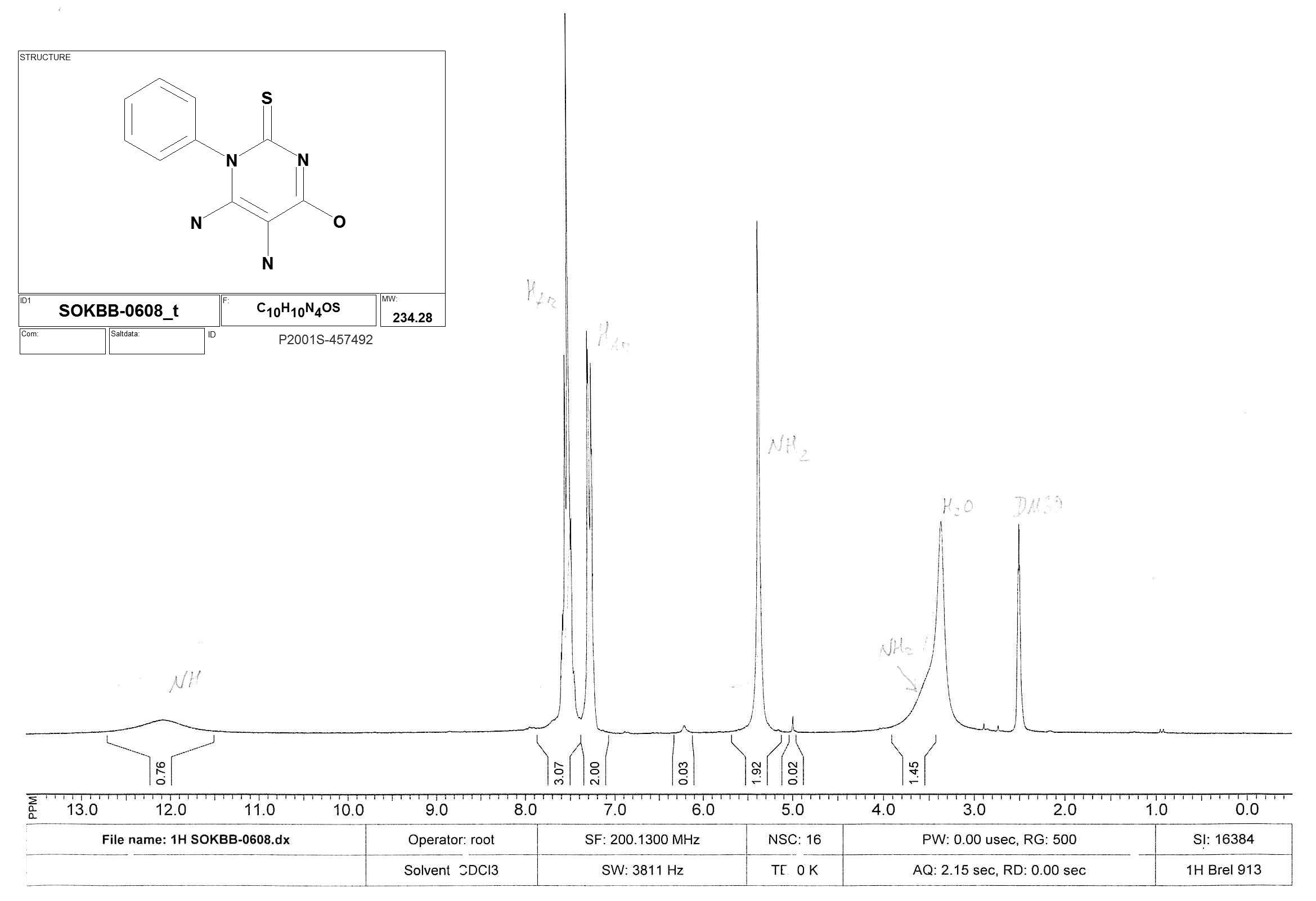

Supplement: Supplementary file 2. [file elife-53779-supp2.zip › mt_vls_62_compounds_QC_data/Compound_41_STL245617.jpg]

Bruker DRX-500, SF=500.13 MHz, 05-09-2019 Base: BBB7074-7

SDR49199 in DMSO-d6/CCl4

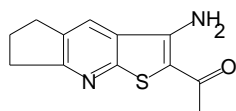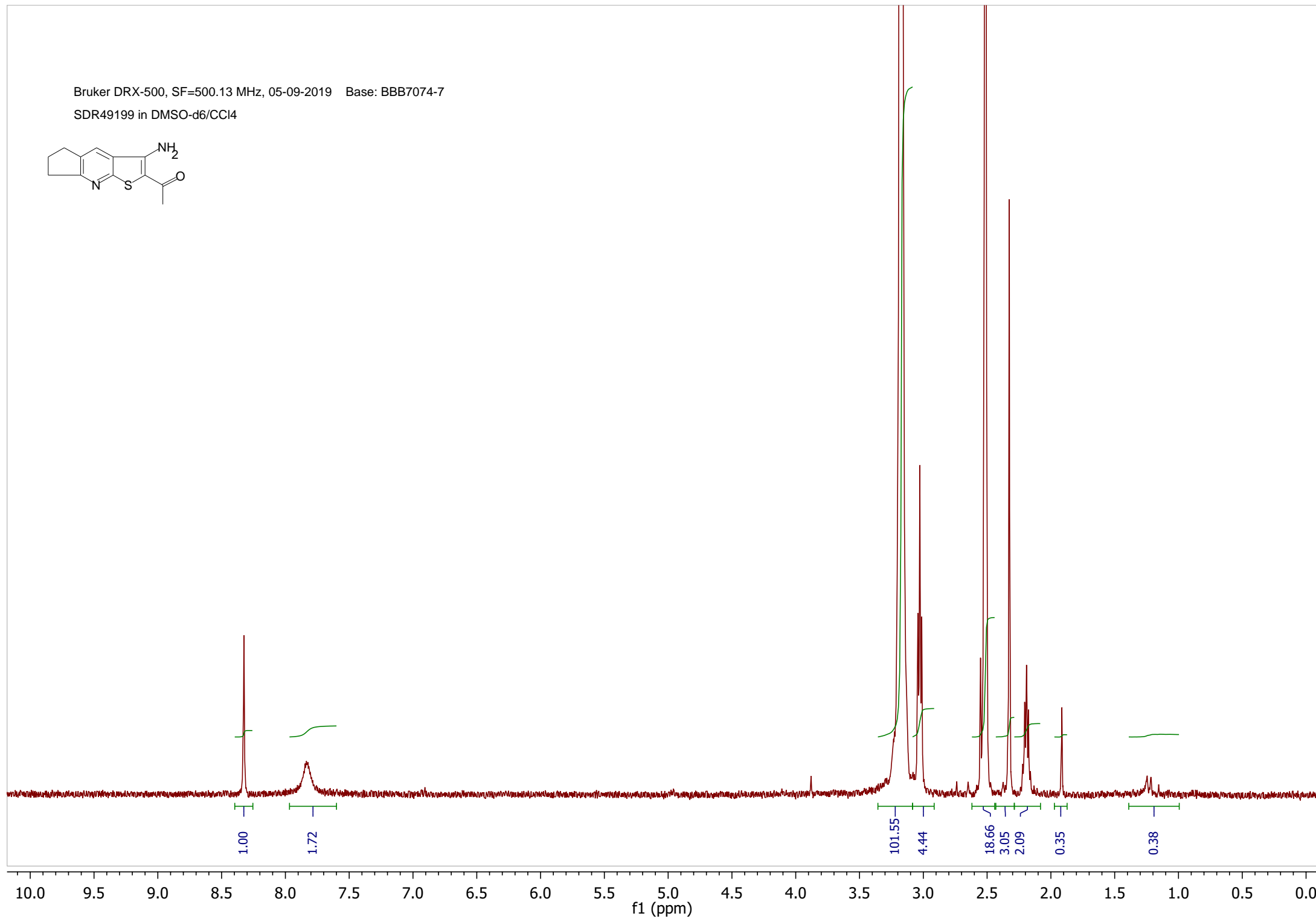

Supplement: Supplementary file 2. [file elife-53779-supp2.zip › mt_vls_62_compounds_QC_data/Compound_45_STK677082.PDF]

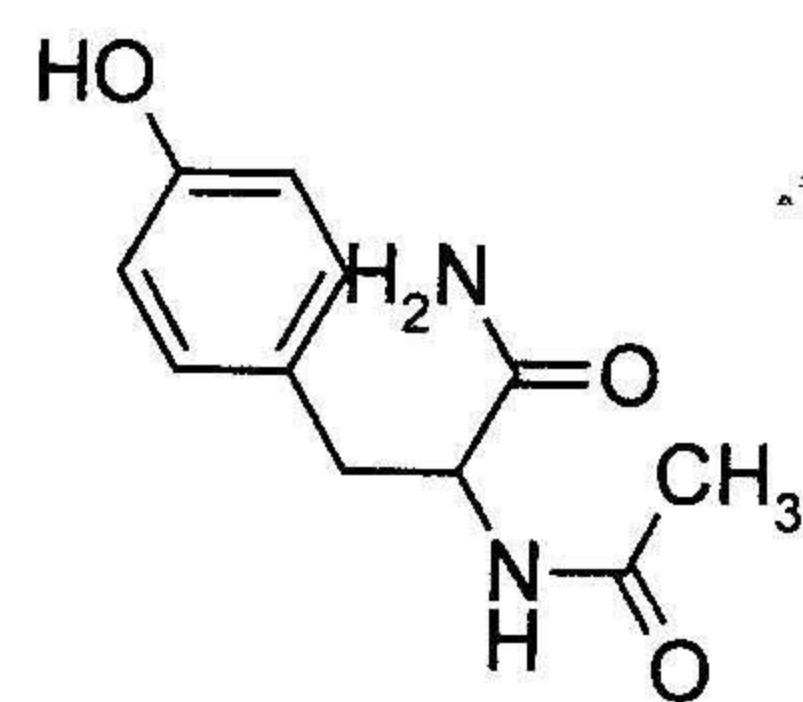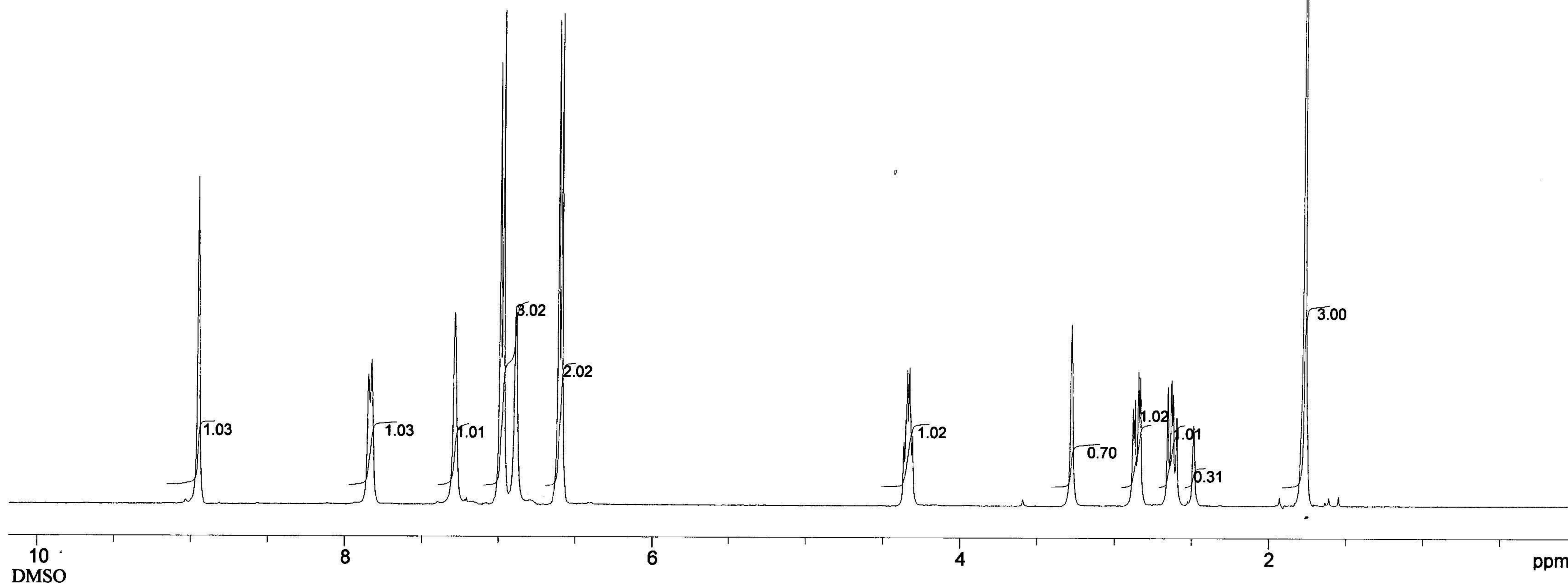

Supplement: Supplementary file 2. [file elife-53779-supp2.zip › mt_vls_62_compounds_QC_data/Compound_46_Alinda_1/C46_IBS-L0126050.pdf]

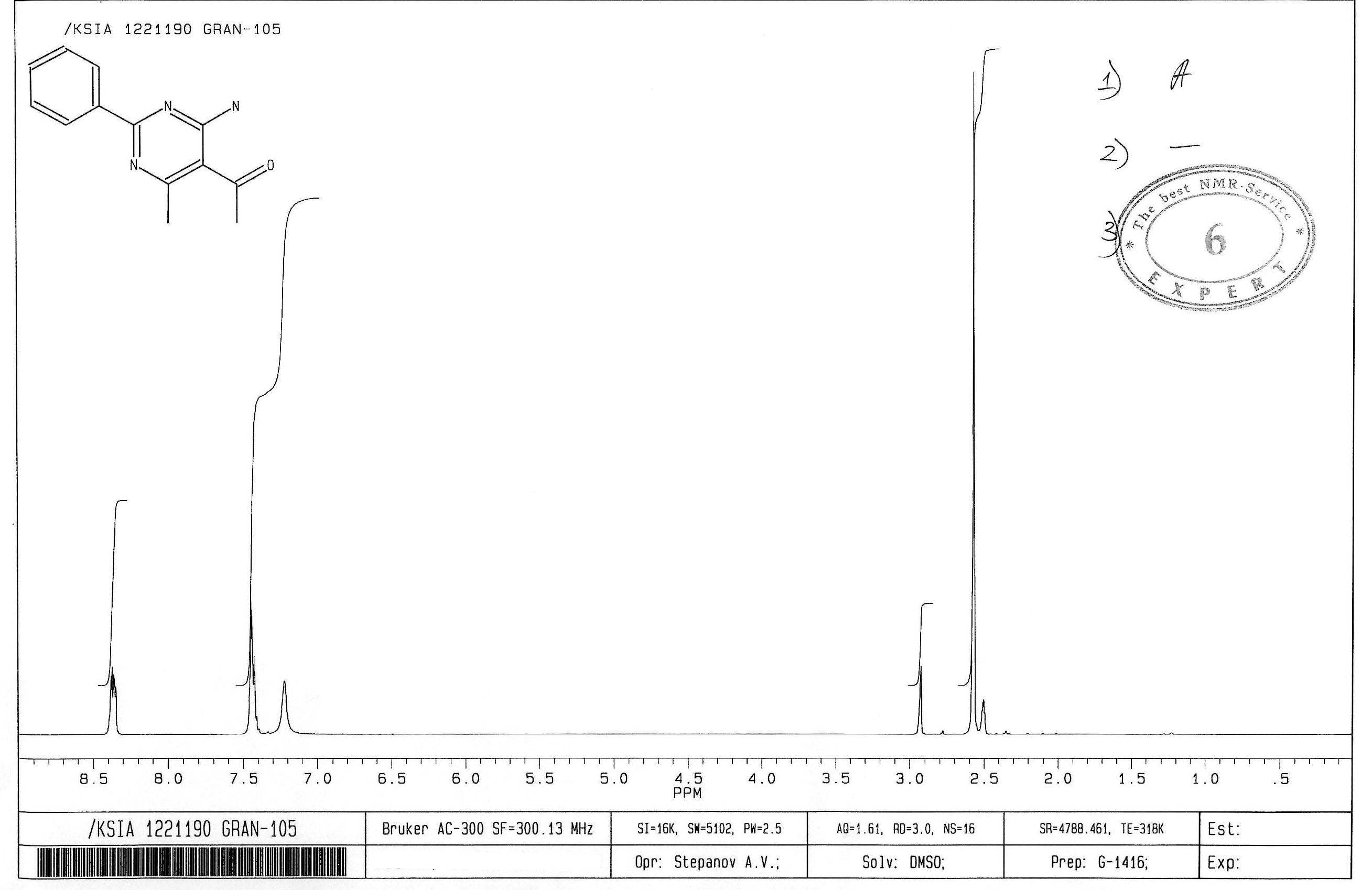

Supplement: Supplementary file 2. [file elife-53779-supp2.zip › mt_vls_62_compounds_QC_data/Compound_48_STL298248.jpg]

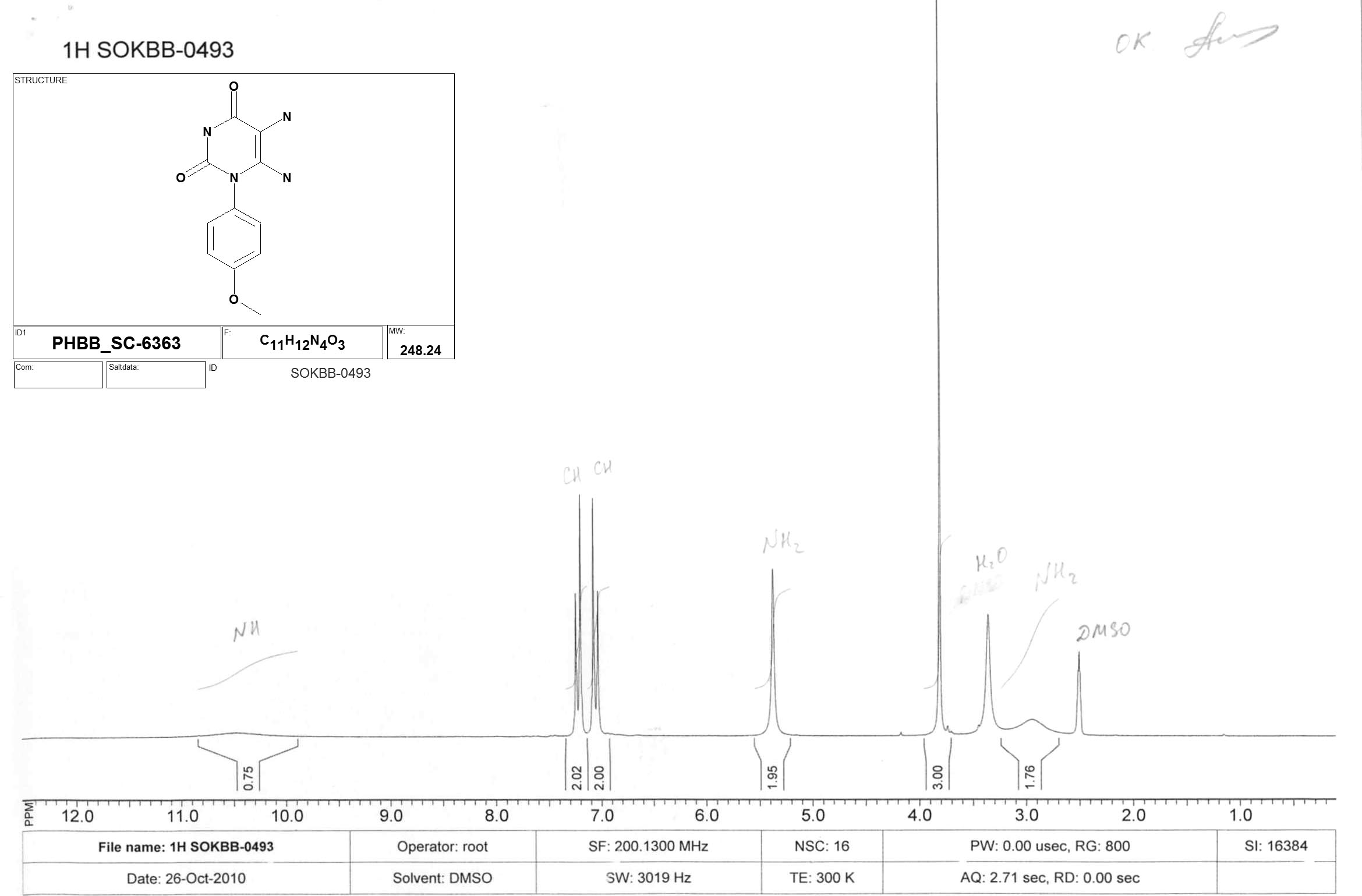

Supplement: Supplementary file 2. [file elife-53779-supp2.zip › mt_vls_62_compounds_QC_data/Compound_49_STL146798.jpg]

Bruker DRX-500, SF=500.13 MHz, 05-09-2019 Base: BBB7074-7

SDR57038 in DMSO-d6/CCl4

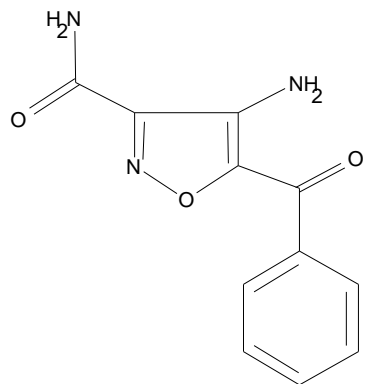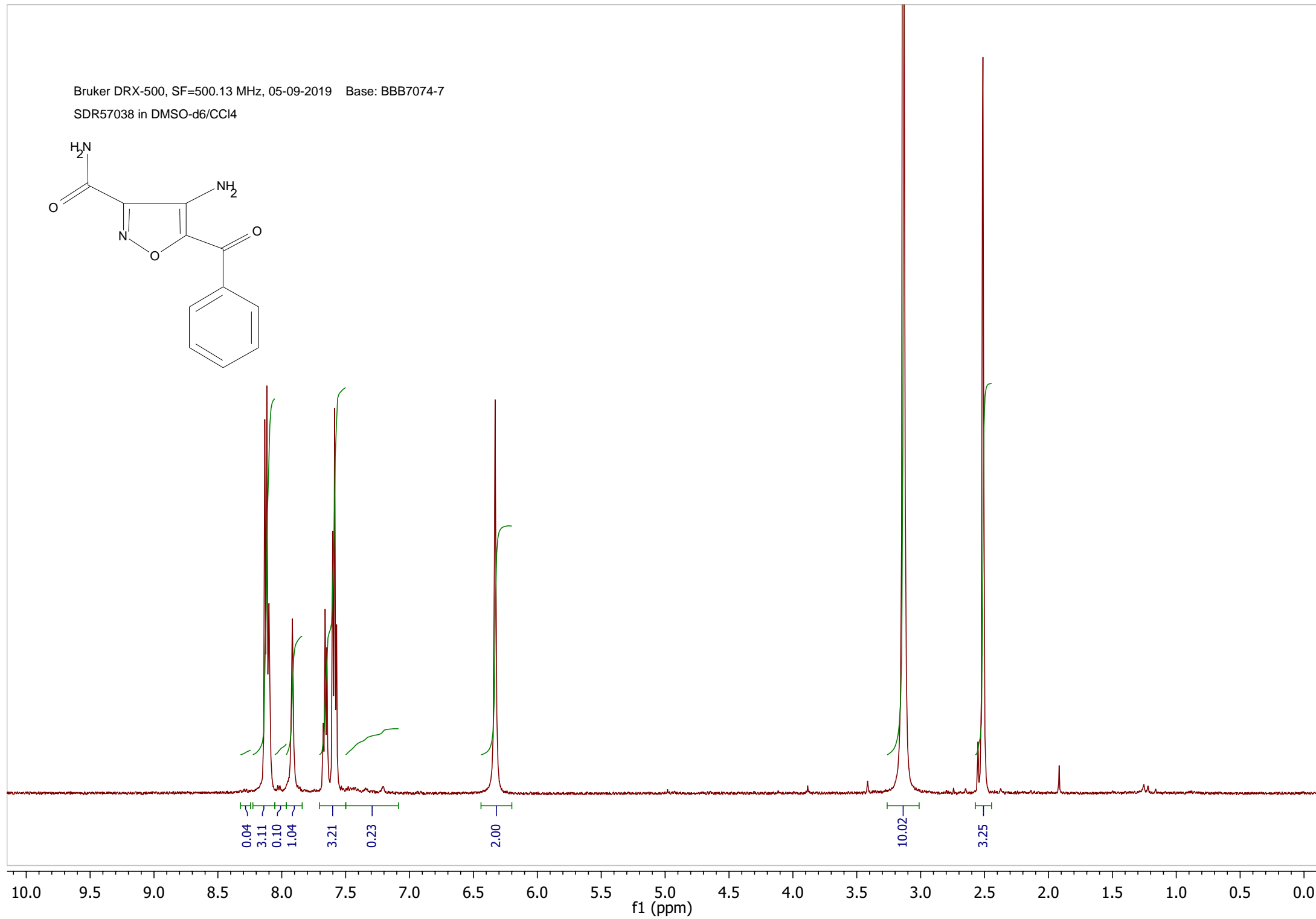

Supplement: Supplementary file 2. [file elife-53779-supp2.zip › mt_vls_62_compounds_QC_data/Compound_52_STK767382.PDF]

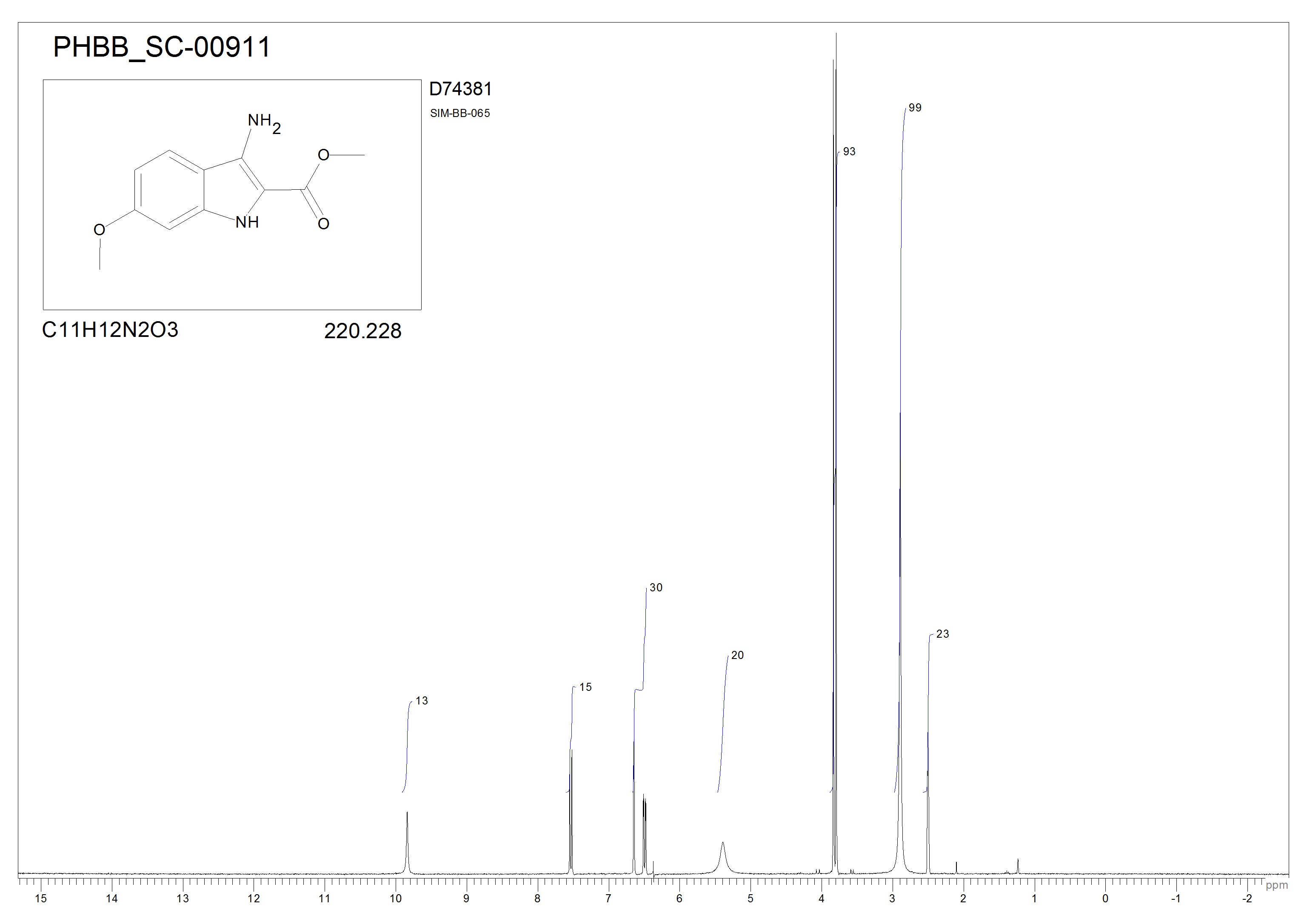

Supplement: Supplementary file 2. [file elife-53779-supp2.zip › mt_vls_62_compounds_QC_data/Compound_54_STK504679.jpg]

Bruker DRX-500, SF=500.13 MHz, 05-09-2019 Base: BBB7074-7

SDS01718 in DMSO-d<sub>6</sub>/CCl<sub>4</sub>

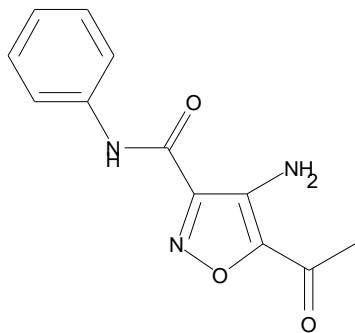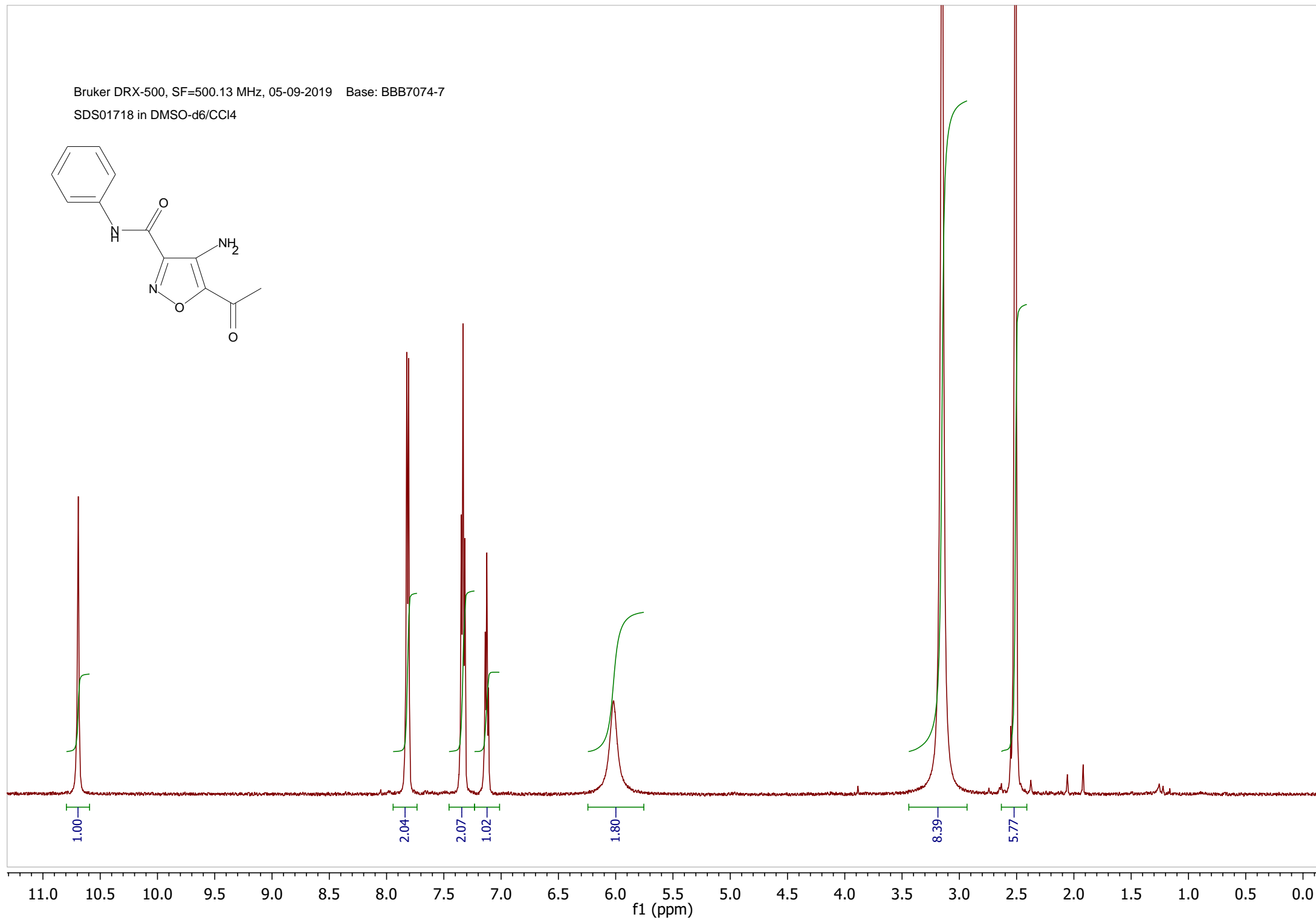

Supplement: Supplementary file 2. [file elife-53779-supp2.zip › mt_vls_62_compounds_QC_data/Compound_56_STK784239.PDF]
