# Supplementary material for: Structure-based discovery of potent and selective melatonin receptor agonists
Source: eLife. 2020 Mar 2;9:e53779. doi: 10.7554/eLife.53779 (PMC7080406; doi:10.7554/eLife.53779)
Supplement: Supplementary file 2. [file elife-53779-supp2.zip › mt_vls_62_compounds_QC_data/Compound_46_Alinda_1/Certificate_IBS-L0126050.doc]

**ALINDA CHEMICAL TRADE COMPANY LIMITED**

ALINDA CHEMICAL TRADE COMPANY LIMITED

Kuskovskaya str,20a, office 409B

Moscow,Russia

**Phone:** +7(495) 989-96-74

**E-mail addresses:** [sid@alinda.ru](mailto:sid@alinda.ru) ; [sale@alinda.ru](mailto:sale@alinda.ru)

**CERTIFICATE OF ANALYSIS**

**Product: alpha-(Acetylamino)-4-hydroxy-benzenepropanamide**

**Synonym:** **N-Acetyltyrosylnamide**

**Catalog Number: IBS-L0126050**

**CAS Number**: 1948-71-6

**MFCD Number:** MFCD00063043

**Physical Appearance: Solid, powder**

**Molecular Weight: 222.25**

**Purity (NMR-H): 90+%**

**Country origin - Russia**

Creation Date: 01.02.2018
