# Supplementary material for: Structure-based discovery of potent and selective melatonin receptor agonists
Source: eLife. 2020 Mar 2;9:e53779. doi: 10.7554/eLife.53779 (PMC7080406; doi:10.7554/eLife.53779)
Supplement: Supplementary file 2. [file elife-53779-supp2.zip › mt_vls_62_compounds_QC_data/Compound_46_Alinda_1/MSDS_IBS-L0126050.doc]

**Alinda Chemical Trade Company Ltd**

**MATERIAL SAFETY DATA SHEET**

****** SECTION 1 - CHEMICAL PRODUCT AND COMPANY IDENTIFICATION ******

**Name:** **alpha-(Acetylamino)-4-hydroxy-benzenepropanamide.**

**Synonym:** **N-Acetyltyrosylnamide**

**Catalog Numbers: IBS-L0126050**

```
Company Identification:
Alinda Chemical Trade Company Ltd
Kuskovskaya str,20a, office 409B
Moscow,Russia
Phone: +7(495) 989-96-74
E-mail addresses:
  sid@alinda.ru
  sale@alinda.ru


**** SECTION 2 - COMPOSITION, INFORMATION ON INGREDIENTS ****
```

| **CAS#** | **Chemical Name** | **%** | **MFCD** | **Haz Symbols** | **Risk Phrases** |
| --- | --- | --- | --- | --- | --- |
| 1948-71-6 | **alpha-(Acetylamino)-4-hydroxy-benzenepropanamide** | 95 | MFCD00063043 | Xi | 36/37/38 |

Formula: C11H14N2O3

```
Molecular Weight: 222.25


**** SECTION 3 - HAZARDS IDENTIFICATION ****

Specific identification of hazards to human and environment:
Irritating to eyes, respiratory system and skin. May be harmful if swallowing.


**** SECTION 4 - FIRST AID MEASURES ****
Inhalation:
If inhaled, remove to fresh air immediately. If breathing is difficult, give oxygen. Get medical aid.
Skin:
In case contact, immediately wash skin with soap and copious amount of water
Eyes:
In case contact, immediately flush eyes with plenty of water for at least 15 minutes. Get medical aid.
Ingestion:
If swallowed, wash mouth with water provided person is conscious. Get medical aid.


**** SECTION 5 - FIRE FIGHTING MEASURES ****

Extinguishing Media:
Suitable: Water spray, carbon dioxide, dry chemical powder.
Special risks
Specific Hazard: Emits toxic fumes under fire conditions.
Special protective equipment for firefighters:
Wear a self-contained breathing apparatus and protective clothing to prevent contact with skin and eyes.


**** SECTION 6 - ACCIDENTAL RELEASE MEASURES ****

Procedure(s) of personal precaution(s)
Wear self-contained breathing apparatus, and heavy rubber gloves.
Methods for cleaning up
Sweep up, place in a bag and hold for waste disposal. Avoid raising dust. Ventilate area and wash site after material pickup in compete.


**** SECTION 7 - HANDLING and STORAGE ****

Handling:
Avoid contact with skin and eyes and inhalation. Ensure good ventilation at the workplace.
Storage:
Keep away from sources of ignition. Store in cool, dry place. Store in a tightly closed container.


**** SECTION 8 - EXPOSURE CONTROLS, PERSONAL PROTECTION ****

Engineering Controls:
Safety shower and eye bath. Mechanical exhaust required.

Personal Protective Equipment

Eyes:
Wear appropriate protective eyeglasses or chemical safety goggles
Skin:
Wear appropriate protective gloves to prevent skin exposure.

Respirators:
Follow the OSHA respirator regulations found in 29 CFR 1910.134 or European Standard EN 149. Always use a NIOSH or European Standard EN 149 approved respirator when necessary.


**** SECTION 9 - PHYSICAL AND CHEMICAL PROPERTIES ****

Physical State:			Solid
Color:					powder
Odor:					N/A
pH:						N/A
Viscosity:				N/A
```

**Boiling Point:**	459^o^C/760 mmHg (cal)

```
Melting Point:				N/A
Autoignition Temperature:	N/A
```

**Flash Point:**	231^o^C (cal)

```
Explosion Limits:			Product does not present an explosion
hazard
Specific Gravity/Density:	N/A


**** SECTION 10 - STABILITY AND REACTIVITY ****

Chemical Stability: stable at recommend conditions
Incompatibilities with Other Materials:
Oxidizing agents, strong acids
Hazardous Decomposition Products:
Carbon monoxide, carbon dioxide, nitrogen oxides
Hazardous Polymerization:
Has not been reported.


**** SECTION 11 - TOXICOLOGICAL INFORMATION ****

Signs and symptoms
Irritant to skin, eye and mucous membranes
LD50/LC50: Not available.
Carcinogenicity:
Not listed by ACGIH, IARC, NIOSH, NTP, or OSHA.

Toxicological properties of the compound have not been fully determinate.


**** SECTION 12 - ECOLOGICAL INFORMATION ****

No data available


**** SECTION 13 - DISPOSAL CONSIDERATIONS ****

Dispose of in a manner consistent with federal, state, and local regulations.


**** SECTION 14 - TRANSPORT INFORMATION ****

IATA         Non-hazardous for air transport
IMDG         Non-hazardous for sea transport
RID/ADR      Non-hazardous for road transport


**** SECTION 15 - REGULATORY INFORMATION ****

International Regulations:
Hazard Symbols: Xi Irritant
Risk Phrases:
R 36/37/38 - Irritating to eyes, respiratory system and skin.
Safety Phrases:
S 26 - In case of contact with eyes, rinse immediately with plenty of water and seek medical advice.
S 36/37/39 - Wear suitable protective clothing, gloves and eye/face protection.
WGK (Water Danger/Protection):
No information available.
Information about limitation of use
It is for research and development use only.


**** SECTION 16 - ADDITIONAL INFORMATION ****

MSDS Creation Date: 01.02.2018   Revision #0 Date: Original.

The information above is believed to be accurate and represents the best information currently available to us. However, we make no warranty of merchantability or any other warranty, express or implied, with respect to such information, and we assume no liability resulting from its use. Users should make their own investigations to determine the suitability of the information for their particular purposes. In no way shall the company be liable for any claims, losses, or damages of any third party or for lost profits or any special, indirect, incidental, consequential or exemplary damages, howsoever arising, even if the company has been advised of the possibility of such damages.
```
